# Soft materials nanoarchitectonics: liquid crystals, polymers, gels, biomaterials, and others

**DOI:** 10.3762/bjnano.16.77

**Published:** 2025-07-04

**Authors:** Katsuhiko Ariga

**Affiliations:** 1 Research Center for Materials Nanoarchitectonics, National Institute for Materials Science (NIMS), 1-1 Namiki, Tsukuba 305-0044, Japan; 2 Graduate School of Frontier Sciences, The University of Tokyo, 5-1-5 Kashiwa-no-ha Kashiwa 277-8561, Japan

**Keywords:** biomaterials, gel, liquid crystal, nanoarchitectonics, polymer, soft materials

## Abstract

The concept of nanoarchitecture, as a post-nanotechnology methodology, can be defined as the construction of functional materials from nanometer-sized units using a variety of materials processes. It is believed to be particularly well suited to the assembly of soft materials that exhibit flexible and diverse structures and properties. To demonstrate its effectiveness, this review takes typical soft materials, including liquid crystals, polymers, gels, and biological materials, as examples. The aims are to extract the properties that emerge from them and to highlight the challenges that lie ahead. The examples also illustrate the potential applications, including organic semiconductor devices, electrochemical catalysts, thin-film sensors, solar energy generation, plastic crystal electrolytes, microactuators, smart light-responsive materials, self-repairing materials, enzyme cascade sensors, healing materials for diabetic bone defects, and bactericidal materials. As can be seen from these examples, soft materials nanoarchitectonics offers a wide range of material designs, specific functions, and potential applications. In addition, this review examines the current state and future of soft materials nanoarchitectonics. As an overall conclusion, it is highly anticipated that soft materials nanoarchitectonics will continue to develop significantly in the future.

## Introduction

The growing interest in soft and responsive materials, collectively known as “soft materials” [1–5], may be indicative of the significant advancements made in materials science. In the past, materials that were robust, rigid, and durable were esteemed. Nevertheless, the significance of materials endowed with intelligent capabilities that exhibit diverse responses to external stimuli is on the rise [6–10]. The field of materials chemistry is undergoing a transition from a focus on hard, robust materials to a greater emphasis on softer, more mouldable substances. This shift is actually reasonable as observed in the evolution of living organisms. From an alternative standpoint, living things can be regarded as sophisticated soft functional systems, wherein multiple functions operate softly in concert and are highly efficacious [11–15]. With the exception of the skeleton, they can be considered to be almost soft materials systems. It can be reasonably proposed that the mastery of materials science in the context of soft functional materials will facilitate the development of advanced functional systems that emulate the characteristics of living organisms. This concept is not limited to applications in biology, such as biomedical applications [15–20], but also extends to functional material systems that address issues such as energy [21–25] and environment [26–30].

The creation of such soft functional materials has been fostered alongside the advancement of science disciplines such as organic chemistry [31–35], polymer chemistry [36–40], and materials chemistry [41–44]. Consequently, soft materials, including polymers [45–49], liquid crystals [50–54] and gels [55–59], have been developed. Technologies that facilitate the conversion of these materials, not limited to typical soft materials, into more advanced assemblies include self-assembly via supramolecular chemistry [60–64], the creation of metal-organic frameworks (MOFs) through coordination chemistry [65–69], and the synthesis of covalent organic frameworks via polymer chemistry [70–74]. In particular, some thin-film techniques based on interface science are useful for the deliberate and rational assembly of ordered aggregated membrane structures. This is exemplified by the development of methods such as self-assembled monolayers (SAMs) [75–79], Langmuir–Blodgett (LB) method [80–84], and layer-by-layer (LbL) assembly [85–89]. The science and technology of creating and structuring soft materials has advanced in parallel with the growing demand for the development of more sophisticated functional materials.

Concurrently with the transition from hard to soft, there is also a significant trend in the advancement of materials sciences from the macroscale to the nanoscale. In other words, the ability to control the structures of materials at the nanoscale (i.e., atomic and molecular) is becoming increasingly important in the development of functional materials [90–94]. Even for the same material, alterations to its internal or assembly structure can result in significant changes to its functionality. Furthermore, as evidenced by quantum materials such as quantum dots [95–99], the nanoscale of size and dimensions can result in properties that are not attainable with bulk materials. In other words, the key to developing functionality and improving properties lies in controlling the nanostructure in addition to the creation of the materials themselves.

The advent of nanotechnology has facilitated the ability to access actual nanostructures. The latest advances in nanotechnology have made it possible to observe structures at the atomic and molecular level [100–104], to manipulate them [105–109], and to evaluate the physical properties of these nanostructures and nanospaces [110–114]. Concurrently, significant developments in the fabrication of functional materials and devices through microfabrication and nanofabrication have also contributed to technological innovation [115–119]. The advances in nanotechnology and the integration and functionality of objects in materials science have developed independently, yet they share a similar meaning. It is now appropriate to consider them together.

**Figure 1:**
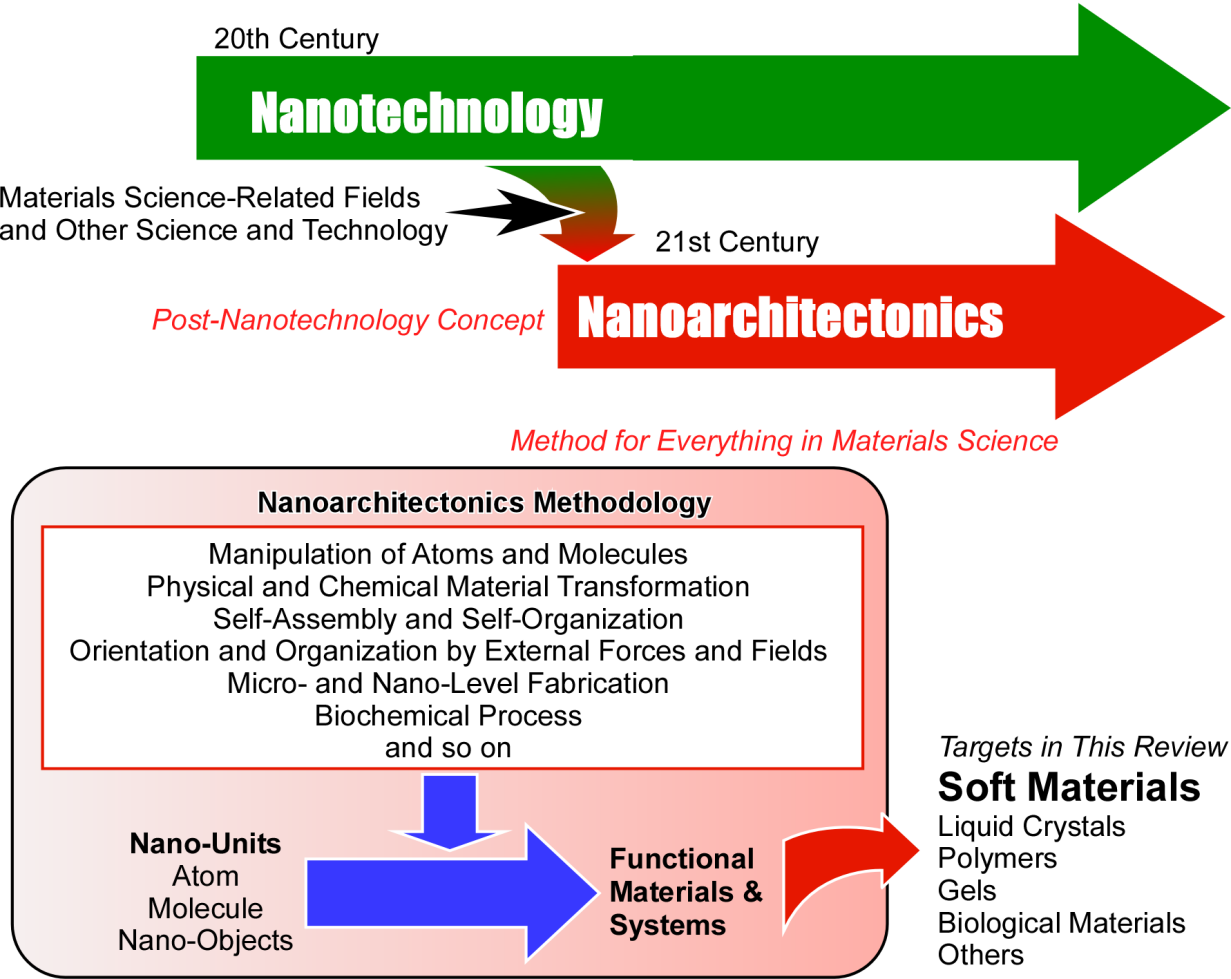
Outline of nanoarchitectonics concept: construction of functional materials from nanoscale units using a variety of materials processes.

The concept of nanoarchitectonics, defined as a post-nanotechnology methodology [120], fulfils this role. Similarly as Richard Feynman established the foundations of nanotechnology in the mid-20th century [121–122], Masakazu Aono proposed the concept of nanoarchitectonics [123–124] at the threshold between the 20th and 21st centuries. Nanoarchitectonics can be defined as the construction of functional materials from nanoscale units using a variety of materials processes (Figure 1) [125]. It is evident that the integration of knowledge and technology from both nanotechnology and materials sciences is imperative. Nanoarchitectonics may be regarded as a convergence of nanotechnology and materials sciences [126–128]. The construction of functional materials is achieved through the utilization of atomic, molecular, and nanoscale constituents, employing a range of techniques derived from the fields of nanotechnology and materials science [129]. The architectonics of functional materials is achieved through the selection and combination of a range of techniques, including the manipulation of atoms and molecules, physical and chemical material transformation, self-assembly and self-organization, orientation and organization by external forces and fields, micro- and nanoscale fabrication, and biochemical processes. The nanoarchitectonics method is particularly suited to the creation of asymmetric and hierarchical structures [130] due to the wide variety of processes that can be employed. In principle, all materials are composed of atoms and molecules, and thus, this methodology can be applied to the creation of all materials. If the ultimate theory of physics is the theory of everything [131], then nanoarchitectonics could be said to be the method for everything in materials science [132–133].

As previously outlined, nanoarchitectonics represents the convergence of nanotechnology and materials sciences, encompassing a vast array of disciplines. These include, but are not limited to, both hard and soft materials. The research objectives and applications of nanoarchitectonics are diverse. The term “nanoarchitectonics” has been used in the titles of numerous publications across a range of disciplines. They range from the creation of functional materials [134–138] and the control of structures [139–143], elucidation and understanding of physical phenomena [144–148], and applications in the field of fundamental biochemistry [149–153]. Nanoarchitectonics is also used in the development of chemical catalysts [154–158], photocatalysts [159–163], sensors [164–168], biosensors [169–173], devices [174–178], solar cells [179–183], fuel cells [184–188], batteries [189–193], supercapacitors [194–198], and other energy applications [199–203]. Furthermore, it is employed in environmental remediation [204–208], drug delivery [209–212], tissue engineering [213–217], and medical applications [218–222].

It seems reasonable to posit that nanoarchitectonics will make a significant contribution to the field of soft materials science. This is due to the fact that the methods employed in nanoarchitectonics are highly flexible. Thus, it is believed to be particularly well suited to the assembly of soft materials that display flexible and diverse structures and properties. Furthermore, it is intriguing to examine the advancements in materials sciences, spanning from hard to soft and from the macroscale to the nanoscale, upon relations with soft materials and nanoarchitectonics. However, the diversity of soft materials is such that it is virtually impossible to provide a comprehensive overview within a small review article. Accordingly, this review takes typical soft materials, including liquid crystals, polymers, gels, and biological materials, as examples, and selects and explains papers that claim to describe nanoarchitectonics in those fields or papers that are suitable for supplementing them. Additionally, it includes several examples that do not fall within the aforementioned categories but demonstrate distinctive innovation. It is not the intention of this review to encompass all fields and examples. Rather, it aims to select a number of examples, extract the characteristics that emerge from them, and highlight the challenges that lie ahead. These examples are roughly categorized by types of materials and their assemblies that are further grouped with application types. However, materials, assemblies, and applications are always integrated, and thus flows and orders of the examples are not simple. In addition, research progresses on liquid crystals, polymers, gels, and biological materials have different features that cannot be unified in one way. Therefore, this review emphasizes their varieties and diversities rather than summarizing in simply unified story patterns. By employing such methods, it will examine the present state and future of soft materials nanoarchitectonics.

## Review

### Liquid crystal nanoarchitectonics

Liquid crystals are an attractive form of soft materials, characterized by a combination of moderate fluidity and orientation [223–225]. They exhibit a high degree of diversity with regard to both the type of phase and the size of the regular structures. Furthermore, they include a high degree of responsiveness to external stimuli. The nanoarchitectonics of liquid crystal structures offers significant potential for the development of stimuli-responsive materials and functional substances, which would be highly beneficial for advancing materials science. The following section presents a selection of research studies that illustrate the potential of liquid crystals as a promising avenue of investigation in the field of soft materials nanoarchitectonics.

**Figure 2:**
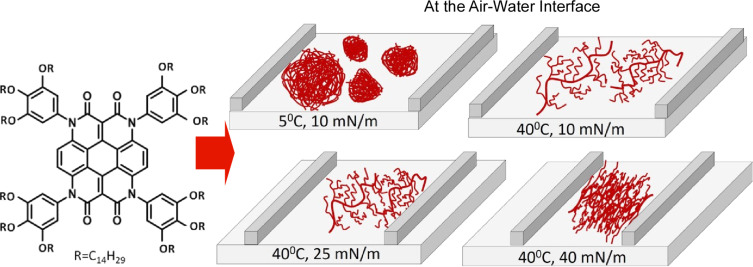
Nanoarchitectonics of discotic liquid crystal molecular nanowires by manipulating the subphase temperature and surface pressure in a Langmuir monolayer system at the air–water interface. Figure 2 was adapted with permission from [226], Copyright 2024 American Chemical Society. This content is not subject to CC BY 4.0.

Discotic liquid crystal molecules are constituted of flat discotic cores attached to flexible aliphatic alkyl chains. The ability to control the assembly structures of these molecules is crucial for the development of a range of functional materials. It is frequently observed that discotic liquid crystal molecules assume a columnar phase as a consequence of π–π stacking between the π-conjugated discotic cores. The self-organization at the molecular film level offers significant potential for a range of applications. The precise separation and alignment of π-conjugated discotic liquid crystal molecular nanowires represents an attractive area of research. Kumar, Nayak, and colleagues investigated an approach to control the structure of discotic liquid crystal molecular nanowires by manipulating the subphase temperature and surface pressure in a Langmuir monolayer system (Figure 2) [226]. The LB technique represents a powerful methodology that allows for effective control over the assembly of molecular-sized thicknesses. At low temperatures, the molecular nanowires coalesce, whereas, at high temperatures, the nanowires separate to form interconnected networks. The compression of the thin-film structure on the water surface resulted in the transformation of the network into a compact and highly uniform monolayer. At a temperature of 5 °C, the molecules formed islands with a high degree of density. At 40 °C and 10 mN·m^−1^, a network of separated nanowires was observed. As the surface pressure increased, the separated nanowires exhibited a tendency to come closer together, ultimately forming a nanowire network. At a higher surface pressure of 40 mN·m^−1^, the nanowires exhibited a tendency to come closer together, resulting in the formation of a compact and uniform monolayer. The interfacial nanoarchitectonics method of separating nanowires will undoubtedly prove invaluable for the separation of other 1D organic nanosystems, including nanotubes, nanowires, and nanoribbons.

**Figure 3:**
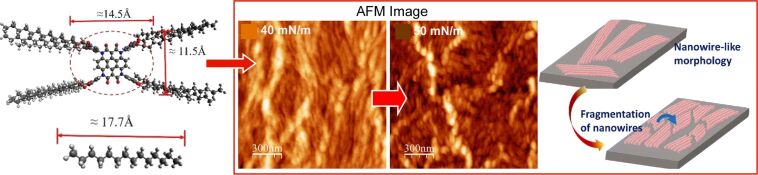
Effects of surface pressure on the molecular organization of a monolayer of heterocoronene-based discotic liquid crystals with transition from a closely packed nanowire domain to fragmented wire segments. Figure 3 was used with permission of The Royal Society of Chemistry, from [227] (“Deciphering pressure-induced nanoarchitectonics in a monolayer of heterocoronene-based discotics at air–water and air–solid interfaces” by N. Kumar et al., *Soft Matter*, vol. 19, issue 8, © 2023); permission conveyed through Copyright Clearance Center, Inc. This content is not subject to CC BY 4.0.

Mishra, Nayak, and colleagues conducted a comprehensive analysis of the effects of surface pressure on the molecular organization of a monolayer of heterocoronene-based discotic liquid crystals (Figure 3) [227]. The molecules were dispersed on water and formed floating domains at the air–water interface in the absence of applied pressure. The aggregates were constituted by small molecular units, with their alkyl chains oriented towards the air phase. Upon compression, the domains merged to form a coherent monolayer. The monolayer was observed to undergo irreversible structural changes, occurring through mechanisms such as loss of monolayer by desorption and local nucleation of defects. In terms of morphology, the nanoscale structure of the monolayer underwent a transformation from a randomly oriented nanowire configuration to a closely packed nanowire domain as the surface pressure increased. Ultimately, this transition culminated in the formation of fragmented wire segments that diffused locally on the film. These findings may offer insights into the fabrication of controllable nanoarchitectures of bipolar discotic liquid crystal molecules and their two-dimensional films. The structure and quality of the films are pivotal parameters for any molecular electronics application, thus making them indispensable for the development of nanoscale electronic devices. In particular, these findings on the controlled self-assembly of heterocoronene discotic liquid phase molecules can also serve as an excellent case study for intermolecular interactions in bulky molecular systems. Based on molecular nanoarchitectonics, this will facilitate the establishment of design rules for optimal performance in device applications.

**Figure 4:**
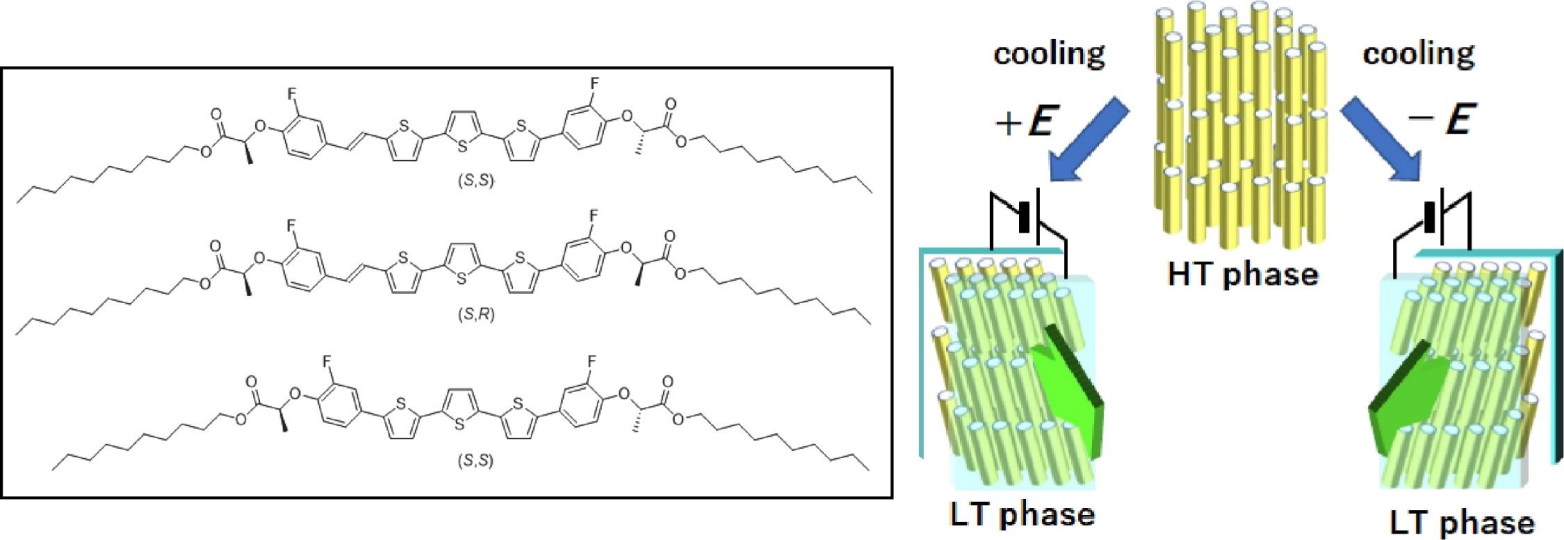
Chiral nanoarchitectonics with π-conjugated units to form a smectic crystal phase with the chromophore tilted at 45° by applying a DC bias during cooling from the high-temperature phase to the smectic crystal phase. Figure 4 was reproduced from [233] (Y. Matoba et al., “Diastereomeric Effect on Bulk Photovoltaic Property and Polarized Electroluminescence in Ferroelectric Liquid Crystals Containing an Extended π-Conjugated Unit”, *Bull. Chem. Soc. Jpn.*, 2023, vol. 96, issue 3, pages 247–256, https://doi.org/10.1246/bcsj.20230011); by permission of Oxford University Press on behalf of the Society. © 2023 The Chemical Society of Japan. This content is not subject to CC BY 4.0.

Extended π-conjugated compounds, including oligoacenes and oligothiophenes, exhibit excellent carrier transport properties in the aggregated state, rendering them promising candidates for use as organic semiconductors. It can be posited that liquid crystal compounds incorporating extended π-conjugated cores may be considered as liquid crystal semiconductors that exhibit anisotropic conduction of electronic charge carriers. The efficient transport of electronic carriers has been confirmed in nematic, chiral nematic, smectic, and columnar phases. It has been demonstrated that liquid crystals comprising extended π-conjugated units and polar moieties can exhibit ferroelectric behavior and electronic carrier transport properties. The corresponding materials have been the subject of investigation for potential applications in electroluminescent devices, field-effect transistors, and solar cells [228–232]. To investigate the potential of chiral nanoarchitectonics in such applications, Funahashi and colleagues synthesized two diastereomers comprising identical π-conjugated units [233]. The diastereomers exhibited disparate structural characteristics and properties (Figure 4). One of these diastereomeric molecules had a smectic crystal phase that had a tilted chromophore (the part that gives the molecule its color) at 45° from the layer normal. This tilted chromophore caused a macroscopic polarization (a change in the electric field) when a direct current (DC) bias was applied during cooling from a high-temperature phase to a smectic crystal phase. By contrast, the other diastereomer exhibited a smectic crystal phase in which the chromophore was parallel to the layer normal and no macroscopic polarization was induced. Bulk photovoltaic effects and polarization-induced electroluminescence were exclusively observed in the polarized smectic crystal phase with the chromophore tilted from the layer normal. It is conceivable that the bulk photovoltaic effect could result in the generation of an open circuit voltage that exceeds the bandgap of the active layer. Furthermore, the bulk photovoltaic effect in the tilted smectic crystal phase, with the addition of fullerene derivatives, was also investigated. The emission was linearly polarized with a dichroic ratio exceeding ten for the polarization-induced electroluminescence observed in the tilted smectic crystal phase. Reversing the polarity of the poling treatment enabled the axis of linearly polarized electroluminescence to be rotated by 90°. These findings suggest that the molecular chirality, in conjunction with the tilted orientation of the chromophores within the liquid crystal molecules, induces a macroscopic electric polarization, resulting in a bulk photovoltaic effect and polarization-induced electroluminescence.

**Figure 5:**
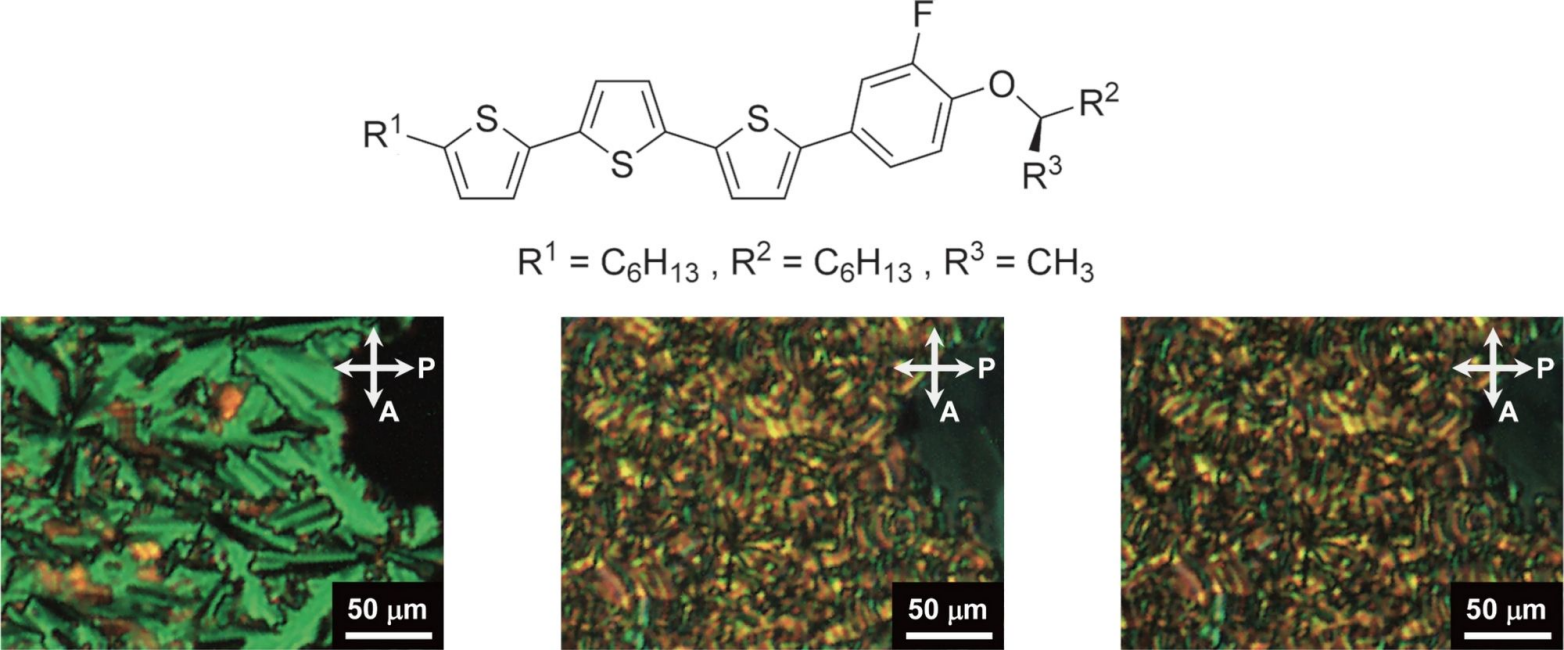
Liquid crystal compounds with phenyl terthiophene extended π-conjugation incorporating chiral, branched alkoxy chains to display ferroelectric chiral smectic C (at 142 °C, left image) and ordered smectic phases at 90 °C (middle image) and 40 °C (right image). Figure 5 was reproduced from [237] (A. Seki et al., “Ferroelectric Photovoltaic Effect in the Ordered Smectic Phases of Chiral π-Conjugated Liquid Crystals: Improved Current-Voltage Characteristics by Efficient Fixation of Polar Structure”, *Bull. Chem. Soc. Jpn.*, 2023, vol. 96, issue 11, pages 1224–1233, https://doi.org/10.1246/bcsj.20230185); by permission of Oxford University Press on behalf of the Society. © 2023 The Chemical Society of Japan. This content is not subject to CC BY 4.0.

The transport of both holes and electrons in various liquid crystal phases of semiconductors represents a fascinating area of research [234–236]. The use of liquid crystal semiconductors in the construction of lightweight organic electronic devices, including thin-film field-effect transistors, light-emitting diodes and solar cells, represents a promising avenue of research. Four liquid crystal compounds were synthesized by Seki et al., incorporating phenyl terthiophene-extended π-conjugation and chiral, branched alkoxy chains (Figure 5) [237]. The compounds exhibited ferroelectric chiral smectic C, and ordered smectic phases. The purpose of this research was to examine how these chiral units contribute to electrical functions. A specific, non-scattering photocurrent decrease was observed in the organized smectic phase of liquid crystal substances containing (*R*)-3-octyloxy groups as chiral components. A bulk photovoltaic effect driven by ferroelectric properties was observed in the polarized structure of the chiral ordered smectic phase. The effective stabilization of the polarized structure contributed to improving the output performance of the bulk photovoltaic effect. Observations were made in liquid crystal cells containing both compounds, revealing that the compounds within the chiral ordered smectic phase, in a polarized state, demonstrated the ferroelectric bulk photovoltaic effect, which resulted in a photocurrent with no applied bias. The reported outcomes can be beneficial for enhancing the performance of the ferroelectric bulk photovoltaic effect in organic ferroelectric π-conjugated compounds, thereby potentially accelerating the creation of next-generation organic thin-film sensors and photovoltaics.

**Figure 6:**
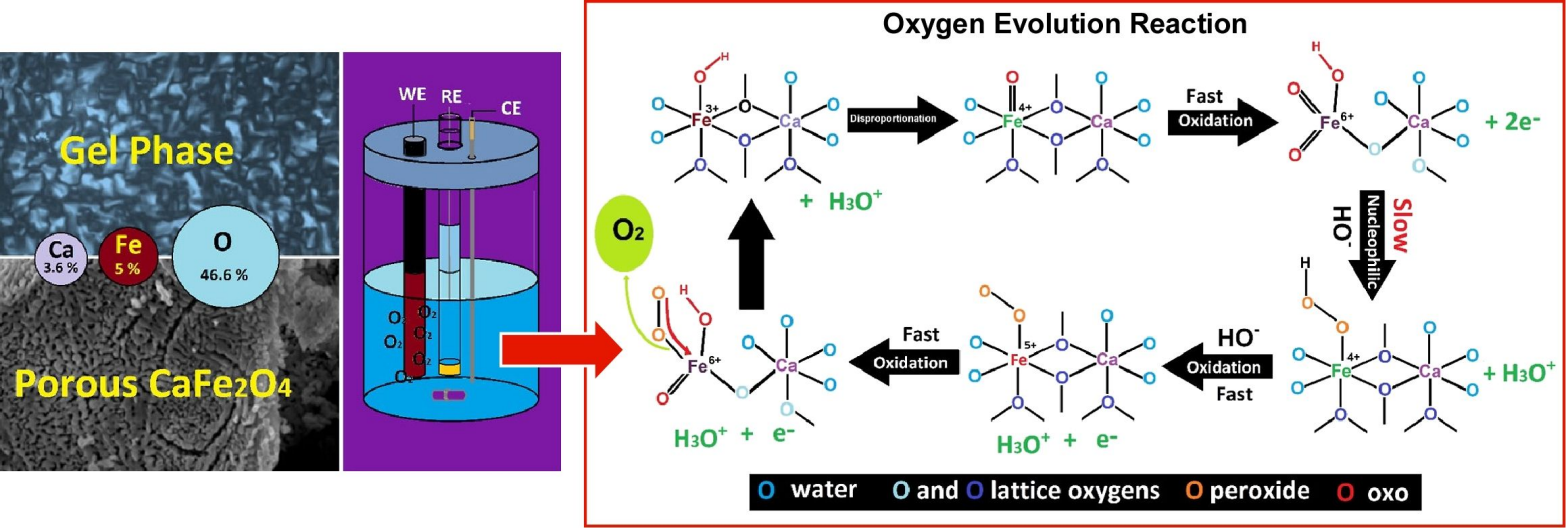
Nanoarchitectonics of CaFe_2_O_4_ thin-film electrodes on graphite rods via a molten salt-assisted self-assembly process (left) with excellent performance in the oxygen evolution reaction (right). Figure 6 was reproduced from [241] (© 2023 H. A. Raza et al., published by ACS, distributed under the terms of the Creative Commons Attribution 4.0 International License, https://creativecommons.org/licenses/by/4.0).

Liquid crystal nanoarchitectonics is not merely concerned with the manipulation of liquid crystal structures; it also encompasses the transfer of such structures to alternative materials, thereby influencing their nanostructure [238–240]. Metal oxides of earth-abundant elements are of significant importance in the fabrication of active electrodes for a range of electrochemical applications. However, it is not always the case that these materials adopt the desired nanostructure. Dag and colleagues fabricated CaFe_2_O_4_ thin-film electrodes on graphite rods via a molten salt-assisted self-assembly process (Figure 6) [241]. The presence of two salts and two surfactants ensures the stability of the lyotropic liquid crystal mesophase even at elevated salt concentrations. The calcium and iron salts act as eutectic solvents, facilitating the assembly of surfactants into the lyotropic liquid crystal phase. Additionally, they can be employed as precursors to produce CaFe_2_O_4_ thin films upon calcination. The resulting transparent solution is coated and subjected to calcination/annealing to form mesoporous thin films. The mesoporous films display excellent performance in the oxygen evolution reaction. It would be beneficial to extend the findings of this study to explore the nanoarchitectonics of other mesoporous metal oxides in electrocatalytic electrodes.

**Figure 7:**
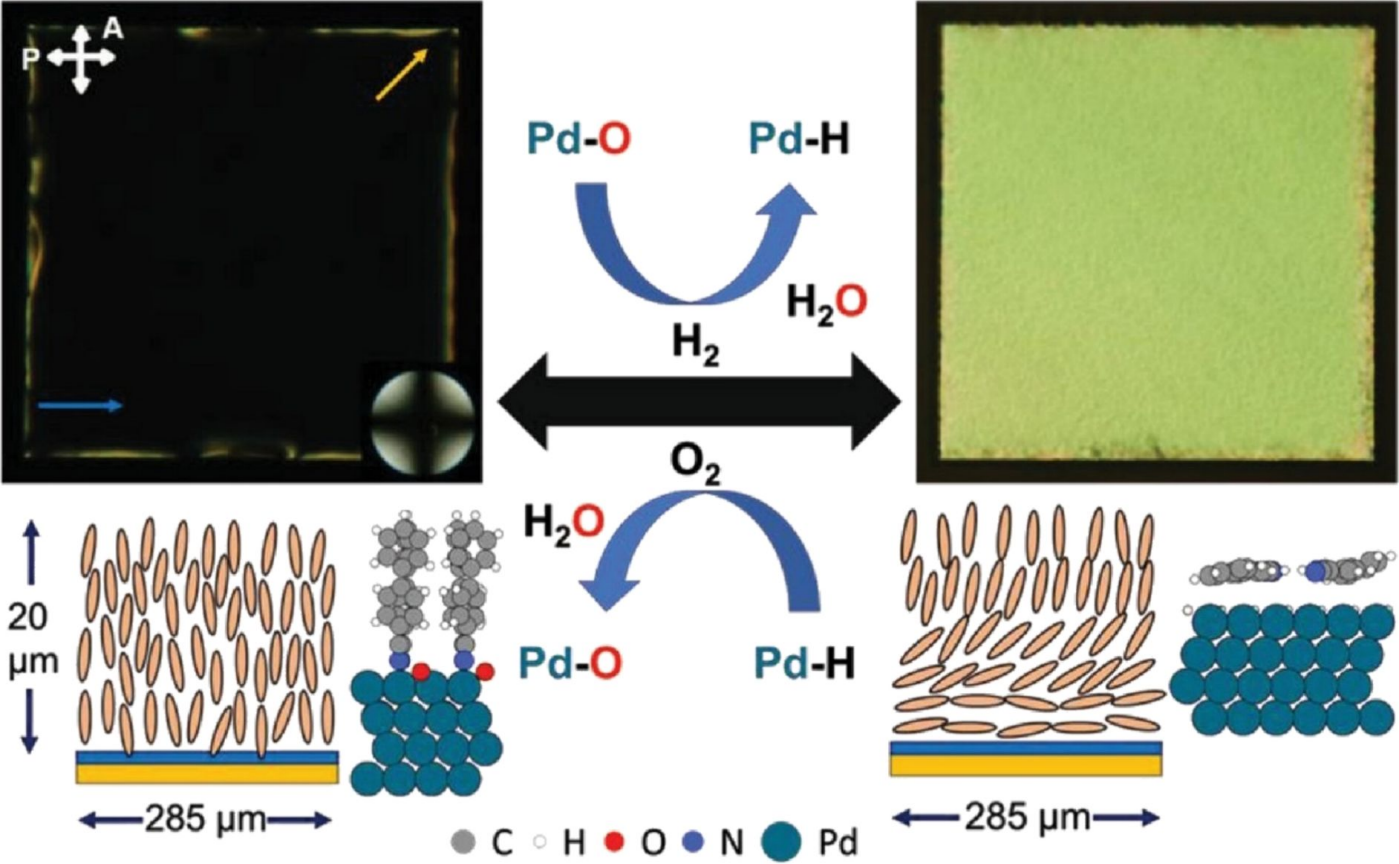
Rapid and reversible microactuation of liquid crystals based on the reaction of gaseous hydrogen and oxygen catalyzed at the Pd/Au surface. Figure 7 was reproduced from [242], H. Yu et al., “Actuating Liquid Crystals Rapidly and Reversibly by Using Chemical Catalysis”, *Advanced Materials*, with permission from John Wiley and Sons. Copyright © 2024 Wiley-VCH GmbH. This content is not subject to CC BY 4.0.

Additionally, there are nanoarchitectonics examples that couple liquid crystal structure control with other functions, such as catalysis. Mavrikakis, Abbott, and colleagues have reported the rapid and reversible microactuation of liquid crystals based on the surface catalysis of H₂ and O₂ at room temperature on a late transition metal alloy film (Figure 7) [242]. The reaction of gaseous hydrogen and oxygen, catalyzed by a Pd/Au surface, is employed to rapidly and reversibly alter the orientation of liquid crystals at room temperature. The dissociative adsorption of hydrogen on the palladium/gold film results in the reduction of pre-adsorbed oxygen and the generation of adsorbed hydrogen. This process causes the nitrile-containing liquid crystal to undergo a change in alignment, from vertical to planar. Subsequently, exposure to oxygen oxidizes the adsorbed hydrogen, reforming the adsorbed oxygen on the Pd/Au surface and restoring the liquid crystal to its initial alignment. The motion of the liquid crystal can be controlled by exposure to gas mixtures of H_2_ and O_2_ with varying compositions. In other words, chemical energy and catalysis can be used to reversibly move functional liquid crystals at the microscale. The chemical fuels’ energy density is significantly higher than that of current batteries, by at least two orders of magnitude, making chemically powered liquid crystal microactuators a promising option for actuation in autonomous systems such as microrobotics. Providing chemical energy to power mobile microsystems can potentially eliminate the requirement for intricate wiring that comes with electrical actuation.

**Figure 8:**
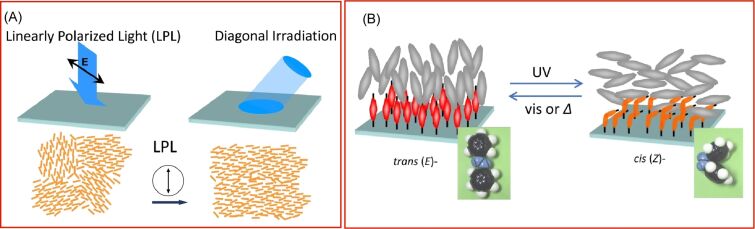
Intelligent photoresponsive materials systems utilizing azobenzene-containing monolayers and liquid crystal polymer films: (A) pronounced molecular orientation with the pronounced molecular cooperativity upon application of linearly polarized light; (B) the orientation of nematic liquid crystals over a micrometer-level thickness in the overlying liquid crystal cel upon the trans/cis photoisomerization of azobenzene monolayers. Figure 8 was reproduced from [243] (T. Seki et al., “Surface-mediated dynamic cooperative motions in azobenzene polymer films”, *Bull. Chem. Soc. Jpn.*, 2024, vol. 97, issue 1, bcsj.20230219, https://doi.org/10.1093/bulcsj/bcsj.20230219); by permission of Oxford University Press on behalf of the Society. © The Author(s) 2023. Published by Oxford University Press on behalf of Chemical Society of Japan. All rights reserved. This content is not subject to CC BY 4.0.

The hybrid nanoarchitectonics of liquid crystals and stimuli-responsive molecules or polymers represents a promising avenue for the development of soft functional materials. In particular, the incorporation of photoswitchable molecules and polymer systems can result in the creation of intelligent photoresponsive materials. In a recent review, Seki presented an overview of research activities utilizing azobenzene-containing monolayers and liquid crystal polymer films [243]. The linking of azobenzene molecules to liquid crystal and polymer systems has enabled the realization of a number of fascinating new motional functions. The use of photostimulation is advantageous in terms of functionality, as it enables the generation of multiple types of information simultaneously without the need for direct contact. When linearly polarized light is applied, the excitation of condensed molecules can be directional. The orientation of molecules can be induced by directional photostimulation. The utilization of liquid crystal materials results in the manifestation of pronounced molecular orientation, which is attributable to the pronounced molecular cooperativity, thus giving rise to a substantial orientation order (Figure 8A). Furthermore, the trans/cis photoisomerization of azobenzene monolayers can be amplified to the material level. Azobenzene molecules present at the molecular level on a substrate can alter the orientation of nematic liquid crystals over a micrometer-level thickness in the overlying liquid crystal cell (Figure 8B). As a consequence of this substantial molecular amplification, the surface is occasionally designated a “command surface” or “command layer”. Furthermore, other dynamic optical functions are being investigated, including photomechanical motion utilizing block copolymer monolayers, morphological switching, photoinduced mass transfer, and polymer motion via Marangoni flow. It is possible to extend this series of photoresponsive properties to other photochromic molecules and polymers. This methodology will be of interest for a variety of smart molecular system devices and smart applications.

Plastic crystals are materials that exhibit both order and a flexible structure [244–247], similar to that observed in liquid crystals. Plastic crystals are composed of three-dimensional crystal lattices that are regularly aligned along the long molecular axis. However, at the level of molecular species or molecular ions, there is orientational and rotational disorder. These materials are distinguished by their high degree of plasticity and diffusivity. In the case of ionic compounds, this results in the formation of an ion-conducting phase, rendering them an intriguing material for use as a transport field for target ions. The following examples illustrate the potential of nanoarchitectonics based on plastic crystals.

**Figure 9:**
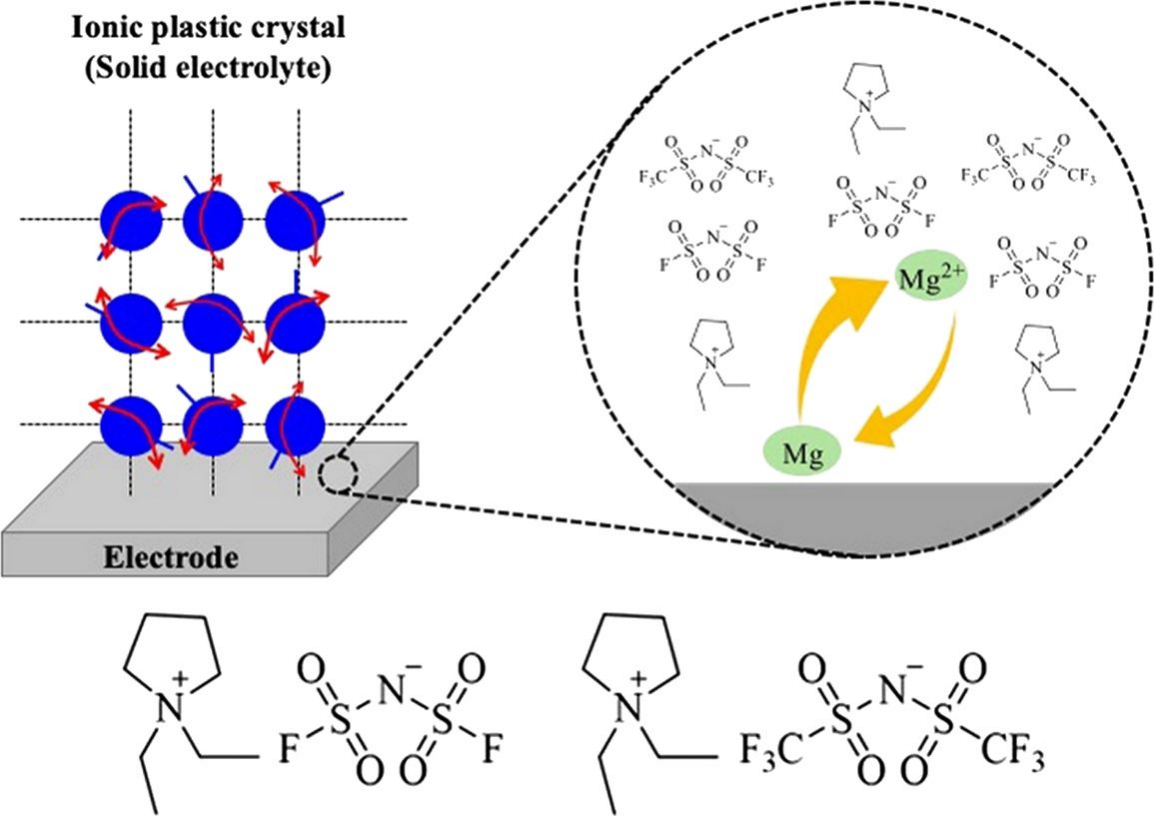
A solid electrolyte consisting of organic ionic plastic crystals with added magnesium salt for secondary Mg batteries where the utilization of the bis(fluorosulfonyl)amide anion is pivotal to the reversible redox reaction of Mg. Figure 9 was reproduced from [248] (© 2024 Y. Hirotsu et al., published by Oxford University Press on behalf of the Chemical Society of Japan, distributed under the terms of the Creative Commons Attribution-NonCommercial 4.0 International License, https://creativecommons.org/licenses/by-nc/4.0/). This content is not subject to CC BY 4.0.

Solid electrolytes can be used safely in secondary magnesium batteries but show lower ionic conductivity compared to conventional liquid electrolytes. A class of solid electrolyte known as organic ionic plastic crystals exhibits exceptional thermal stability, electrochemical stability, and ionic conductivity, making it a promising option. Yoshizawa-Fujita and co-authors investigated the impact of anion species and Mg salt concentration on the characteristics of pyrrolidinium-based organic ionic plastic crystals. The aim of this research was to create a new solid electrolyte made of organic ionic plastic crystals with magnesium salt incorporated for the use in next-generation secondary batteries (Figure 9) [248]. A series of organic ionic plastic crystal/Mg salt composites were prepared with varying magnesium salt concentrations, and their thermal and electrochemical properties were subsequently evaluated. The utilization of the bis(fluorosulfonyl)amide anion is pivotal to the reversible redox reaction of Mg. The employment of bis(fluorosulfonyl)amide as the anion in both the organic ionic plastic crystals and the Mg salt resulted in an enhancement of the redox properties. The bis(fluorosulfonyl)amide structure is well-suited to Mg electrochemistry and will be instrumental in the advancement of high-performance secondary Mg batteries.

As previously stated, organic ionic plastic crystals show promise as new organic solid electrolyte materials. However, their practical application remains elusive due to insufficient mechanical strength and ionic conductivity. Yoshizawa-Fujita and colleagues introduced a lithium salt, lithium bis(fluorosulfonyl)amide, and an inorganic solid electrolyte, Li_7_La_3_Zr_2_O_12_ (LLZO), into the organic ionic plastic crystal *N*,*N*-diethylpyrrolidinium bis(fluorosulfonyl)amide [249]. The fabricated organic–inorganic hybrid solid electrolyte underwent a series of evaluations, including thermal, mechanical, and electrochemical analyses, with the objective of elucidating the factors that influence the electrolyte properties. The findings demonstrated that, in general, the solids displayed remarkable thermal stability and exhibited high plasticity and ionic conductivity across a broad temperature range. It has been proposed that the formation of disordered interfaces of organic ionic plastic crystals/Li on LLZO particles enhances the ionic conductivity. In systems with disordered interfaces, an increase in the amount of additive results in a reduction in the distance between particles and a gradual interconnection of the interfaces, thereby enhancing the ionic conductivity. However, above the percolation threshold, the additive particles begin to overlap, resulting in the formation of isolated, disordered interfaces and an increase in the volume of less conductive material. Accordingly, the highest value of ionic conductivity is observed at moderate concentrations of LLZO. Furthermore, the incorporation of LLZO has been observed to enhance the nanoindentation stiffness of the composite. These findings suggest that the application of organic ionic plastic crystals as solid electrolytes is feasible when the concentrations of lithium salt and LLZO are optimized.

Liquid crystals are an appealing category of soft materials that exhibit a combination of moderate fluidity and order. Liquid crystals can serve as a fundamental component in the field of soft materials nanoarchitectonics, particularly in the development of stimuli-responsive materials. As seen in the represented examples, these materials possess flexible structures while exhibiting physical properties based on an ordered structure. Additionally, they are advantageous for expressing diverse dynamic physical properties, including stimuli responsiveness and carrier mobility. Liquid crystals and plastic crystals can serve as valuable design platforms for soft materials nanoarchitectonics.

### Polymer nanoarchitectonics

Polymers exhibit a range of mechanical properties, including those that render them malleable and pliant, thus making them suitable for utilization as soft materials. Moreover, there is a substantial history of polymer synthesis and development, and chemical design is also a viable avenue of research [250–254]. Additionally, a range of biopolymers can be derived from natural sources. Moreover, techniques such as LbL assembly can be employed to organize polymers and a range of other materials into layers [255–259]. Polymer materials are of significant value as components of soft materials, and can be employed in the creation of a vast array of structures. The following section will present examples of research into polymer-based soft materials nanoarchitectonics.

**Figure 10:**
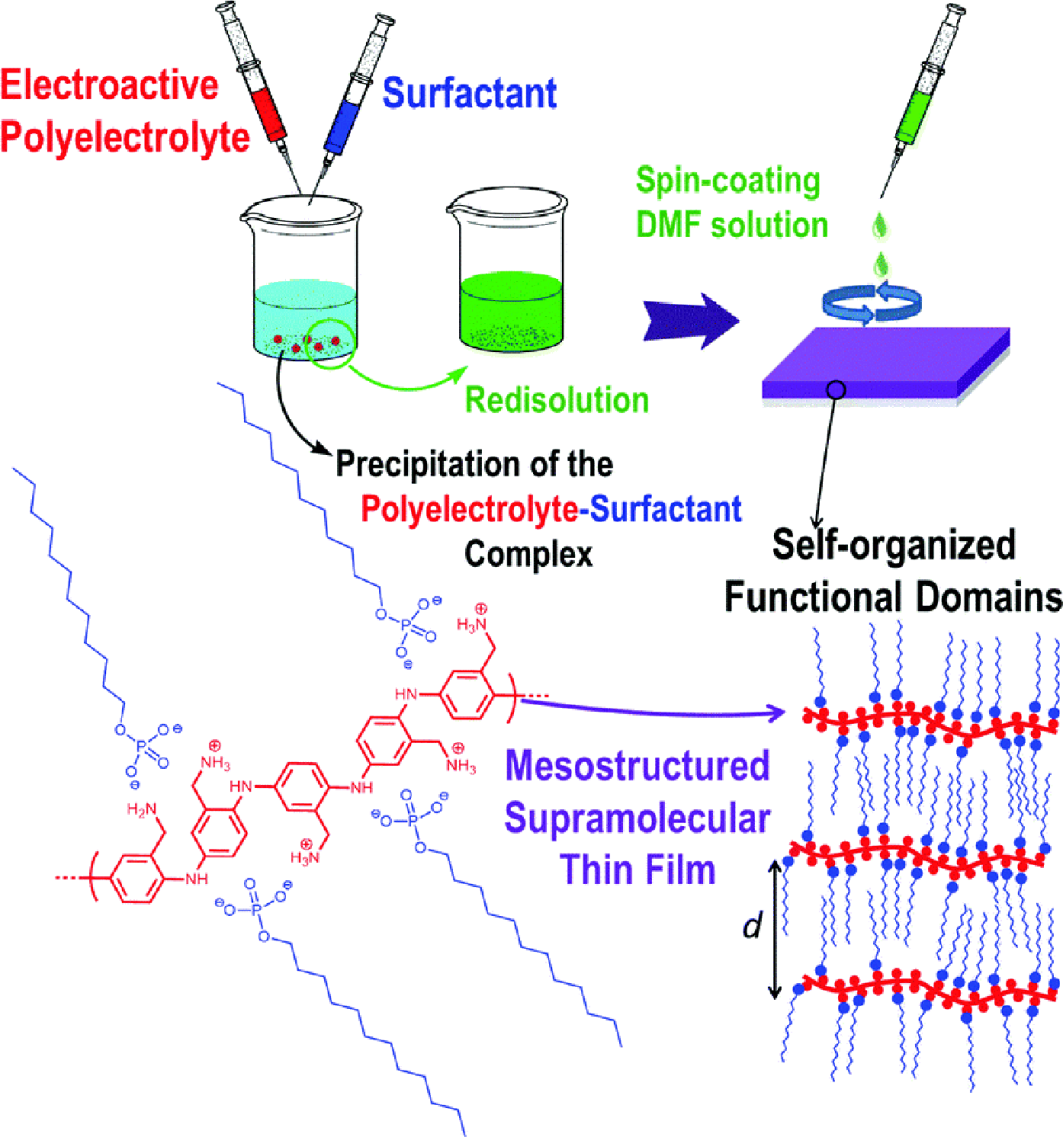
Electrochemical nanoarchitectonics for the chemical modification of electrode surfaces with self-assembled electroactive polyelectrolyte–surfactant complexes composed of amino-appended polyaniline and monododecyl phosphate. Figure 10 was used with permission of The Royal Society of Chemistry, from [260] (“Electrochemical nanoarchitectonics through polyaminobenzylamine–dodecyl phosphate complexes: redox activity and mesoscopic organization in self-assembled nanofilms” by A. Lorenzo et al., *Phys. Chem. Chem. Phys.*, vol. 20, issue 11, © 2018); permission conveyed through Copyright Clearance Center, Inc. This content is not subject to CC BY 4.0.

The development of films with mesoscopic levels of organization, which involve redox-active molecules, is a significant area of focus within the field of polymer nanoarchitectonics. These mesostructured materials have the potential to be used in a variety of applications, such as electrocatalysis, electronic devices, and electrochemical energy conversion and storage. Marmisollé and colleagues conducted research on “electrochemical nanoarchitectonics through polyaminobenzylamine–dodecyl phosphate complexes” and proposed a straightforward strategy for modifying electrode surfaces with self-assembled polyelectrolyte–surfactant complexes (Figure 10) [260]. The complex structures consist of polyaniline appended with amino groups and monododecyl phosphate. The intricate films were produced using a spin coating method. The films displayed distinct lamellar structures, resulting from the strong interactions between the phosphate groups of the complexes and the positively charged ammonium groups of the polyelectrolyte. The alignment of lamellae in a parallel orientation to the substrate is significantly influenced by the percentage of surfactant present. An increase in surfactant concentration results in the formation of more ordered, hydrophobic coatings, while the incorporated polymers retain their electroactive properties. Ion transport through the film is still possible. Nanoarchitectonics polymer composite films can incorporate bioelectroactive proteins such as glucose oxidase. The high hydrophobicity provides an optimal environment for membrane or membrane-associated proteins, thus paving the way for new bio-nanoarchitected devices. In particular, they can serve as electroactive components in sensing and energy devices.

**Figure 11:**
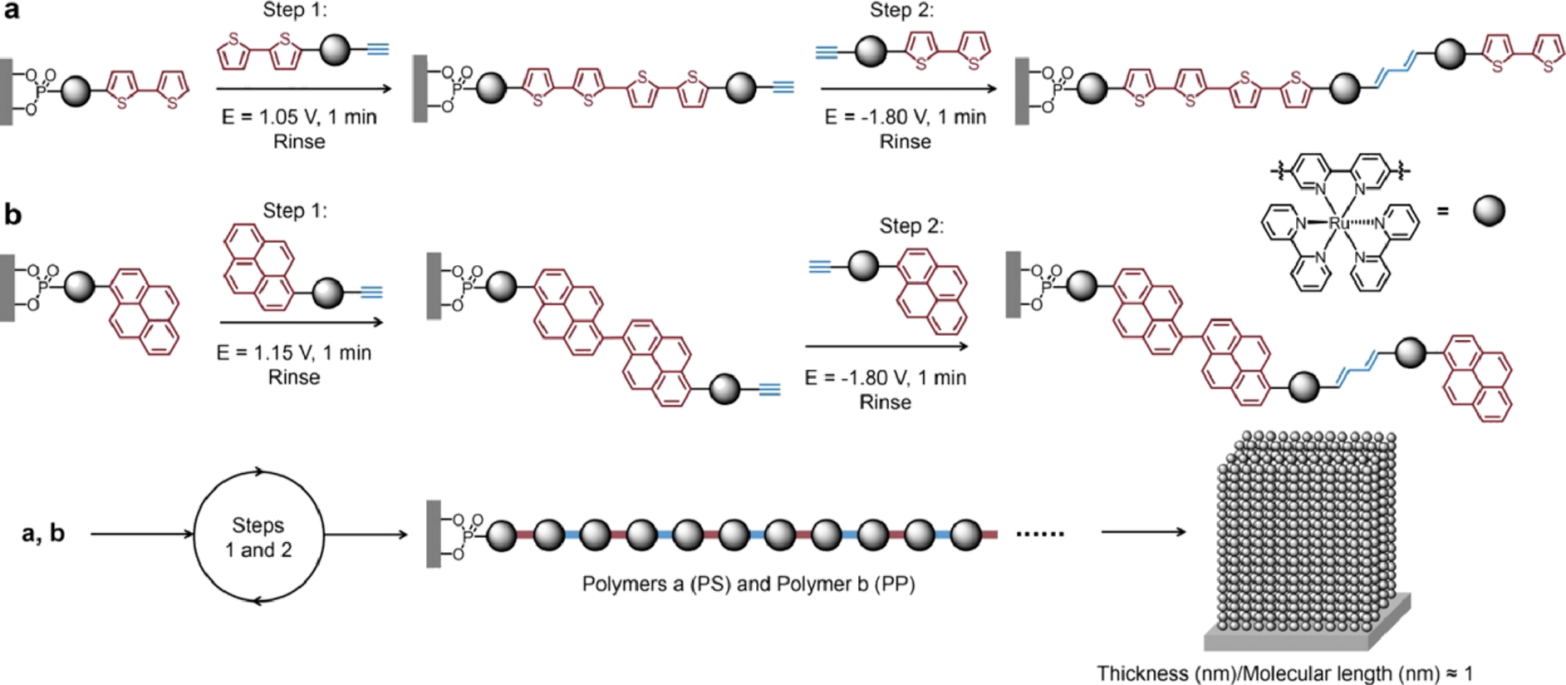
Nanoarchitectonics on electrosynthesis and assembly of conjugated metallopolymers to produce end-on conjugated metallopolymers through surface-initiated one-by-one electrochemical addition and assembly of bifunctional monomers with electroactive redox units under alternating positive and negative potentials. Figure 11 was reproduced from [263], Y. Li et al., “Nanoarchitectonics on Electrosynthesis and Assembly of Conjugated Metallopolymers”, *Angew. Chem. Int. Ed.*, with permission from John Wiley and Sons. Copyright © 2023 Wiley-VCH GmbH. This content is not subject to CC BY 4.0.

Furthermore, a unit-by-unit construction on solid surfaces to form end-on polymer arrays has been documented in the literature [261–262]. In contrast to edge-on and face-on orientations, end-on uniaxial conjugated polymers have the potential to provide macroscopic crystalline films. Nevertheless, the fabrication of these materials has proven challenging using conventional methods, largely due to the slow thermodynamic equilibrium. In response, Li and colleagues published a research paper entitled “Nanoarchitectonics on Electrosynthesis and Assembly of Conjugated Metallopolymers." In this method, they achieved nanoarchitectonics of end-on conjugated metallopolymers through surface-initiated one-by-one electrochemical addition and assembly of bifunctional monomers with electroactive redox units under alternating positive and negative potentials (Figure 11) [263]. The synthesis of uniaxial end-on conjugated metallopolymers in centimeter-sized domains was successfully achieved. SAMs of Ru complexes were employed as assembly templates to direct the topochemical addition of repeating monomers. The polymer is extended in discrete steps through the oxidation of units such as thiophene and pyrene, and the reduction of alkynyl units. The same reaction is then repeated in order to repair any unreacted sites on the SAM, thereby ensuring maximum repeat coverage on a dynamic and statistical basis. This nanoarchitectonics approach enables the assembly of polymer arrays on substrates with uniform sub-nanometer morphology, ultrahigh modulus, and high conductivity. The optical and electrical on/off response of the fabricated structures is length-dependent. For example, the switching current under light illumination increases with increasing molecular length and monolayer thickness. Furthermore, the technique has the potential to be automated, which would facilitate its use as an assembly or printing technique. Consequently, it will contribute greatly to the miniaturization of organic materials and devices, including computing, sensing, diodes, transistors, and optical switches.

**Figure 12:**
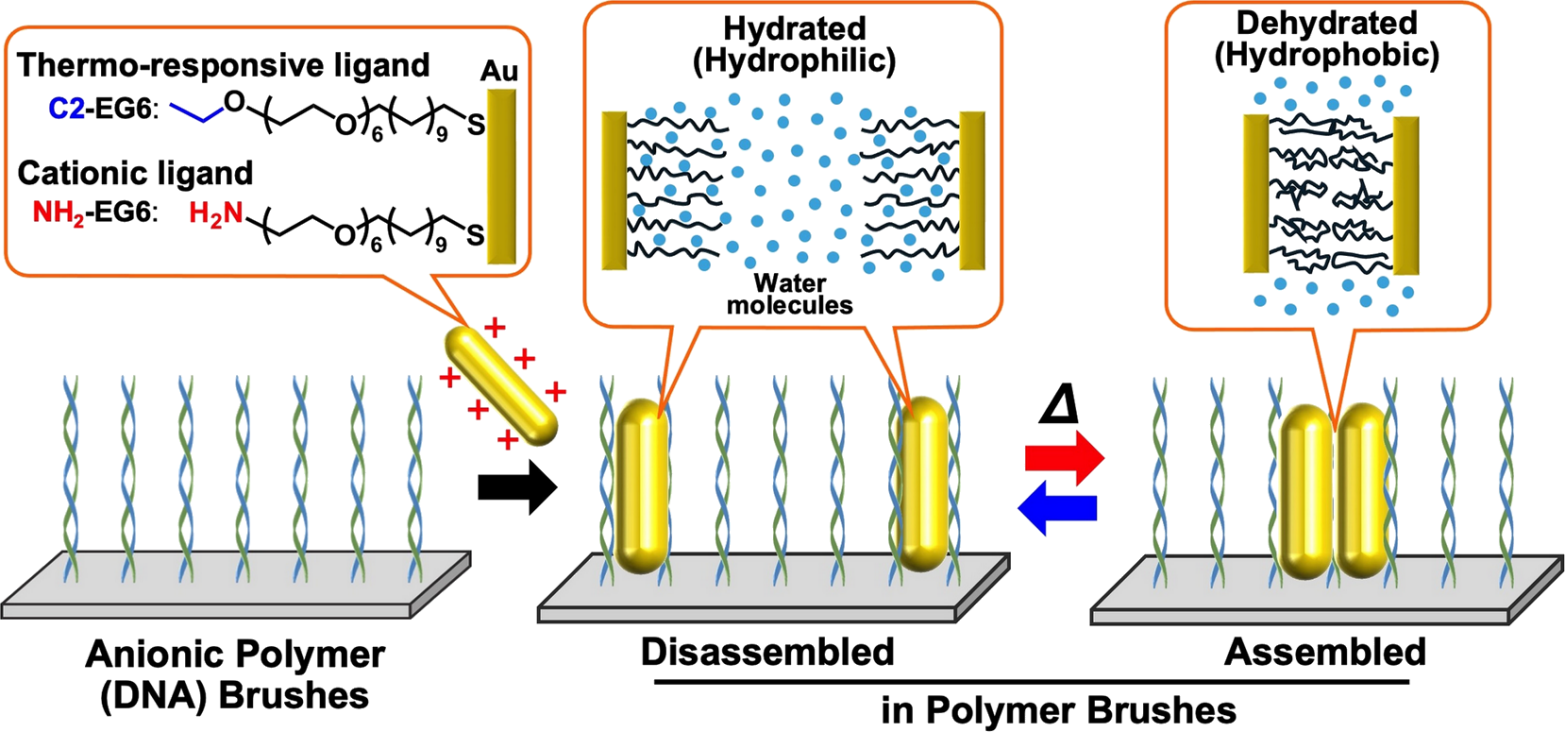
Nanoarchitectonics for the dynamic control of uniformly aligned thermoresponsive gold nanorods on solid substrates using polymer brushes in which, upon heating, the gold nanorods assemble within the polymer brushes, maintaining their vertical orientation and the system reverts to its original state by cooling. Figure 12 was reproduced from [264] (© 2024 J. Yang et al., published by Oxford University Press on behalf of the Chemical Society of Japan, distributed under the terms of the Creative Commons Attribution 4.0 International License, https://creativecommons.org/licenses/by/4.0).

The nanoarchitectonics of objects such as gold nanorods can also be performed with the assistance of polymer chains. Having precise control over the spatial arrangement of gold nanorods presents substantial benefits in the creation of plasmonic systems. Achieving dynamic control over the spatial configuration of gold nanorods is often quite difficult. Ijiro, Mitomo, and colleagues have proposed a strategy for the dynamic control of uniformly aligned thermoresponsive gold nanorods on solid substrates via the application of polymer brushes (Figure 12) [264]. Cationic gold nanorods and thermoresponsive gold nanorods are attached to anionic polymer brushes through moderate electrostatic forces, resulting in vertically aligned arrays of gold nanorods. The polymer brushes consist of alkyl-terminated hexaethylene glycol derivatives, functioning as thermoresponsive ligands. Upon heating, the gold nanorods assemble within the polymer brushes, maintaining their vertical orientation. Upon cooling, the system reverts to its original state. It was demonstrated that the gold nanorods are capable of significant lateral diffusion, exhibiting a considerable distance of movement. Hybrid materials comprising anisotropic nanoparticles and polymer brushes were designed and subjected to dynamic control. Consequently, rod-shaped gold nanoparticles have been the subject of considerable interest with regard to their potential use in optical antennas, sensitive biosensors, and medical applications. This is due to their strong plasmonic absorption in the visible–near infrared region, which is caused by electronic vibrations along the main axis. Given that the plasmonic properties of these particles are contingent upon their spatial configuration, it is similarly crucial to regulate the distance and orientation between the gold rod particles when designing plasmonic systems. This approach also offers a versatile platform for the development of advanced plasmonic devices.

**Figure 13:**
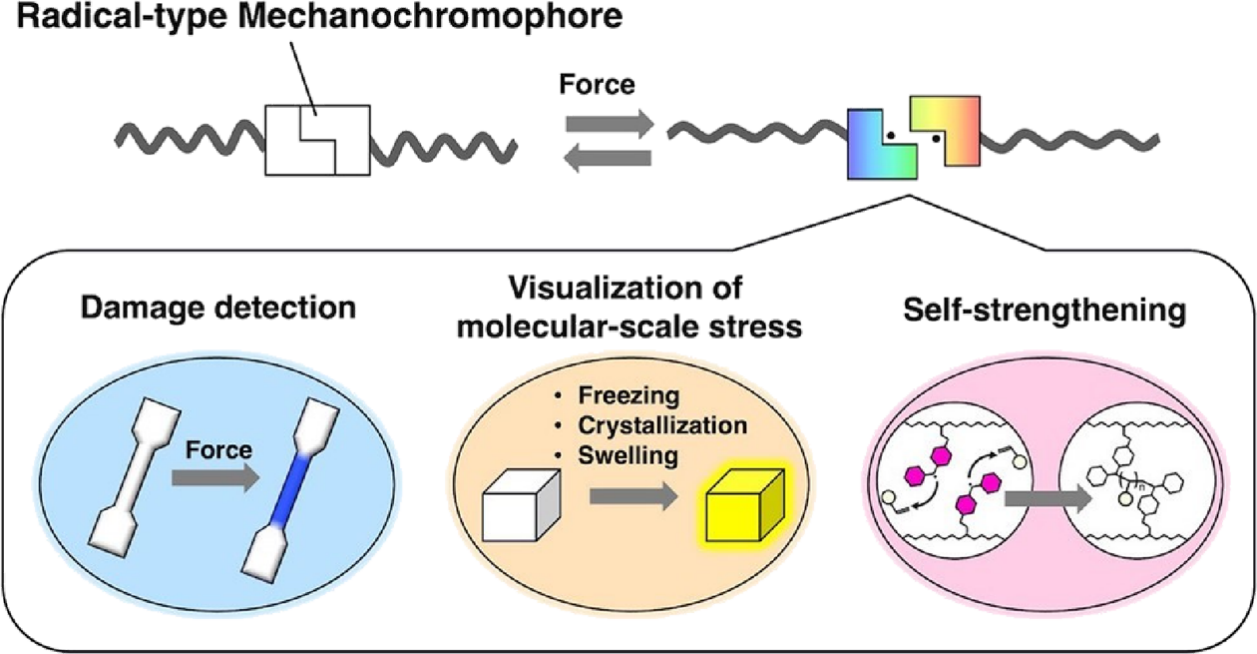
Nanoarchitectonics design of mechanochromic polymers based on radical dynamic covalent chemistry with the objective of imparting custom chromic properties to polymer materials. Figure 13 was reproduced from [274] (T. Yamamoto et al., “Mechanochromic polymers based on radical-type dynamic covalent chemistry”, *Bull. Chem. Soc. Jpn.*, 2024, vol. 97, issue 3, uoad004, https://doi.org/10.1093/bulcsj/uoad004); by permission of Oxford University Press on behalf of the Society. © The Author(s) 2023. Published by Oxford University Press on behalf of the Chemical Society of Japan. All rights reserved. This content is not subject to CC BY 4.0.

The field of polymer reactions has made significant advancements in the precise synthesis of desired polymer structures [265–269]. These polymers are anticipated to play a pivotal role in soft materials nanoarchitectonics. While the control of mechanical properties by polymer design has been a significant area of interest, mechanochemistry of polymers and related molecules has recently emerged as a field of growing scientific and industrial interests [270–273]. The development of mechanochromic polymers, which display optical changes in response to mechanical stress, is expected to have a substantial influence across multiple fields. Mechanical forces applied to materials can be visualized using these tools, and they can also be employed to aid in detecting damage, thus preventing significant harm to materials. They are capable of offering a diverse range of information, encompassing everything from the nanoscale to the macroscale. Otsuka and associates are involved in the precise design of mechanochromic polymers based on radical dynamic covalent chemistry, with the goal of conferring custom chromic properties to polymer materials. A recent review article discusses the characteristics and possible uses of radical chemistry (Figure 13) [274]. Radical dynamic covalent chemistry encompasses straightforward homolysis and radical–radical coupling reactions, which give rise to discernible color alterations without the necessity for catalysts or the generation of by-products. It is possible to develop a variety of dynamic covalent molecules that homolytically dissociate into stable radical species upon mechanical stimulation, thereby exhibiting color changes. The accumulated knowledge on molecular design to control the chemical stability of normally unstable radical species allows these mechanical functions to be manipulated as needed. The introduction of computational science has the potential to facilitate the creation of new radical mechanochromophores, which is expected to further advance radical dynamic covalent chemistry. Dynamic covalent polymers can undergo structural reorganization in response to specific stimuli, thereby enabling the control of their macroscopic states and viscoelastic properties. This capability allows for the development of a range of smart functionalities, including controllable swelling, degradability, self-repairing ability, and reworkability. It is anticipated that this will contribute to a number of research fields, including polymer degradation and recycling, the extension of the lifespan of polymer materials, the development of adhesive polymers for specific applications, and the exploration of new areas of polymer mechanochemistry.

**Figure 14:**
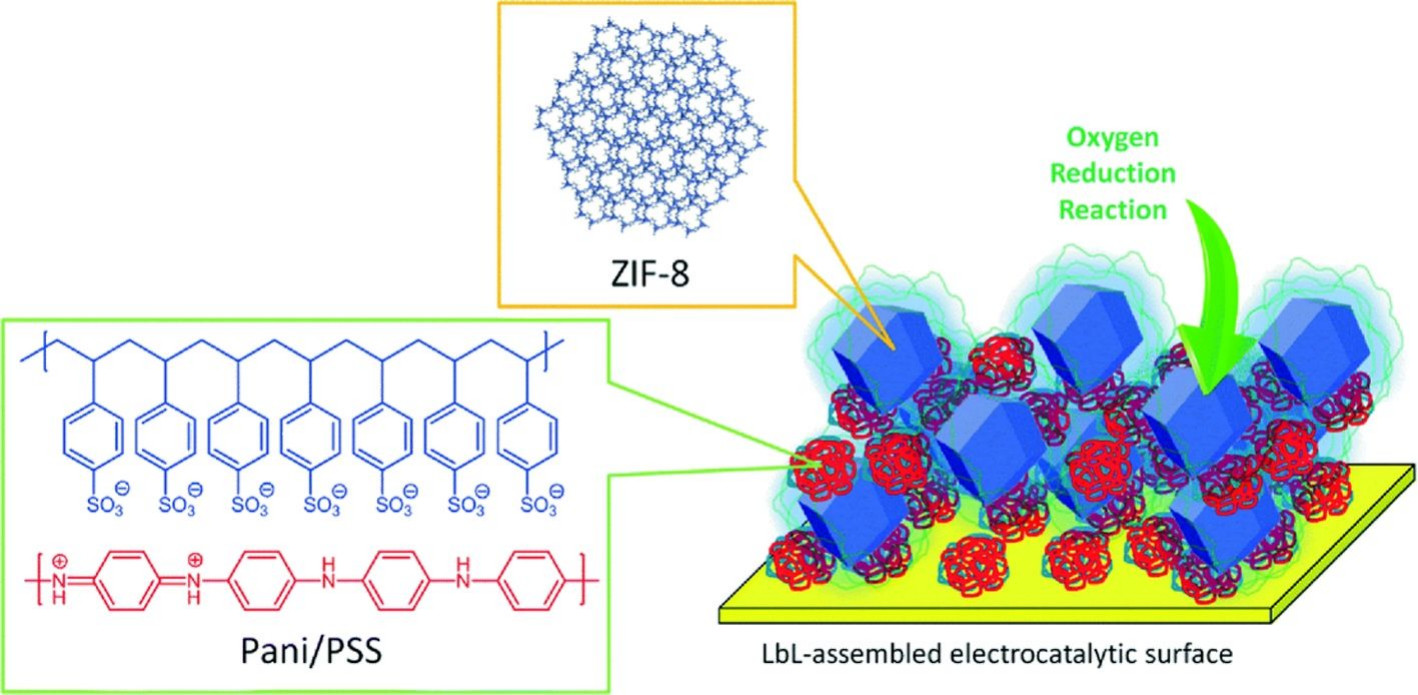
Oxygen reduction reaction catalysts with the sequential nanoarchitectonics of a colloidal suspension comprising polyaniline/polystyrene sulfonate and ZIF-8 metal-organic framework nanocrystals coated with polyallylamine hydrochloride. Figure 14 was used with permission of The Royal Society of Chemistry, from [275] (“Layer-by-layer integration of conducting polymers and metal organic frameworks onto electrode surfaces: enhancement of the oxygen reduction reaction through electrocatalytic nanoarchitectonics” by A. P. Mártire et al., *Molecular Systems Design & Engineering*, vol. 4, issue 4, © 2019); permission conveyed through Copyright Clearance Center, Inc. This content is not subject to CC BY 4.0.

Polymers, including polyelectrolytes, are pivotal constituents of LbL assembly. Additionally, they can serve as an important component in soft materials nanoarchitectonics, a flexible approach to assembling diverse functional materials in a layer-by-layer fashion. LbL methods provide a nanoarchitectonics approach to the construction of functional composite nanomaterials with exceptional electrocatalytic properties. Azzaroni, Rafti, Marmisollé, and colleagues optimized the electrocatalytic properties of a conducting polymer by synergistically combining it with an O_2_-absorbing MOF using LbL assembly [275]. The oxygen reduction reaction (ORR) represents a pivotal process in electrochemical energy conversion systems. Instead of costly Pt-based electrocatalysts, the potential of conducting polymers as ORR catalysts has been explored. This involved the sequential organization of a colloidal suspension comprising polyaniline/polystyrene sulfonate and ZIF-8 MOF nanocrystals coated with polyallylamine hydrochloride (Figure 14). This functional electrode was designed to combine two key elements, that is, high surface area and porosity with electroactivity. This combination is intended to result in enhanced ORR activity. The LbL-assembled films were electrically connected, thereby increasing the electrocatalytic current obtainable for ORR in aqueous environments with neutral pH. Furthermore, the selective incorporation and enrichment of oxygen within the MOF microporous matrix, which serves as an oxygen reservoir linked to the polymer electrocatalytic material, enhances the overall ORR activity. This exemplary work demonstrated the potential for a new method of fabricating MOF-based electrocatalytic surfaces. Further enhancements in performance may be attained by means of improvements in electron transport properties and optimization of the loading of the MOF within the LbL nanostructures.

**Figure 15:**
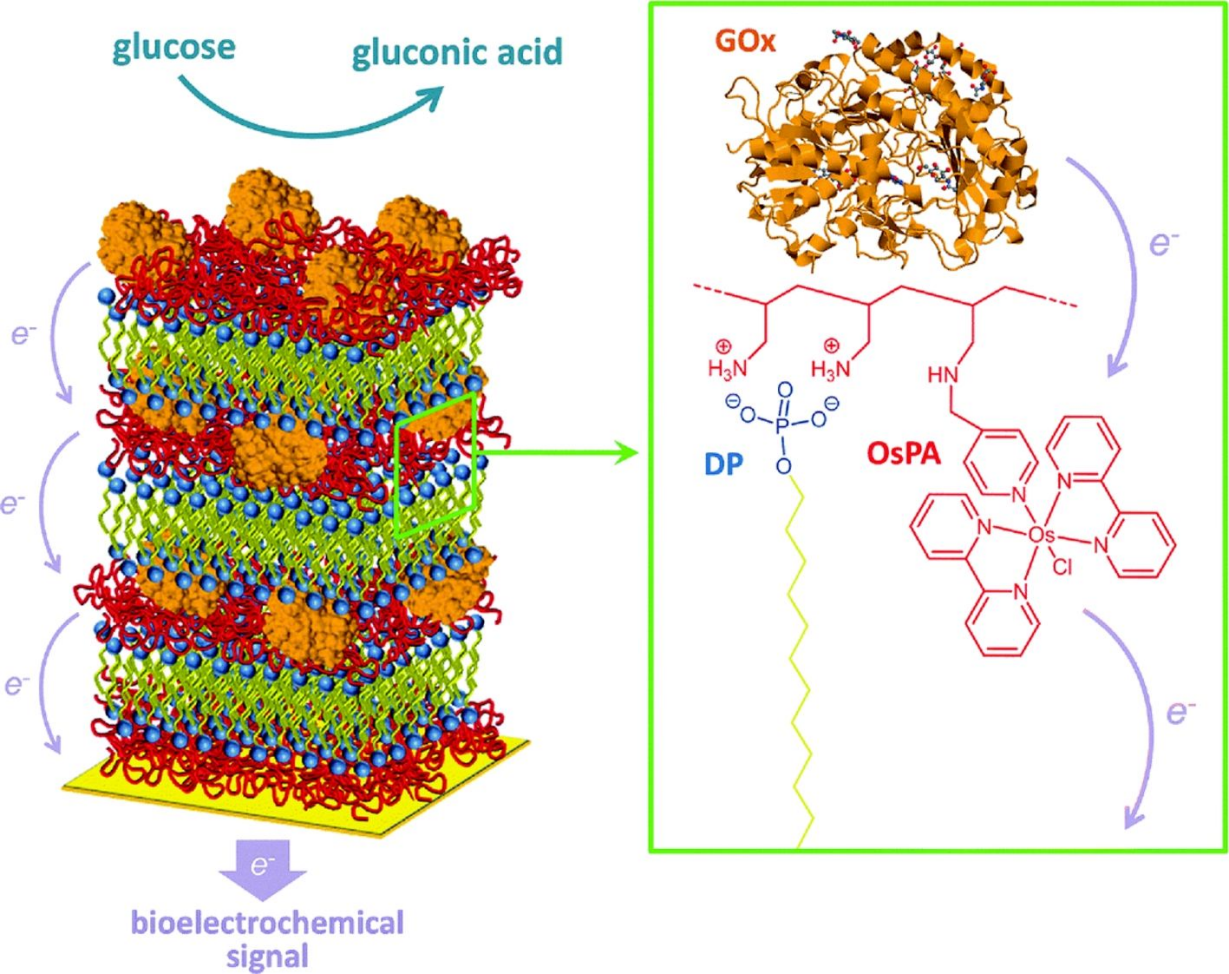
Bioelectrochemical nanoarchitectonics for the creation and functionalization of highly organized layered multilayer structures of lipid-like surfactants and polyelectrolytes in which, hydrophobic lamellar domains were employed as hosts to create electrochemically active films that display spatially addressed redox units, and redox-labelled polyallylamine and glucose oxidase were integrated into the layered hydrophilic domains. Figure 15 was used with permission of The Royal Society of Chemistry, from [276] (“Highly-organized stacked multilayers via layer-by-layer assembly of lipid-like surfactants and polyelectrolytes. Stratified supramolecular structures for (bio)electrochemical nanoarchitectonics” by M. L. Cortez et al., *Soft Matter*, vol. 14, issue 10, © 2018); permission conveyed through Copyright Clearance Center, Inc. This content is not subject to CC BY 4.0.

The creation of well-defined soft multilayer structures comprising a variety of components represents a crucial strategy in the field of soft materials nanoarchitectonics. Cornez, Azzaroni, and colleagues describe the preparation and functionalization of highly organized layered multilayer structures by layer-by-layer organization of lipid-like surfactants and polyelectrolytes (Figure 15) [276]. In this study, hydrophobic lamellar domains were employed as hosts to create electrochemically active films that display spatially addressed redox units. Furthermore, redox-labelled polyallylamine and glucose oxidase were integrated into the layered hydrophilic domains, thus forming the layered multilayer films. The multilayer films were fabricated by alternately depositing redox-active osmium complex-labelled polyallylamine hydrochloride, sodium dodecyl phosphate, and glucose oxidase in a sequential manner. The cooperation of bioelectrocatalysis in the presence of glucose and redox wiring within the layered multilayer films was demonstrated. The incorporation of an additional redox-active osmium complex layer between the surfactant bilayer and the glucose layer enhances the wiring efficiency of the redox assembly. The incorporation of lipid-like surfactants into polyelectrolyte multilayers can facilitate the development of soft materials nanoarchitectonics, enabling the versatile design of layered heterosupramolecular assemblies. This enables the sophisticated functionalization of the supramolecular organization of multicompartmental interfacial structures.

**Figure 16:**
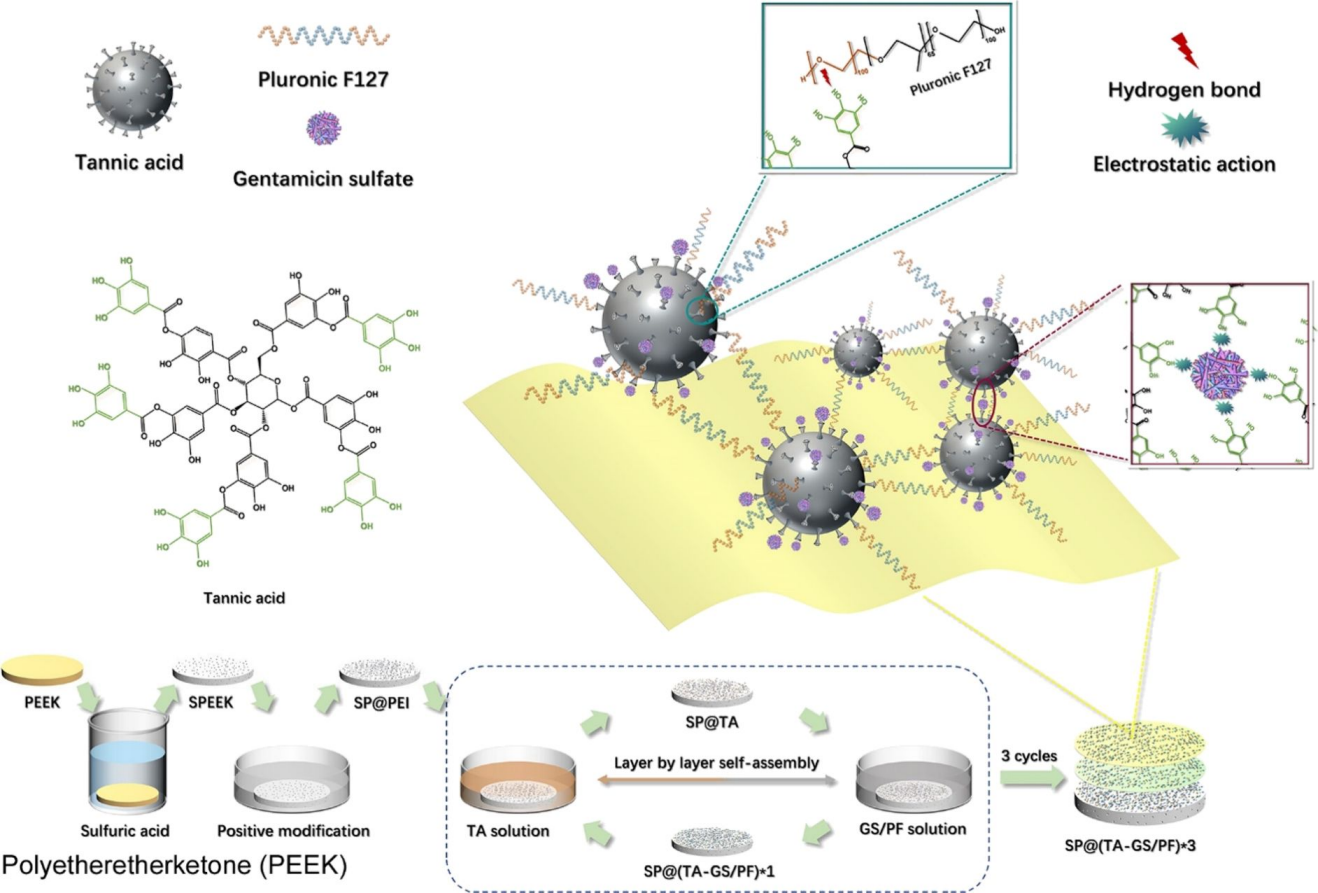
Layer-by-layer nanoarchitectonics involving the hybridization of tannic acid, gentamicin sulfate, and Pluronic F127 on a porous polyetheretherketone substrate prepared by sulfonation toward a bifunctional system of antioxidants and antibacterial properties on its surface and a model implant to effectively treat diabetic bone defects and restore the integration and remodelling of the implant and surrounding bone tissue. Figure 16 was adapted with permission from [277], Copyright 2022 American Chemical Society. This content is not subject to CC BY 4.0.

The fabrication of functional layered structures by means of soft materials nanoarchitectonics also makes a contribution to the field of medicine. For instance, patients with diabetic bone defects require novel and efficacious medical implant material strategies to enhance their prognosis. It is imperative to minimize the risk of implant failure due to excessive oxidative stress and the elevated risk of bacterial infection in patients with diabetes. Weng and colleagues employed an LbL construction strategy to enhance the healing ability of diabetic bone defects [277]. This involved the hybridization of tannic acid, gentamicin sulfate, and Pluronic F127 on a porous polyetheretherketone substrate prepared by sulfonation (Figure 16). The material, which exhibits a bifunctional system of antioxidants and antibacterial properties on its surface, was employed as a model implant to effectively treat diabetic bone defects and restore the integration and remodeling of the implant and surrounding bone tissue. In the process, polyetheretherketone is treated with H_2_SO_4_ to form a three-dimensional porous polyetheretherketone structure. Moreover, the porous polyetheretherketone was coated with tannic acid via an electrostatic interaction. Furthermore, the modified tannic acid was combined with gentamicin sulfate through electrostatic interaction and hydrogen bonding in a mixture of gentamicin sulfate and Pluronic F127. The prepared material demonstrated sustained antibacterial activity and facilitated osteoblast (MC3T3-E1) differentiation, which is essential for bone formation. Moreover, the material was observed to eliminate excessive oxidative stress, promote the growth of H_2_O_2_-injured human umbilical vein endothelial cells, and facilitate the secretion of endothelial growth factor, which is essential for angiogenesis. The subcutaneous implant model in diabetic rats and the bone tissue implant model yielded notable in vivo outcomes with respect to angiogenesis and osseointegration, respectively. The LbL strategy offers a highly applicable and versatile approach to soft materials nanoarchitectonics, with the potential to contribute to a range of medical fields, including the aforementioned example.

**Figure 17:**
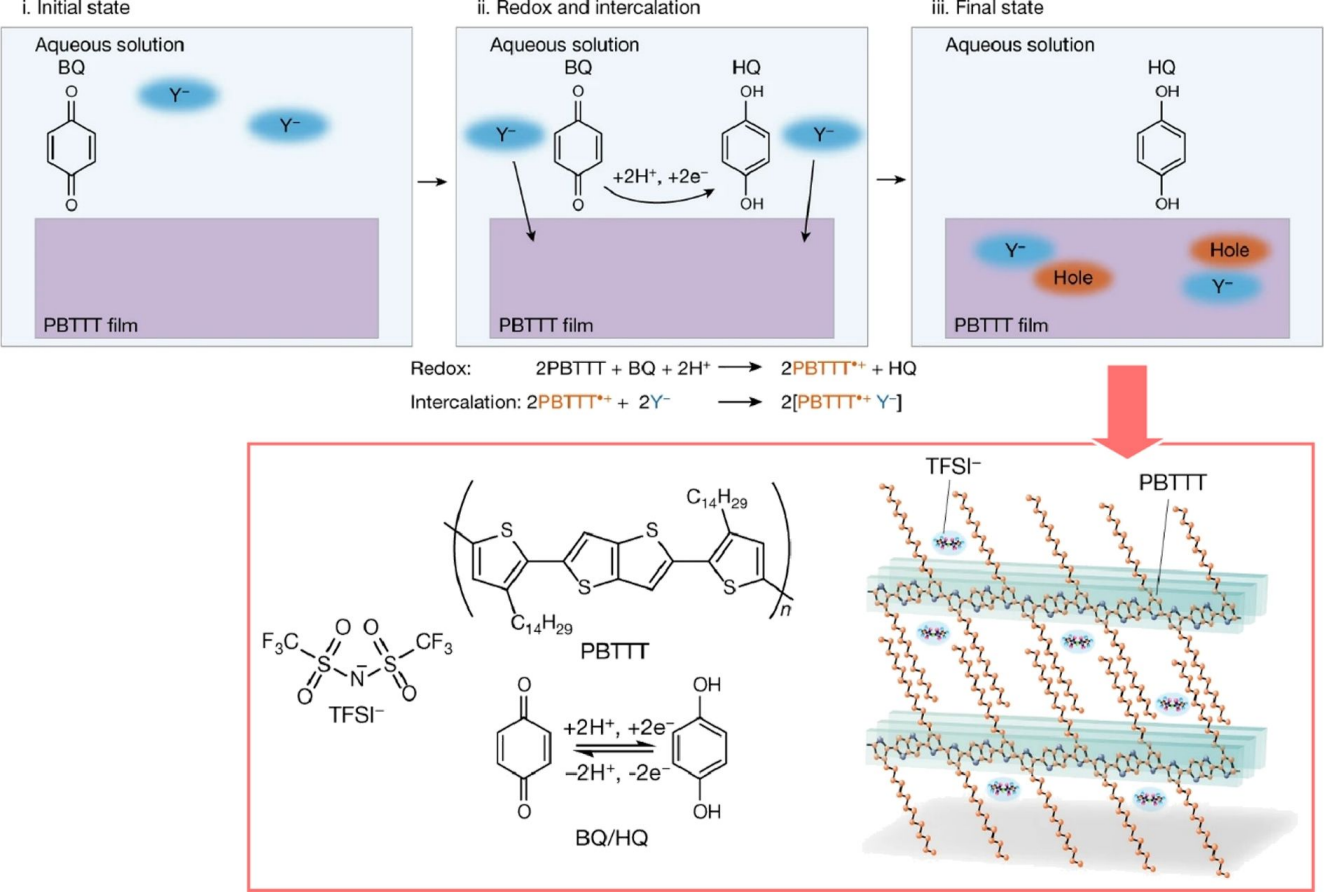
The process of chemical doping of organic semiconductors based on proton coupling electron transfer reaction and intercalation into the nanospaces formed in thin polymer films. Figure 17 is from [278] (M. Ishii et al., “Doping of molecular semiconductors through proton-coupled electron transfer“, *Nature*, vol. 622, pages 285–291, published by Springer Nature, 2023, reproduced with permission from Springer Nature). Copyright © 2023, The Author(s), under exclusive licence to Springer Nature Limited. This content is not subject to CC BY 4.0.

It is also anticipated that soft materials nanoarchitectonics using polymers will be applied in other fields, including the control of the functions of polymer semiconductors. The process of chemical doping of molecular semiconductors is based on electron transfer reactions between the semiconductors and the dopant molecules. The redox potential of the dopant is a critical factor in regulating the Fermi level of the semiconductor. The research group led by Ishii and Yamashita utilizes proton coupling electron transfer reactions, which are extensively employed in biochemical processes [278]. They make effective use of intercalation into the nanospaces formed in thin polymer films (Figure 17). Molecular redox reactions that are precisely controlled at room temperature are employed in biochemical processes, including proton coupling electron transfer reactions. For instance, two-electron transfer reactions and two-proton transfer reactions are frequently employed to establish a disparity in the electrochemical potential of protons across a membrane for the generation of cellular energy. An illustrative example of a reversible proton coupling electron transfer reaction is the conversion of benzoquinone to the hydroquinone, which occurs by the acceptance of two electrons and two protons. The compounds in use do not react with water due to their weak redox properties, rendering them stable redox agents at room temperature. The redox potential of the proton coupling electron transfer reaction between benzoquinone and hydroquinone is precisely controlled by pH, in accordance with the Nernst equation. In order to compensate for the charge carriers in the semiconductor, dopant ions must be supplied through the redox reaction. The efficient doping of polymeric organic semiconductor thin films is achieved through the synergistic reaction of the proton coupling electron transfer reaction and the insertion of hydrophobic ions. This process has enabled the efficient doping of crystalline organic semiconductor thin films at room temperature. By examining the conditions, it is possible to achieve strong chemical doping beyond the conventional limits in aqueous solutions at room temperature. The demonstrated proton coupling electron transfer reaction-based route may facilitate the fabrication of advanced and reliable organic semiconductor devices, including sensors and bioelectronics, in a more expedient manner.

The presented examples illustrate the use of polymers in soft materials nanoarchitectonics. The design of polymers is a well-established field, and chemical design can be executed with a high degree of skill. Furthermore, a range of biopolymers can be sourced from natural sources. Nevertheless, there are numerous ongoing nanoarchitectonics projects that control the assembly of polymers. Consequently, a substantial corpus of knowledge and experiences has been accumulated in the design and integration of polymer materials. By combining this knowledge, a variety of results can be obtained in soft materials nanoarchitectonics, spanning the fields of electronics and medicine.

### Gel nanoarchitectonics

Gels are representative soft materials that exhibit flexibility and softness. They are soft and flexible substances comprising polymers and molecular aggregates that are predominantly solvated and integrated with the solvent [279–281]. Furthermore, gel materials with excellent stimuli responsiveness and biocompatibility have also been widely developed. As the nanoarchitectonics of the polymers and molecular aggregates that constitute gels evolve, the design and functionality of gels are becoming increasingly diverse and sophisticated. This section presents a selection of recent examples of functional gel materials, which illustrate the potential of soft materials nanoarchitectonics in research.

The field of gel nanoarchitectonics is frequently linked to the study of biological tissue formation. The composition of living cells and organisms includes a multitude of biomolecules, which can be regulated in terms of both concentration and spatial distribution. This enables the exertion of complex biological functions. Synthetic multinetwork hydrogels can be considered analogous to extracellular matrices, and have attracted attention due to their exceptionally high toughness and other properties. In a recent review, Kubota provided an overview of the advancements in research on supramolecular-polymer composite hydrogels, a novel class of multinetwork hydrogels [282]. Composite hydrogels can be designed to integrate the stimuli responsiveness of supramolecular gels with the rigidity of polymer gels in a rational manner. Supramolecular–polymer composite hydrogels integrate the properties of supramolecular networks and polymer networks, including stimuli responsiveness and rigidity/toughness. This distinctive attribute can also be harnessed for the regulated release of protein-based biopharmaceuticals. Furthermore, it has been demonstrated that by incorporating functional molecules such as enzymes and their inhibitors, supramolecular polymer composite hydrogels can be employed as matrices for the controlled release of protein biopharmaceuticals in response to antibodies. It is anticipated that these hydrogels will prove useful in a number of biomedical applications, including the three-dimensional controlled release of drugs and proteins, the construction of hierarchical organoids, and the development of implantable and injectable gel devices. One of the principal avenues for enhancing the functionality of composite hydrogels is to emulate the intricate hierarchical and dynamic structure of biological tissues. In this context, the concept of nanoarchitectonics is of significant importance.

**Figure 18:**
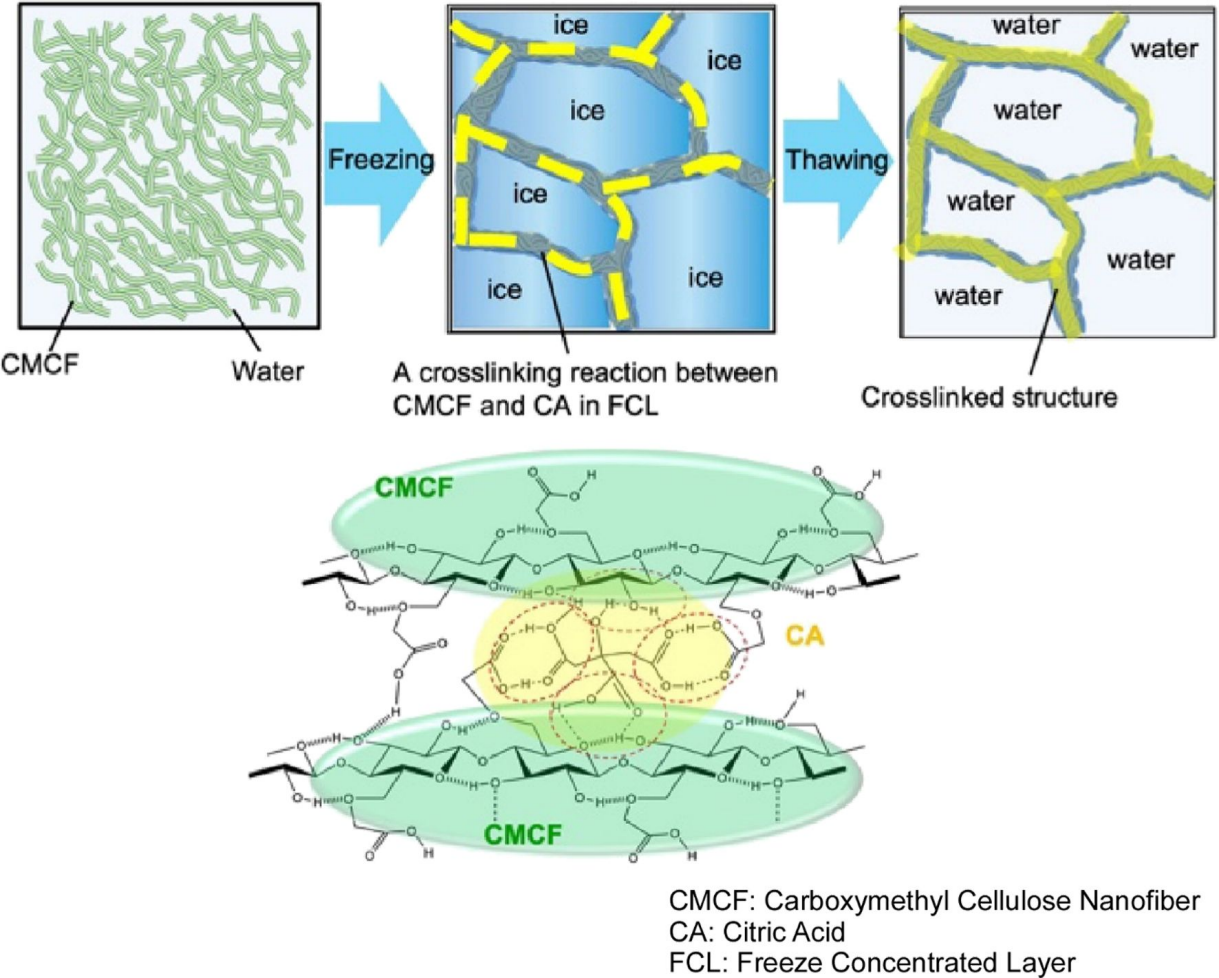
Nanoarchitectonics of carboxymethylcellulose nanofiber hydrogels involving the addition of citric acid to frozen carboxymethylcellulose nanofibers and subsequent thawing of the mixture. Figure 18 was reproduced from [283] (Y. Sekine et al., “Freeze-Concentrated Layers as a Unique Field for the Formation of Hydrogels”, *Bull. Chem. Soc. Jpn.*, 2023, vol. 96, issue 10, pages 1150–1155, https://doi.org/10.1246/bcsj.20230146); by permission of Oxford University Press on behalf of the Society. © 2023 The Chemical Society of Japan. This content is not subject to CC BY 4.0.

Hydrogels are created by nanoarchitectonics using abundant water, and the phase separation of ice crystals, solutes, and bound water that occurs during freezing can be employed as a reaction field to control the hierarchical structure of the hydrogel. In a recent review, Sekine and Nankawa presented research on carboxymethylcellulose nanofiber hydrogels formed through solid–quasi-liquid phase separation [283]. The synthesis of carboxymethylcellulose nanofiber hydrogels is a straightforward process, involving the addition of citric acid to frozen carboxymethylcellulose nanofibers and subsequent thawing of the mixture (Figure 18). Prior to the melting of the ice crystals, a rearrangement of the carboxymethylcellulose nanofiber structure via hydrogen bonding occurs within the freeze-concentrated layer. During the process of freeze concentration, carboxymethylcellulose nanofibers and bound water are retained in high concentrations. The cross-linking reaction in this distinctive environment contributes to the formation of carboxymethylcellulose nanofiber hydrogels with high mechanical strength. One of the distinctive properties of this hydrogel is that it possesses a substantial internal volume, high fluidity, and the capacity to readily absorb and release water. The oriented structure of carboxymethylcellulose nanofibres, which exhibit both hydrophobic and hydrophilic properties, contributes to the rapid dehydration and absorption of water. The fabricated hydrogels are non-toxic and biodegradable, and have a highly fluid internal structure formed from macropores, rendering them useful as adsorbents, carriers, or biomaterial usages. As demonstrated in the system presented here, there is considerable potential for the application of synthetic methods and materials utilizing freezing, and it is anticipated that this process will prove to be a valuable addition to the field of nanoarchitectonics.

**Figure 19:**
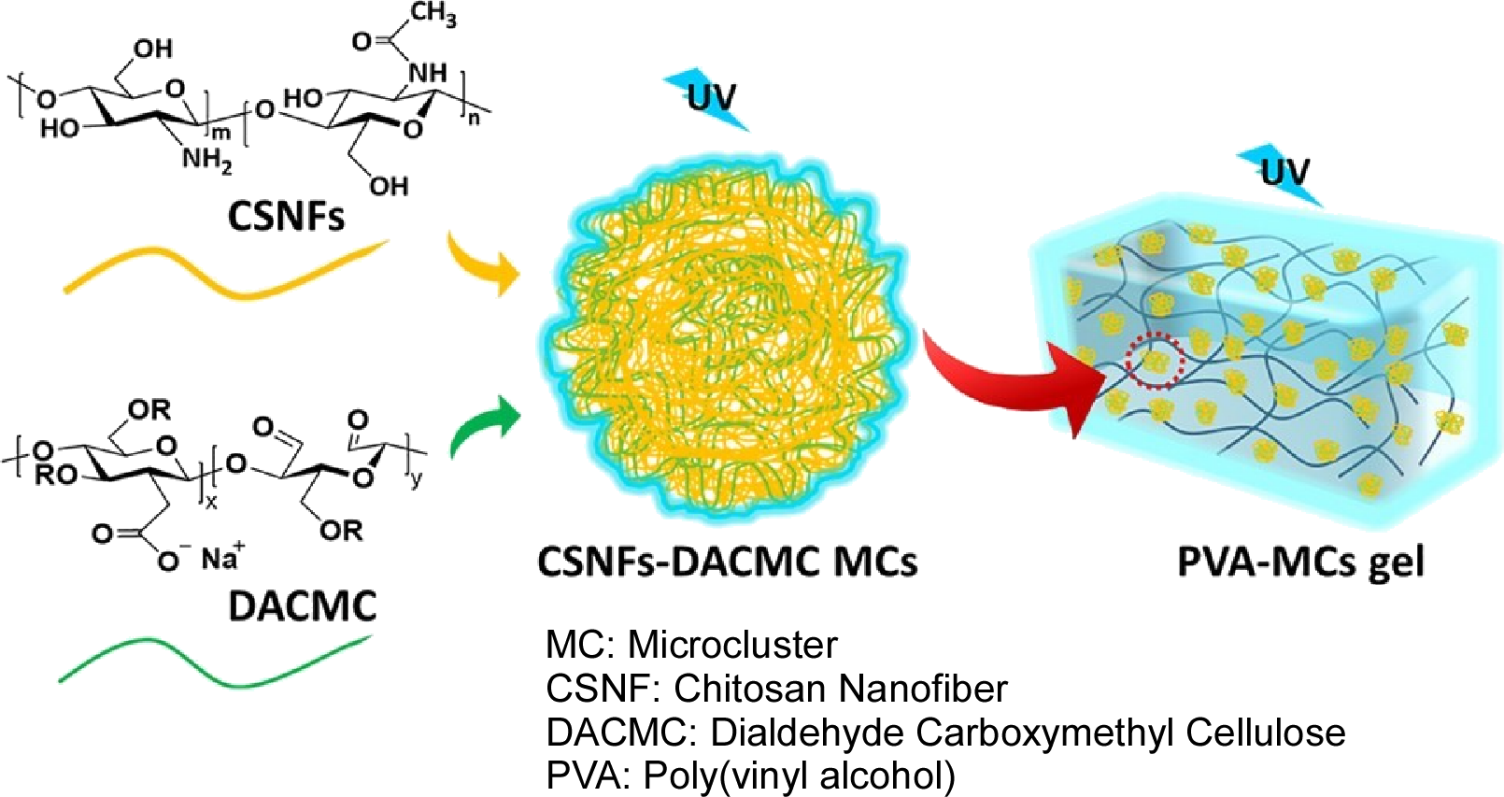
Nanoarchitectonics of co-assembled chitosan nanofibres and dialdehyde carboxymethyl cellulose with multiple cross-links to fabricate microclusters with fluoresce under ultraviolet light subsequently combined with polyvinyl alcohol and hydrogel to produce fluorescent materials. Figure 19 was reproduced from [284] (M. P. H. Pedige et al., “Clusterization-triggered emission of polysaccharide-based microclusters induced by the co-assembly of chitosan nanofibers and dialdehyde carboxymethyl cellulose”, *Bull. Chem. Soc. Jpn.*, 2024, vol. 97, issue 6, uoae065, https://doi.org/10.1093/bulcsj/uoae065); by permission of Oxford University Press on behalf of the Society. © The Author(s) 2024. Published by Oxford University Press on behalf of the Chemical Society of Japan. All rights reserved. This content is not subject to CC BY 4.0.

The potential of gel nanoarchitectonics as a light-emitting material is also being investigated. Some natural polymers have the potential to function as cluster emitters. Such materials can be combined with gels to create novel functional products. Sugawara, Uyama, and colleagues co-assembled chitosan nanofibres and dialdehyde carboxymethyl cellulose with multiple cross-links to fabricate microclusters fluorescing under ultraviolet light (Figure 19) [284]. The fabricated microclusters were subsequently combined with poly(vinyl alcohol) and hydrogel to produce fluorescent materials. The microclusters are capable of forming stable structures in aqueous environments due to the formation of cross-links through imine bonds, ionic interactions, and hydrogen bonds between the polysaccharides. The multiple interactions and heteroatom character of both chitosan nanofibres and dialdehyde carboxymethyl cellulose enabled clustering-induced emission through conjugation through space. Consequently, the hydrogel of microclusters is quenched when it interacts with specific ions. The quenching of emission permitted the detection of specific metal ions, including Cu^2+^ and Fe^3+^. The composite hydrogel exhibited excellent luminescence properties and demonstrated stable performance over an extended period of time at various temperatures. These characteristics render them promising materials for use in metal sensing and bioimaging.

**Figure 20:**
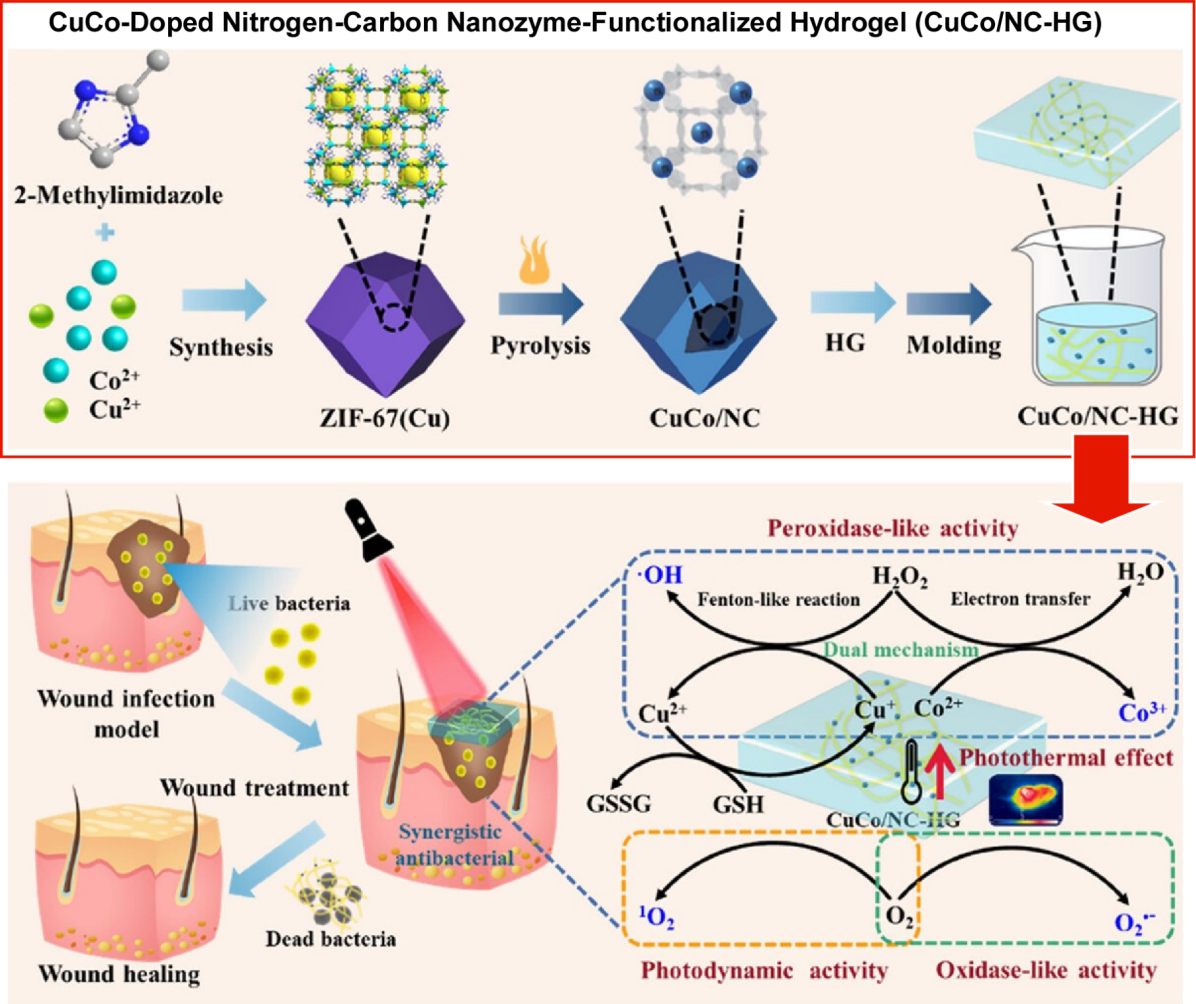
Nanoarchitectonics of bimetallic Cu-/Co-doped nitrogen–carbon nanozyme-functionalized hydrogel: bimetallic CuCo nanomaterials immobilized on the surface of the hydrogel to display photoresponsive, enhanced enzymatic effects when exposed to near-infrared irradiation (808 nm), exhibiting robust peroxidase-like and oxidase-like activities. Figure 20 was adapted with permission from [285], Copyright 2024 American Chemical Society. This content is not subject to CC BY 4.0.

The incorporation of active metal sites within the gel structure facilitates the development of functional materials for medical applications. Infection and transmission represent significant challenges in the modern medical field. It is of paramount importance to develop materials that can effectively treat bacterial infections and overcome the issue of antimicrobial resistance. Gao and colleagues reported the development of a bimetallic CuCo-doped nitrogen–carbon nanozyme-functionalized hydrogel (Figure 20) [285]. ZIF-67(Cu), a MOF, was calcined at 400 °C to prepare bimetallic CuCo nanomaterials, which were then immobilized on the surface of the hydrogel through a process of nanoarchitectonics involving the hydrothermal growth method. The resulting hydrogels display photoresponsive, enhanced enzymatic effects when exposed to near-infrared irradiation (808 nm), exhibiting robust peroxidase-like and oxidase-like activities. The peroxidase-like activity can be enhanced by a dual mechanism, comprising the direct oxidation of Co^3+^ and the generation of ^•^OH based on Cu^+^, which oxidizes glutathione. The CuCo-doped nitrogen–carbon nanozyme-functionalized hydrogel displays oxidase-like activity, enabling the generation of superoxide anion (O_2_^•–^). The photodynamic activity of the CuCo-doped nitrogen–carbon nanozyme-functionalized hydrogel, when irradiated with near-infrared light, results in the generation of singlet oxygen. Furthermore, it exhibits a high photothermal conversion effect, which not only facilitates the eradication of bacteria but also enhances the generation efficiency of ^•^OH and O_2_^•–^ and promotes the consumption of glutathione. The antibacterial efficacy of the CuCo-doped nitrogen–carbon nanozyme-functionalized hydrogel was demonstrated by its potent bactericidal action against methicillin-resistant *Staphylococcus aureus* and ampicillresistant *Escherichia coli*. The CuCo-doped nitrogen–carbon nanozyme-functionalized hydrogel has the capacity to facilitate the healing of infected wounds without the induction of inflammation. Further optimization and development may facilitate its utilization as an innovative tool in anti-infective therapy.

**Figure 21:**
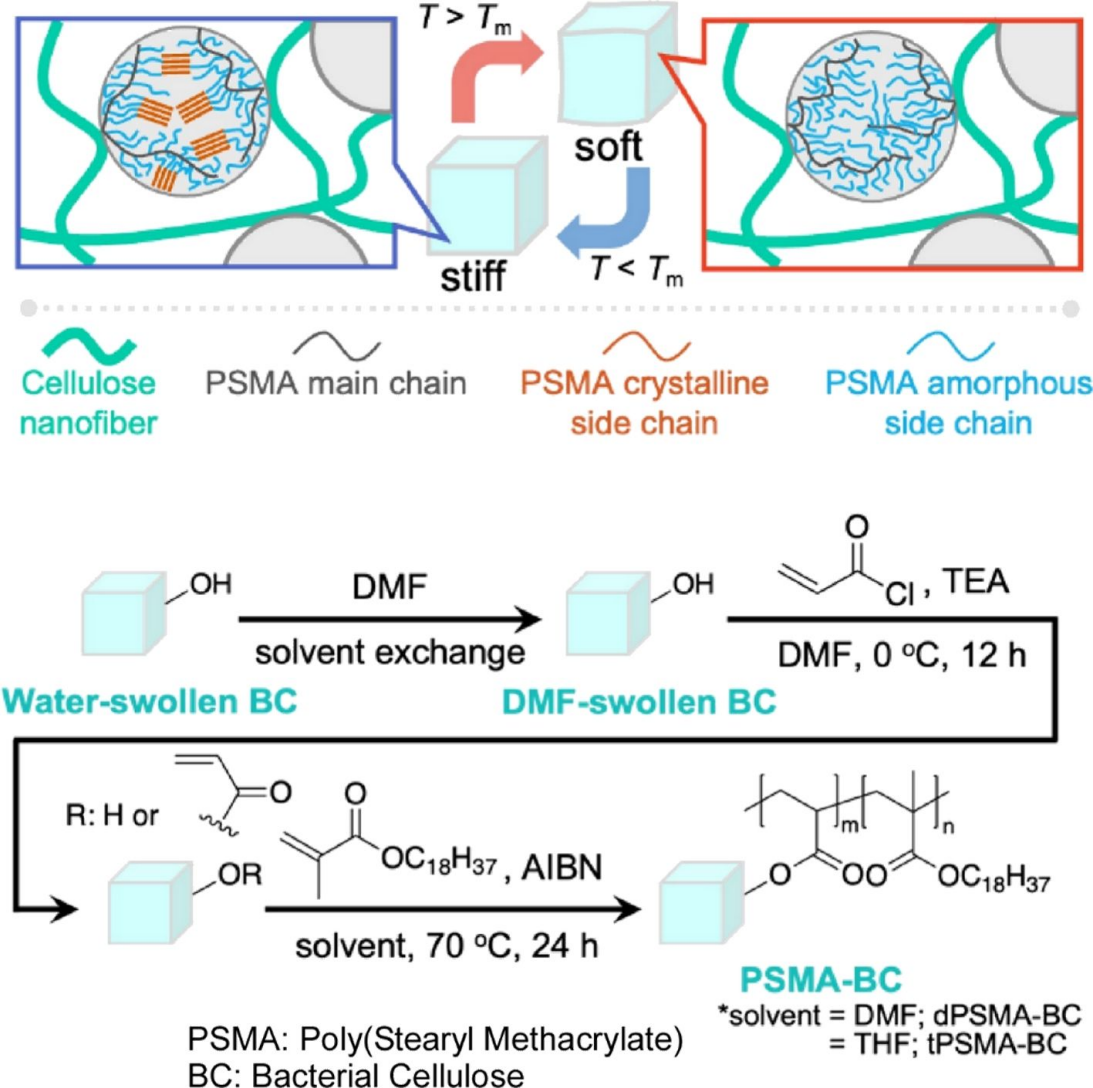
Nanoarchitectonics of thermoresponsive hydrogels with switchable mechanical properties by incorporating poly(stearyl methacrylate) as the responsive domain and bacterial cellulose as the supporting hydrogel exhibiting a distinct phase change between crystalline and molten states in response to cooling and heating due to the packed structure of the long aliphatic side chains of the comb-shaped polymer. Figure 21 was reproduced from [286] (N. Roopsung et al., “Switchable Stiffness of Composite Hydrogels Triggered by Thermoresponsive Phase-Change Particles”, *Bull. Chem. Soc. Jpn.*, 2023, vol. 96, issue 7, pages 636–638, https://doi.org/10.1246/bcsj.20230094); by permission of Oxford University Press on behalf of the Society. © 2023 The Chemical Society of Japan. This content is not subject to CC BY 4.0.

In gel nanoarchitectonics, the combination of components with disparate properties allows for the generation of complementary or competitive effects. Uyama and colleagues developed thermoresponsive hydrogels with switchable mechanical properties by incorporating poly(stearyl methacrylate) as the responsive domain and bacterial cellulose as the supporting hydrogel (Figure 21) [286]. The poly(stearyl methacrylate) particles within the bacterial cellulose display a reinforcing effect below the melting point, while above the melting point, this effect is reduced. This results in a notable degree of responsiveness. Bacterial cellulose provides a stable structural framework for the smart hydrogel. Meanwhile, the thermoresponsive domain of the material is poly(stearyl methacrylate). The poly(stearyl methacrylate) exhibits a distinct phase change between crystalline and molten states in response to cooling and heating due to the packed structure of the long aliphatic side chains of the comb-shaped polymer. This phase change results in the smart hydrogel exhibiting switchable stiffness behavior. In the composite gels, a clear decrease in Young’s modulus was observed with increasing temperature from 25 to 50 °C. Furthermore, the difference in Young’s modulus at 25 and 50 °C widened with increasing amounts of incorporated poly(stearyl methacrylate). In this study, they put forward the incorporation of poly(stearyl methacrylate) particles into the hydrogel network as a promising approach to enhance the thermoresponsiveness. It is anticipated that the nanoarchitectonics methodology will facilitate the industrial utilization of hydrogels as artificial muscles and soft robotic components.

**Figure 22:**
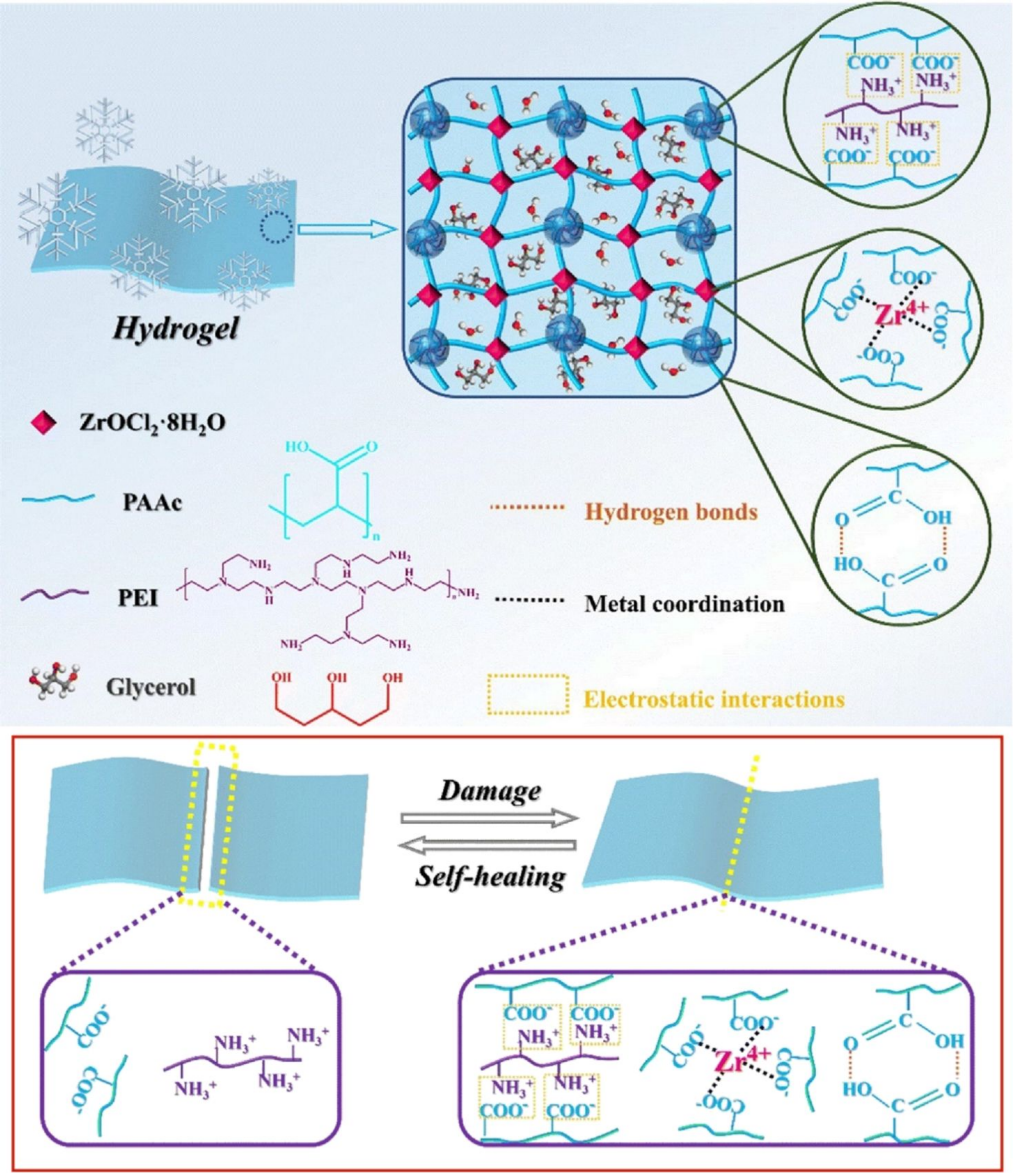
Nanoarchitectonics of composite hydrogels with high mechanical strength and rapid and efficient self-healing capabilities based on multiple synergistic effects. Figure 22 was used with permission of The Royal Society of Chemistry, from [287] (“Nanoarchitectonics composite hydrogels with high toughness, mechanical strength, and self-healing capability for electrical actuators with programmable shape memory properties” by Y. Wang et al., *Nanoscale*, vol. 15, issue 46, © 2023); permission conveyed through Copyright Clearance Center, Inc. This content is not subject to CC BY 4.0.

Hydrogel materials have the potential to be highly beneficial in a number of different fields, including flexible electronic devices, tissue engineering, and wound dressings. In order to achieve this, it is necessary to ensure that the material possesses sufficient mechanical properties, recovery performance, and self-healing speed. To address these challenges, Gao and colleagues designed composite hydrogels with high mechanical strength, and rapid and efficient self-healing capabilities based on multiple synergistic effects (Figure 22) [287], which are suitable for use in flexible electronic devices, tissue engineering, and wound dressings. The formation of the hydrogels is primarily driven by metal coordination between Zr^4+^ and carboxyl groups present in the reaction-generated polymer network. Additionally, electrostatic interactions between carboxyl groups and protonated amine groups of poly(ethyleneimine) contribute to the hydrogel formation process. The prepared hydrogels display excellent mechanical and self-healing properties, which are the result of the further intertwining of multiple synergistic interactions. The interactions between the weak polyelectrolytes, polyethyleneimine and polyacrylic acid, facilitate an improvement in the elasticity of the hydrogels, thereby imparting rapid self-healing properties. In particular, the robust metal–ligand interactions and the presence of multiple reversible interactions resulted in the hydrogel exhibiting superior mechanical properties compared to other hydrogel materials. The hydrogel also exhibits excellent self-healing ability due to the multiple reversible effects. The hydrogel demonstrated a rapid self-healing capacity and exhibited long-term stability. The formation of composite hydrogels with high toughness, high mechanical strength, and excellent self-healing ability was achieved through the utilization of multiple synergistic interactions. It is anticipated that the composite hydrogel will find utility as an actuator or robot arm in intelligent applications. Furthermore, the temperature-sensitive property enables its utilization as a shape-memory material. Furthermore, the capacity of the material to alter its shape in response to varying temperatures enables the generation of programmable effects.

**Figure 23:**
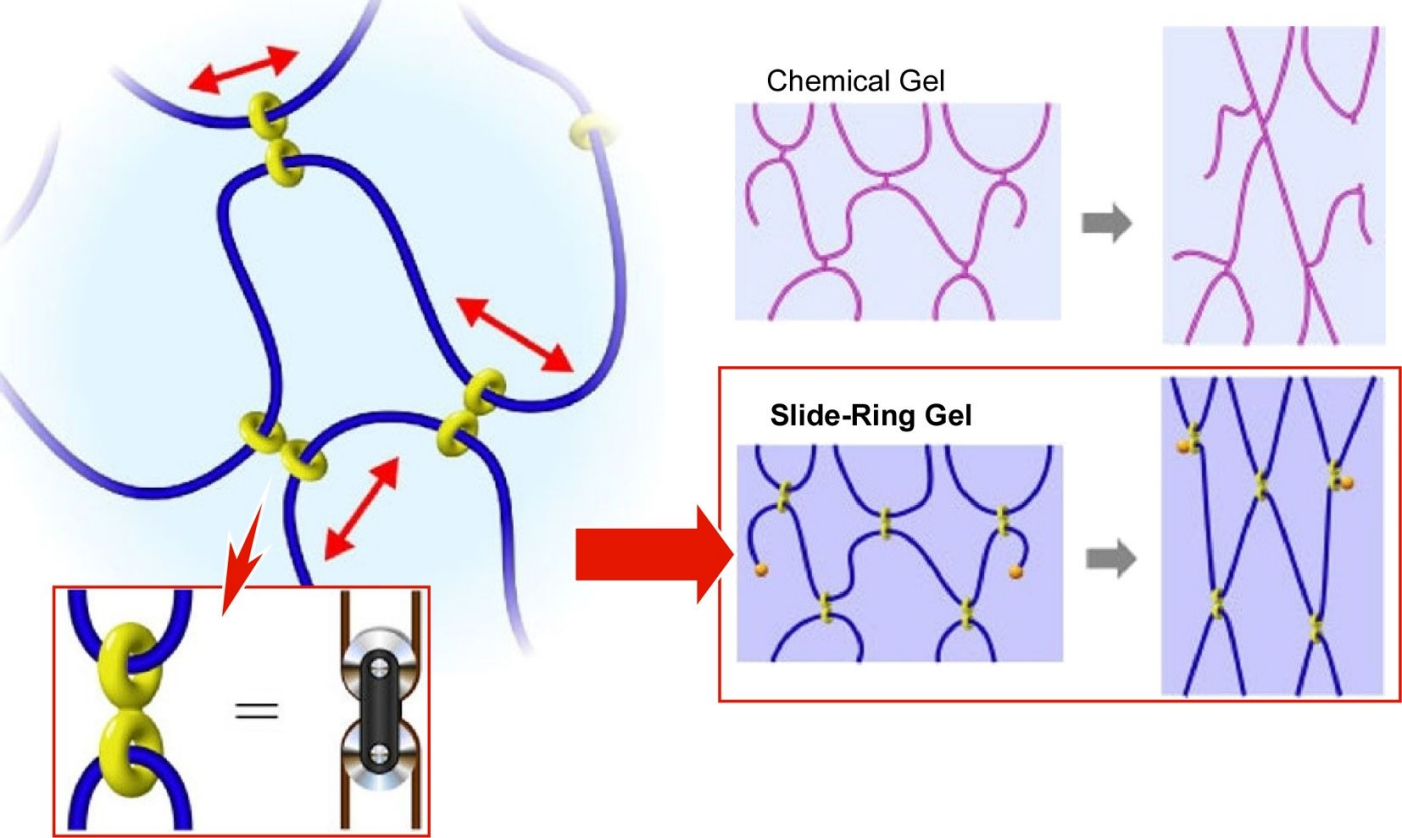
Nanoarchitectonics of the slide-ring gels employing rotaxane structures utilized in supramolecular chemistry as cross-linking points that are able to move freely along the polymer chains, thereby equalizing the tension of the polymer chains in a manner analogous to pulleys. Figure 23 is from [288] (K. Ito, “Novel Cross-Linking Concept of Polymer Network: Synthesis, Structure, and Properties of Slide-Ring Gels with Freely Movable Junctions“, *Polymer Journal*, vol. 39, pages 489–499, published by Springer Nature, 2007, reproduced with permission from SNCSC). This content is not subject to CC BY 4.0.

An intriguing gel design from the perspective of nanoarchitectonics is the slide-ring gels developed by Ito and colleagues. This employs rotaxane structures utilized in supramolecular chemistry as cross-linking points [288]. The utilization of this supramolecular structure with topological properties gives rise to a novel type of gel that is distinct from both physical and chemical gels. In this gel, the polymer chains with bulky end groups are not covalently cross-linked in the manner of chemical gels, nor do they interact attractively as in physical gels. In contrast, the cross-linking structure is formed by the polymer chains passing through each of the figure-of-eight pore structures (Figure 23). Consequently, these cross-links are able to move freely along the polymer chains, thereby equalizing the tension of the polymer chains in a manner analogous to pulleys. This phenomenon is referred to as the pulley effect. In chemical gels, the polymer chains are gradually broken due to the unequal distribution of polymer length between the fixed cross-links. Conversely, in slide-ring gels, the polymer chains are capable of passing through the figure-eight cross-links that serve as pulleys. Furthermore, the equalization of tension can occur not only within a single polymer chain, but also between adjacent polymers that are connected by figure-eight cross-links. Consequently, the slide-ring gels display remarkable flexibility and capacity for volume change. The mechanical behavior of the slide-ring material can be adequately explained by a free-junction model that takes into account the pulley effect. In contrast to conventional cross-linked polymer materials, no elastic instability is observed. The mechanical behaviors of the slide-ring material were found to be analogous to those observed in biological materials, including mammalian skin, blood vessels, and tissues. It is anticipated that this novel gel material will have applications not only in gel-like substances such as soft contact lenses and polymer batteries, but also in solvent-free polymer materials such as paints, fibres and films.

Hydrogels are typically characterized by low mechanical strength due to the limited water content of their polymeric matrix. In many cases, a sacrificial structure capable of dissipating input energy is introduced to create a tough hydrogel. However, the toughness of these gels frequently diminishes considerably when subjected to continuous cyclic loading, due to the inability of the sacrificial damage to be rapidly repaired. To overcome this fundamental limitation and obtain a tough gel, Mayumi, Ito, and colleagues developed a poly(ethylene glycol) hydrogel cross-linked with a moderate amount of polymer that forms a sliding ring [289]. The fundamental design is the slide-ring hydrogel, in which the poly(ethylene glycol) chains are connected by slidable cross-links, consisting of hydroxypropyl-α-cyclodextrin rings. The cross-links in the slide-ring gel facilitate the sliding of the poly(ethylene glycol) chains, thereby releasing the stress within the network. When subjected to uniaxial stretching, the polymer strands situated between the cross-linking points elongate and undergo uniform tensile deformation in the direction of the applied force. The unexposed highly oriented poly(ethylene glycol) chains repeatedly form and dissolve a close-packed structure, accompanied by stretching and releasing. This results in rapid and reversible strain-induced crystallization, which markedly enhances the toughness of the hydrogel. The reinforcement of the intact gel structure of the hydrogel is achieved through the utilization of strain-induced crystallization. The high toughness of the slide-ring gel can be attributed to a well-known toughening mechanism observed in rubber. The reversible formation and destruction of poly(ethylene glycol) crystals under cycles of loading and unloading is the origin of the high mechanical reversibility of the slide-ring gel.

Gels are soft materials, comprising polymers and molecular aggregates that are predominantly solvated and integrated together. As illustrated by the aforementioned examples, gel nanoarchitectonics enables the fabrication of gels with diverse properties contingent on the design, fabrication methodology, and constituents employed. The combination of components with disparate properties can yield either complementary or competitive effects. As soft materials, gels exhibit a wide range of mechanical properties and a high degree of tolerance for the incorporation of diverse substances. It is therefore anticipated that a diverse range of practical materials, including those for biomedical applications, will be developed.

### Biomaterials nanoarchitectonics

In the soft materials nanoarchitectonics, the utilization of biological materials and the advancement of biologically pertinent functions are subjects of debate. This is related to the favorable compatibility between soft materials and biological systems, as well as the soft signal responsiveness required for biological functions. Biomolecules, biomaterials and even their assemblies can be considered to fall within the category of soft materials. It is therefore highly significant to consider the nanoarchitectonics of soft materials in relation to the properties and functions of biomaterials. The following section will present some case studies illustrating the application of soft materials nanoarchitectonics in the context of biomaterials.

The initial parts of this section begins with a series of conceptual reviews. In considering the field of biomaterials nanoarchitectonics, it is essential to take into account the role of water. In particular, polymeric biomaterials are frequently utilized in aqueous environments, whereby the water molecules exhibit a range of mobilities, including non-freezing, intermediate, and free water [290–291]. It is evident that DNA and protein molecules exert an influence on the biological reactions that occur between biomaterials and biological fluids. To further the medical field, it is a significant challenge to regulate the state of hydration water through the nanoarchitectonics of polymeric chemical structures. In a recent review, Nishimura and Tanaka emphasized the significance of intermediate water [292]. In this review, the authors elucidate the synthesis, analysis, and application of polymeric biomaterials based on the concept of intermediate water. The hydration state of biomaterials affects their biocompatibility and functionality. The hydration state of biomaterials can be classified into three categories, namely, non-freezing water/tightly bound water, intermediate water/loosely bound water, and free water/scarcely bound water. The effect of intermediate water on biocompatibility is significant, as it also affects protein adsorption and cell adhesion. It can therefore be concluded that the intermediate water concept has significant potential for application in a wide range of biomedical fields. The intermediate water concept brings several benefits to a range of sectors, such as biocompatibility, non-fouling behavior, selective protein adsorption, cell attachment, tissue engineering, drug delivery systems, and the creation of multifunctional smart biomaterials for flexible and stretchable electronic devices. The intermediate water concept has also been utilized in the creation of inorganic biomaterials. The application of the intermediate water concept has expanded to cover not just organic biomaterials, but also inorganic biomaterials. The use of intermediate water’s unique characteristics offers the possibility of improving the performance and functionality of biomaterials in a wide range of biomedical applications.

**Figure 24:**
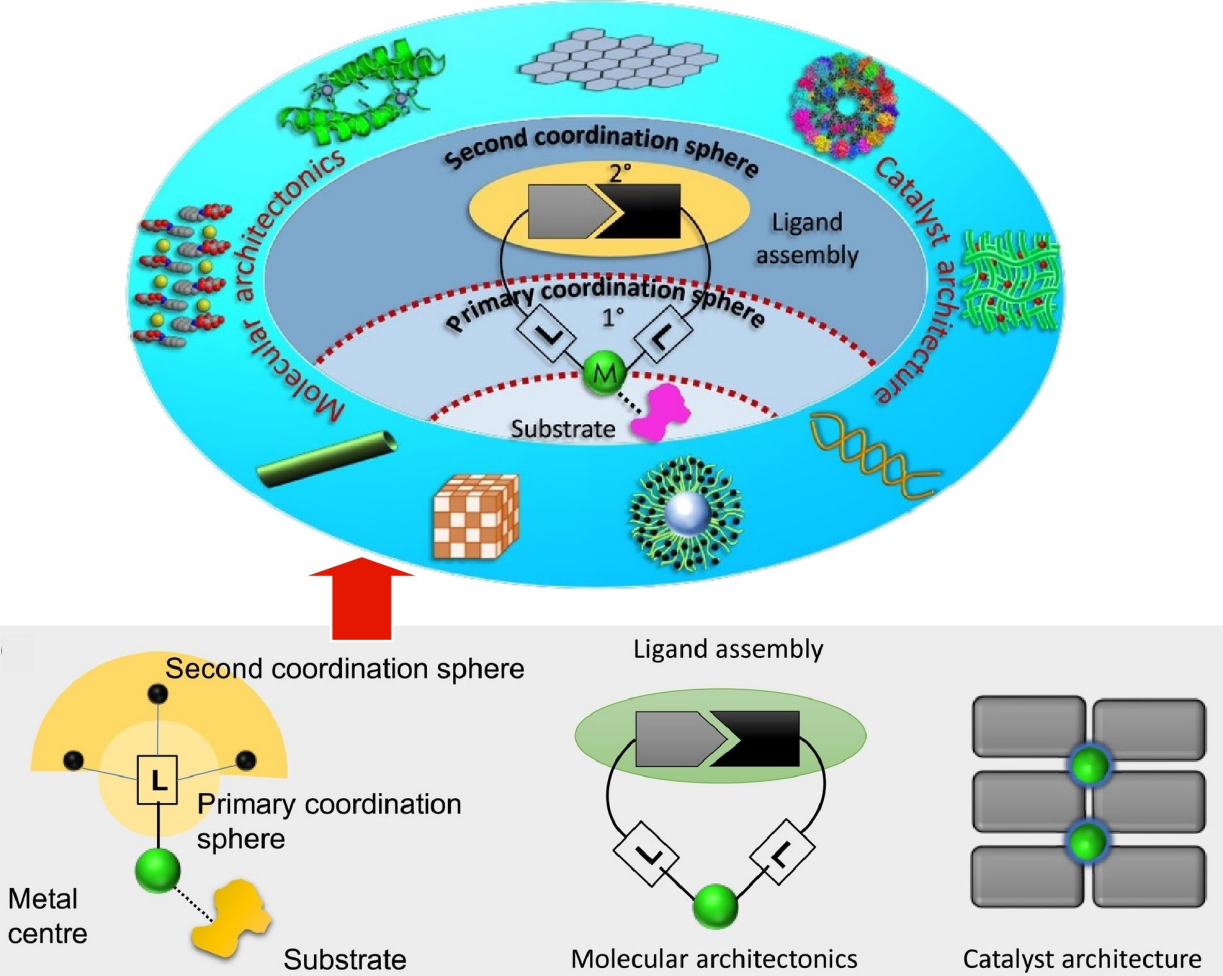
Design of catalytic architectures within the framework of molecular architectures and second coordination spheres, which have been the subject of study in the field of enzyme catalysis and various model organic reactions. Figure 24 is reproduced from [293] (B. Roy et al., “Enzyme-mimetic catalyst architectures: the role of second coordination sphere in catalytic activity”, *Bull. Chem. Soc. Jpn.*, 2024, vol. 97, issue 1, bcsj.20230224, https://doi.org/10.1093/bulcsj/bcsj.20230224); by permission of Oxford University Press on behalf of the Society. © The Author(s) 2023. Published by Oxford University Press on behalf of Chemical Society of Japan. All rights reserved. This content is not subject to CC BY 4.0.

Enzymes with well-defined three-dimensional structures incorporate information about the molecular organization in the vicinity of the active site. Molecular assemblies that are regulated by molecular architecture schemes have the potential to create enzyme-mimetic catalytic architectures. In a recent review, Roy and Govindaraju provide an overview of the state-of-the-art biomimetic catalytic architectures derived from small molecules, sugars, nucleic acids, peptides, and proteins [293]. In particular, they describe the design of catalytic architectures within the framework of molecular architectures and second coordination spheres, which have been the subject of study in the field of enzyme catalysis and various model organic reactions (Figure 24). This figure also illustrates higher-order molecular assembly architectures, including micelles, fibers, tubes, helices, nanoparticles, honeycombs, and 2D sheet structures. Additionally, it depicts metal–ligand interactions, the formation of primary and secondary coordination spheres, the binding of substrates to metal centers, and the formation of catalytic architectures. Second coordination sphere interactions have been identified as a controlling factor for a number of processes occurring during catalytic events, including substrate binding and orientation, electron transfer, bond cleavage or formation, transition state stability, and other related events. The current discussion on the role of the second coordination sphere has significant potential for future developments in the field of catalysis in fundamental biological research. Moreover, these components have the potential to be harnessed for applications in nanotechnology and nanoarchitectonics.

**Figure 25:**
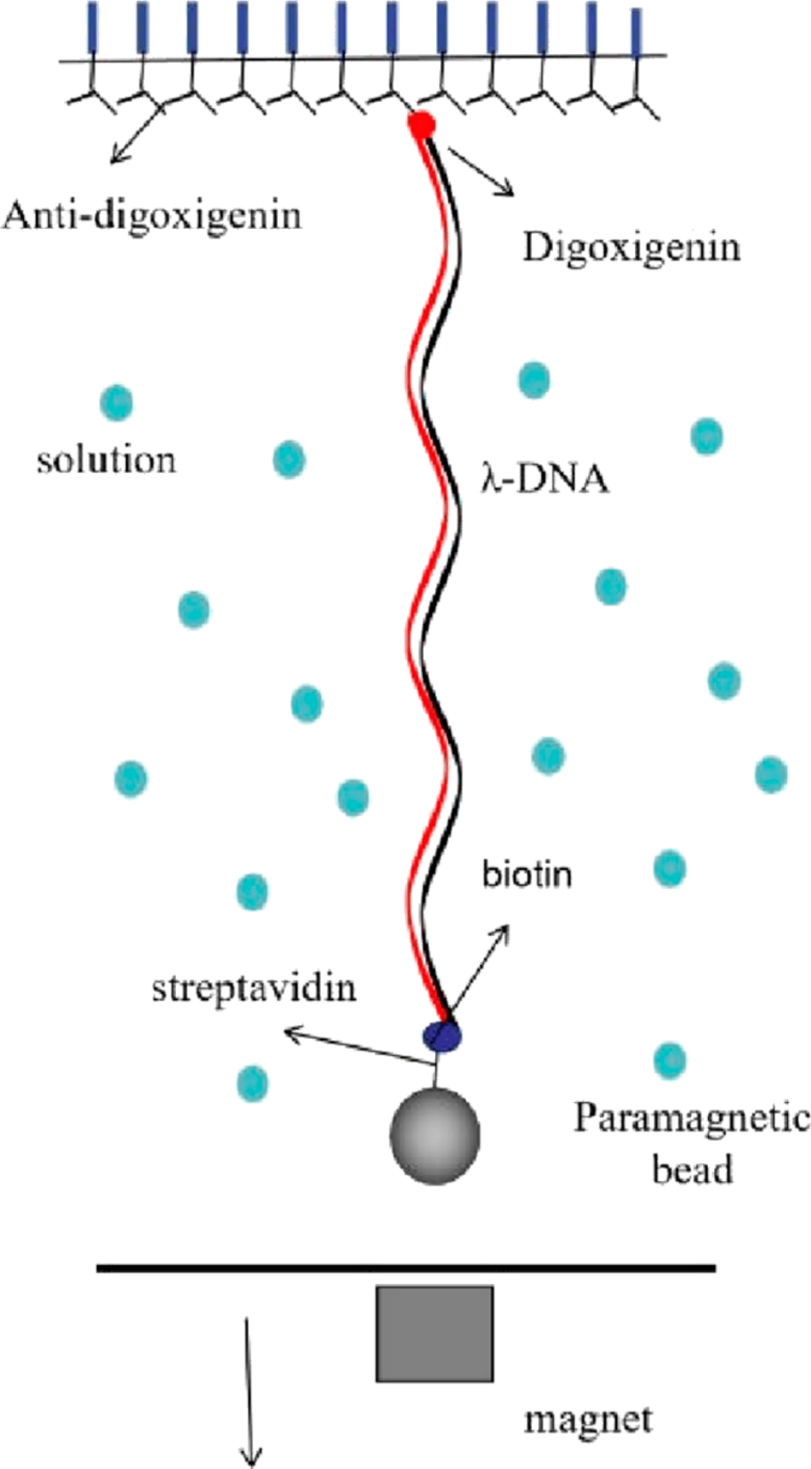
DNA–lysozyme nanoarchitectonics employing magnetic tweezers to conduct a comprehensive investigation of the complexes formed by λ-DNA and lysozyme in dilute aqueous solutions. Figure 25 was reproduced from [294] (© 2022 R. Zhang et al., published by MDPI, distributed under the terms of the Creative Commons Attribution 4.0 International License, https://creativecommons.org/licenses/by/4.0).

It is crucial to understand the properties of these biomaterials for various applications. Investigating their mechanical properties through physicochemical methods is a fundamental aspect of nanoarchitectonics. Zhang et al. employed magnetic tweezers, dynamic light scattering (DLS), and atomic force microscopy (AFM) to conduct a comprehensive investigation of the complexes formed by λ-DNA and lysozyme in dilute aqueous solutions (Figure 25) [294]. The electrostatic and hydrophobic interactions between the lysozyme units play a pivotal role in promoting phase separation in the DNA–lysozyme system. The morphology of the formed aggregates is controlled by direct interactions between protein units and electrostatic attraction between DNA and lysozyme. The DNA–lysozyme complexes were observed to undergo a transformation from loosely stretched chains to compact spheres and subsequently to less compact flower-like structures, a process that was found to be contingent upon alterations in the attached lysozyme particles. The mechanical properties were estimated. One end of a DNA molecule was affixed to a glass sidewall, while the other end was attached to a paramagnetic bead. A permanent magnet, operated by a micromanipulator system, was employed to exert a force on the DNA molecule by drawing the tethered DNA strand into proximity with the paramagnetic bead. The movement of the paramagnetic body was recorded in real time using a charge-coupled device camera, and the video of the small magnetic sphere was subsequently analyzed using software. Furthermore, the research team discovered that lysozyme causes a reversal of the charge on DNA. The neutralization of charge is a crucial step in the compaction of DNA by lysozyme, which enables the overcoming of the electrostatic repulsion between the segments of the DNA strand. As a consequence of charge neutralization and overcompensation, the morphology of the DNA undergoes a transformation, progressing from a loosely distributed state to a compact rod or sphere, and subsequently to a flower-like structure comprising a compact core. The particle size of the DNA complex, as determined by DLS, exhibited a gradual increase due to the charge reversal. Such an analysis will be of great importance for the field of nanoarchitectonics, which concerns the precise assembly of structures from biomaterials.

**Figure 26:**
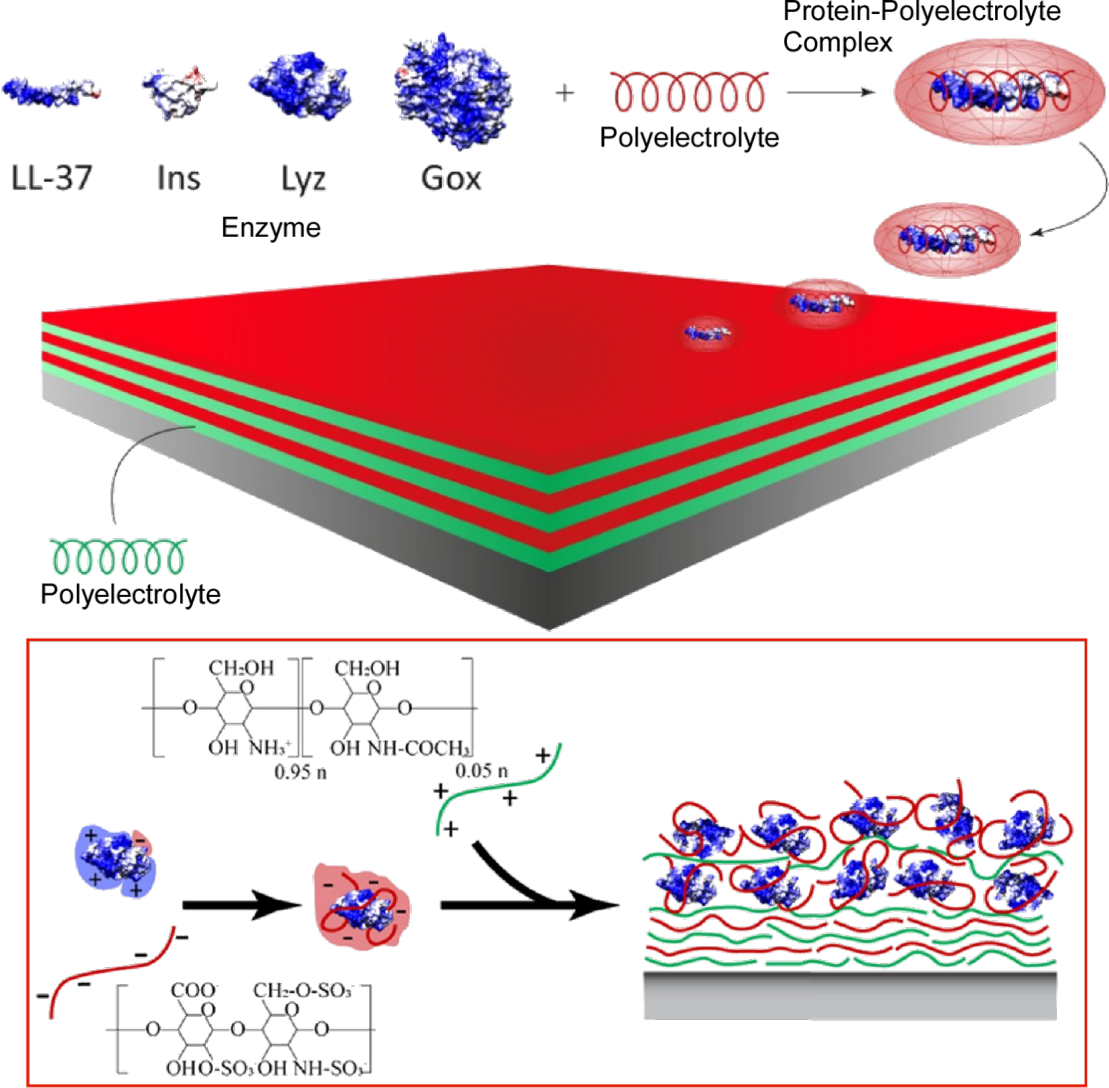
Layer-by-layer nanoarchitectonics using protein–polyelectrolyte complexes, which enables the construction of multilayers combining different proteins, thereby facilitating the formation of multifunctional films. Figure 26 was adapted with permission from [299], Copyright 2022 American Chemical Society. This content is not subject to CC BY 4.0.

A promising approach for the nanoarchitectonics of biomolecules is LbL assembly [295–297]. In addition to conventional LbL techniques, the layering of proteins with oppositely charged polyelectrolytes, which are precomplexed with polyelectrolytes, has been proposed since the 1990s [298]. In a recent study, Vranckx, Dupont-Gillain, and colleagues presented an approach for incorporating lysozyme into multilayers using protein–polyelectrolyte complexes [299]. This method (Figure 26) represents a significant advancement in the field of LbL nanoarchitectonics. This study offers additional proof of the durability and adaptability of protein–polyelectrolyte complexes as fundamental components. The LL-37 peptide, insulin, lysozyme, and glucose oxidase were encapsulated with alginate, poly(styrene sulfonate), heparin, and poly(allylamine hydrochloride). The resulting protein–polyelectrolyte complexes were subsequently assembled through a layer-by-layer process with the aid of chitosan, poly(allylamine hydrochloride), and heparin. The formation of protein–polyelectrolyte complexes through LbL assembly has made it possible to build multilayers consisting of various proteins, which aids in the creation of films with multiple functions. This technique is of particular importance in the field of nanoarchitectonics, as it allows for the creation of interfaces with proteins and peptides that are otherwise difficult to immobilize. Consequently, their functionality can be potentially modified, rendering them a promising class of building blocks for a wide range of applications. The formation of protein–polyelectrolyte complexes may prove to be a highly beneficial approach for the development of bioactive interfaces, as they offer the potential to provide higher protein and/or hydration levels than those achievable with naked proteins.

Biomolecules are highly functional and can be utilized for a variety of purposes through the application of nanoarchitectonics [300]. For instance, a variety of isothermal amplification strategies for DNA and RNA have been devised for the purpose of enhancing the efficiency of biomolecule detection in electrochemical biosensors. In a review article, Liu, Wang, Yang, and colleagues provide an overview of the strategies for signal-amplifying sensing systems, their biological applications, current challenges, and prospects in this promising new field [301]. In particular, they provide a concise overview of soft nanoarchitectonics strategies with regard to amplified electrochemical/electrochemiluminescence biosensors. From a practical standpoint, the development of a controllable electrode surface capable of preventing non-specific protein adsorption is essential for the advancement of reliable point-of-care sensing technologies. It would be beneficial to consider one-step coating strategies for bioactive sensing interfaces. It would be advantageous to develop structured systems capable of achieving efficient dual or multimode signal responses through the utilization of novel electrochemiluminescence emitters or signal response mechanisms. A more systematic approach to nanoarchitectonics will lead to improvements in electrochemical/electrochemiluminescence biosensors. Furthermore, autonomous nanoscale systems have a multitude of applications, spanning from biomedical to environmental remediation. Furthermore, the development of 2D soft nanoarchitectonics-based nanomotors that enable “chemistry on the fly” is envisaged for a diverse range of applications, including biosensing, imaging, and drug delivery.

**Figure 27:**
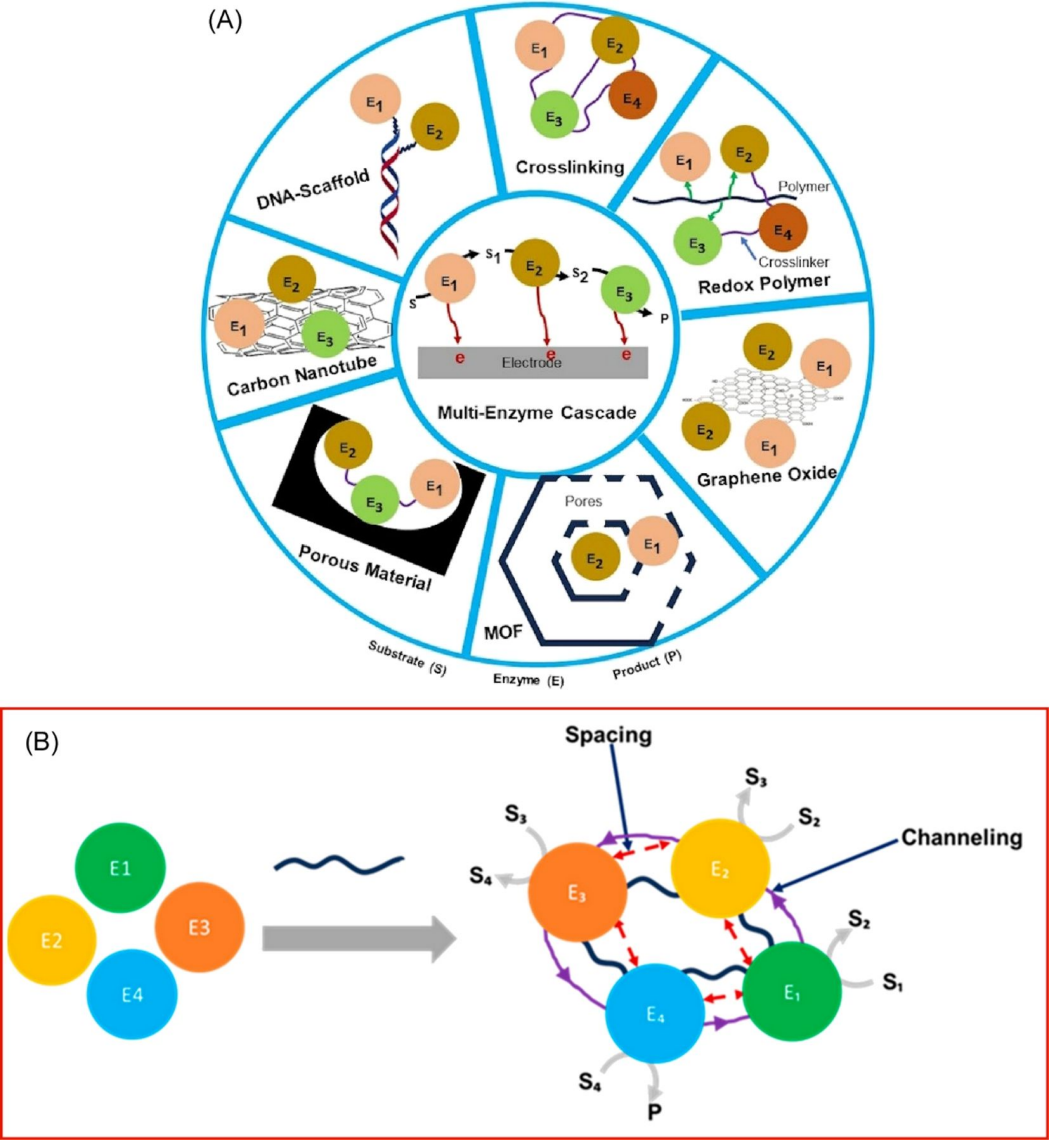
Co-immobilization of enzymes to create efficient cascade systems for the implementation of biosensors and biofuel cells: (A) overview of recent developments in approaches to facilitate the functioning of two or more enzymes in cooperation with each other; (B) two fundamental nanoarchitectonics principles of spacing and channellingfor effective routing of substrates between enzymes. Figures 27A and 27B were adapted from [305] (© 2023 S. d. Kalyana Sundaram et al., published by MDPI, distributed under the terms of the Creative Commons Attribution 4.0 International License, https://creativecommons.org/licenses/by/4.0).

The functional cooperation between biomaterials provides a clear illustration of the impact of biomaterials nanoarchitectonics. This is particularly crucial in the development of sophisticated sensor systems utilizing enzyme cascades [302–304]. The use of nanoarchitectonics with a variety of nanomaterials has the potential to enhance enzyme activity through substrate channeling, while also improving enzyme stability and reusability. Nevertheless, further improvements could be made in terms of the orientation and mutual arrangement of multiple enzymes. In order to create functional in vitro multienzyme systems, inspiration has been taken from natural multienzyme processes observed in living organisms. The nanoarchitectonics of multistep reactions of various enzymes has attracted considerable attention, particularly in the context of applications such as biosensors and the development of biofuel cells. In a recent review, Tsujimura and colleagues provide an overview of recent developments in approaches to facilitate the functioning of two or more enzymes in cooperation with each other [305]. While nanoarchitectonics with programmed DNA scaffolds has also been explored for the design of enzyme cascades, it is more straightforward and practical to design such systems through relatively random immobilization techniques, including adsorption, covalent bonding, and cross-linking. It is recommended to take into account substrate channeling when designing multienzyme cascades, with the aim of creating effective substrate oxidation pathways. This can be achieved through the co-immobilization of enzymes (Figure 27). In this instance, the substrate can be coupled to specific enzymes by regulating the length of the cross-linker spacer arms. It is of paramount importance to develop optimal protocols that take into account the length and reactivity of the cross-linkers in order to create efficient cascade systems for the implementation of biosensors and biofuel cells. Two fundamental principles that are ubiquitous across numerous systems are the precise spatial organization of enzymes and the effective routing of substrates between enzymes. These elements are of great importance in ensuring the seamless transfer of substrates between enzymes, the minimization of undesired side reactions and, ultimately, the enhancement of overall catalytic efficiency.

**Figure 28:**
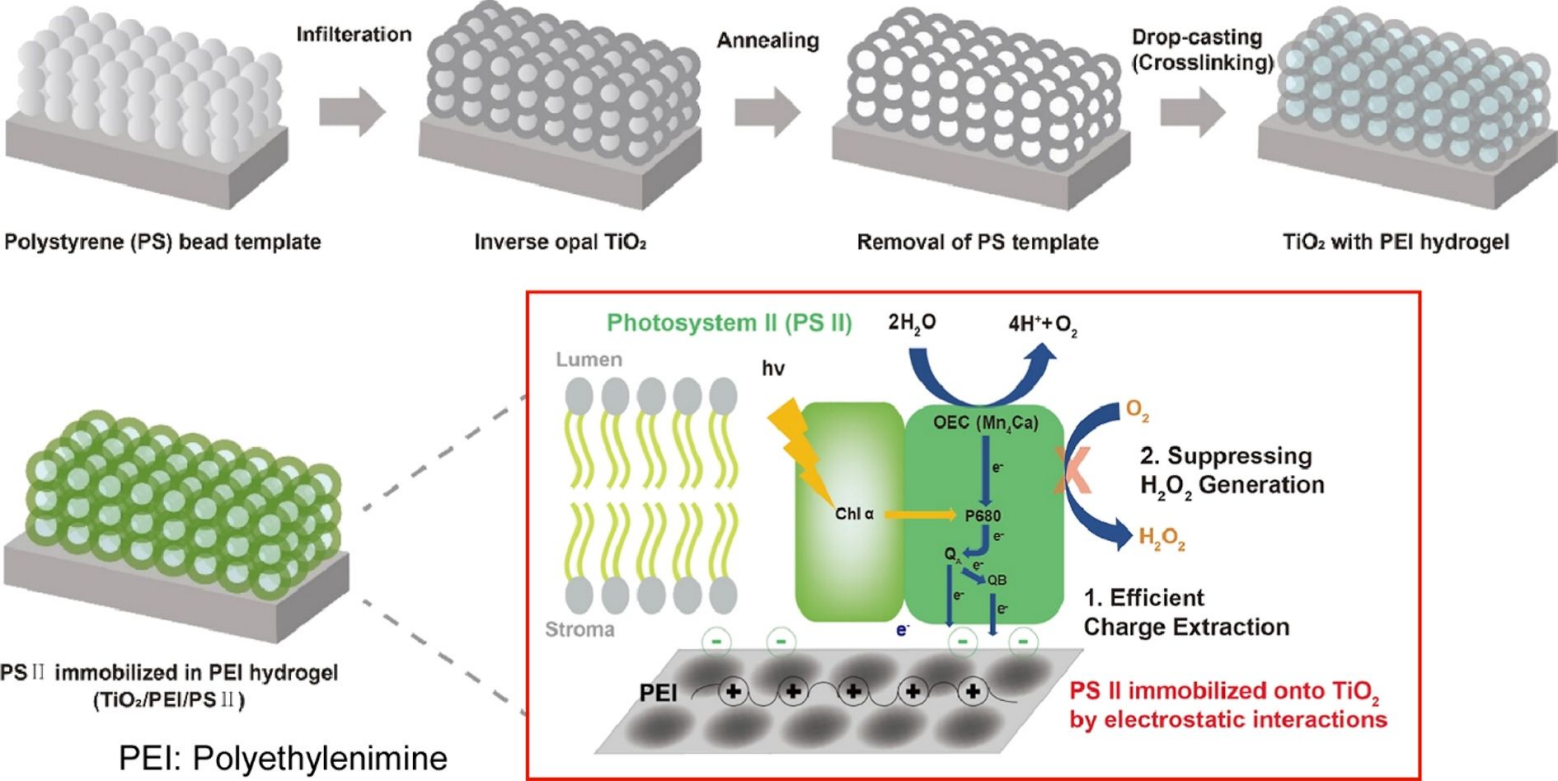
Nanoarchitectonics for the immobilization of natural photosystems on inverse opal TiO₂ using amine-rich polyethylenimine hydrogels, thereby creating organic/inorganic hybrid photoanodes. Figure 28 was adapted with permission from [306], Copyright 2024 American Chemical Society. This content is not subject to CC BY 4.0.

The process of sunlight-induced water oxidation represents a pivotal step in light-driven reductive synthesis. Despite the considerable progress made in the development of light-harvesting materials and cocatalysts, the attainment of high efficiency and stability remains a significant challenge. While various nanomaterials have been employed to achieve this function, a more efficacious approach is to organize natural photosystems. Ryu and colleagues presented a method for the immobilization of natural photosystems on inverse opal TiO_2_ using amine-rich polyethylenimine hydrogels, thereby creating organic/inorganic hybrid photoanodes (Figure 28) [306]. The effective immobilization of the large photosystem II complexes is achieved through the physical entrapment within the porous hydrophilic hydrogel, coupled with the utilization of electrostatic interactions. In particular, the properties of the polyethylenimine hydrogel, including high porosity, hydrophilicity, positive charge, and structural flexibility, facilitate the stable and uniform immobilization of photosystem II within the numerous pores of inverse opal TiO_2_ through the formation of a stable complex. The immobilization of photosystem II can be achieved through the utilization of electrostatic interactions between the positively charged amine groups of polyethyleneimine and the negatively charged stromal side of photosystem II, which allows for the specific orientation of the latter within a defined structure. This particular immobilization method ensures the efficient separation of photogenerated charges and suppresses the occurrence of undesired side reactions, such as the generation of reactive oxygen species. Consequently, photoanodes immobilized with photosystem II exhibit enhanced activity and stability with regard to solar-induced water oxidation. This strategy offers a more straightforward and practical solution to overcome challenges such as controlling the orientation of photosystem II, suppressing side reactions, and relying on complex processes for immobilizing photosystem II. This suggests the potential benefits of hybrid nanoarchitectonics, which can utilize the distinctive structural characteristics of biomaterials.

**Figure 29:**
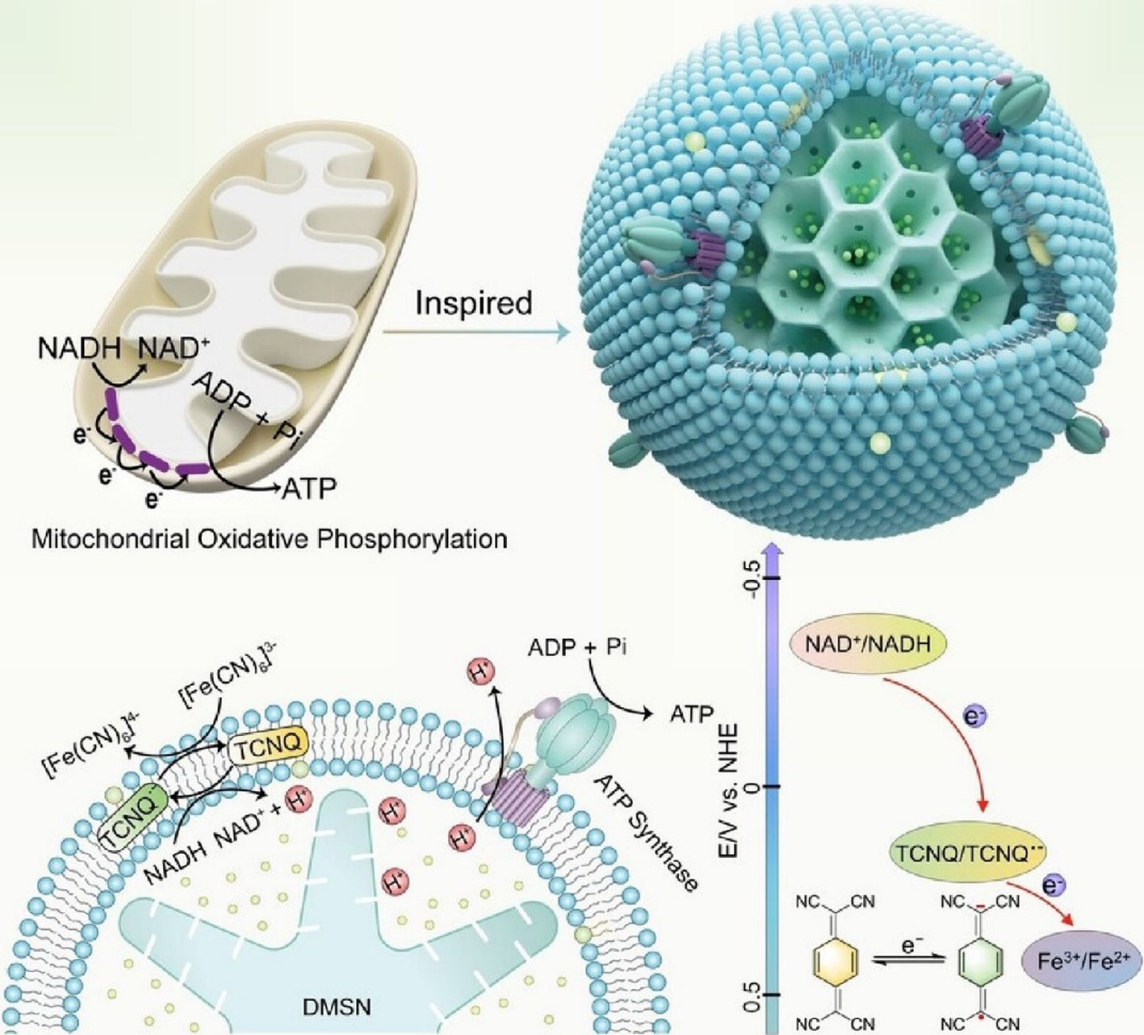
Nanoarchitectonics of a synthetic electron shuttle that activates a mitochondrial-like transmembrane chemoenzyme cascade reaction with dendritic mesoporous silica microparticles as the inner compartment and ATP synthase-reconstituted proteoliposomes as the outer compartment. Figure 29 was reproduced from [307], T. Wang et al., “Nanoarchitectonics with a Membrane-Embedded Electron Shuttle Mimics the Bioenergy Anabolism of Mitochondria”, *Angew. Chem. Int. Ed.*, with permission from John Wiley and Sons. Copyright © 2024 Wiley-VCH GmbH. This content is not subject to CC BY 4.0.

Additionally, there are endeavors to articulate sophisticated functions analogous to those observed in living organisms through the meticulous integration of biomaterials with enhanced capabilities. For instance, the utilization of bioenergy through transmembrane redox reactions in artificial systems represents a challenging research topic. Fei, Li and colleagues organized a synthetic electron shuttle that activates a mitochondrial-like transmembrane chemoenzyme cascade reaction (Figure 29) [307]. The synthetic electron shuttle activates a transmembrane chemoenzyme cascade reaction in a mitochondrial-like nanostructure, thereby facilitating enhanced bioenergy assimilation. The nanoarchitectonics employs dendritic mesoporous silica microparticles as the inner compartment and ATP synthase-reconstituted proteoliposomes as the outer compartment. The small synthetic electron shuttle embedded in the lipid bilayer facilitates a transmembrane redox reaction to convert NADH to NAD^+^ and protons in a manner analogous to that observed in natural enzymes of the mitochondrial respiratory chain. The conversion of NADH to NAD^+^, which releases protons to generate an outward transmembrane proton gradient, drives ATP synthase and thus ATP synthesis. This results in an enhanced outward proton gradient and the activation of ATP synthase, thereby improving the efficiency of catalytic ATP synthesis. The distinctive structural effect is that the hierarchical nanostructure and porosity distribution of the internal compartments significantly enhance the assimilation of bioenergy. The results presented here represent a novel and efficient approach to the realization of artificial bioenergy production, with considerable potential for the development of ATP-driven bioapplications. These findings open up a new avenue for the realization of enhanced bioenergy assimilation.

**Figure 30:**
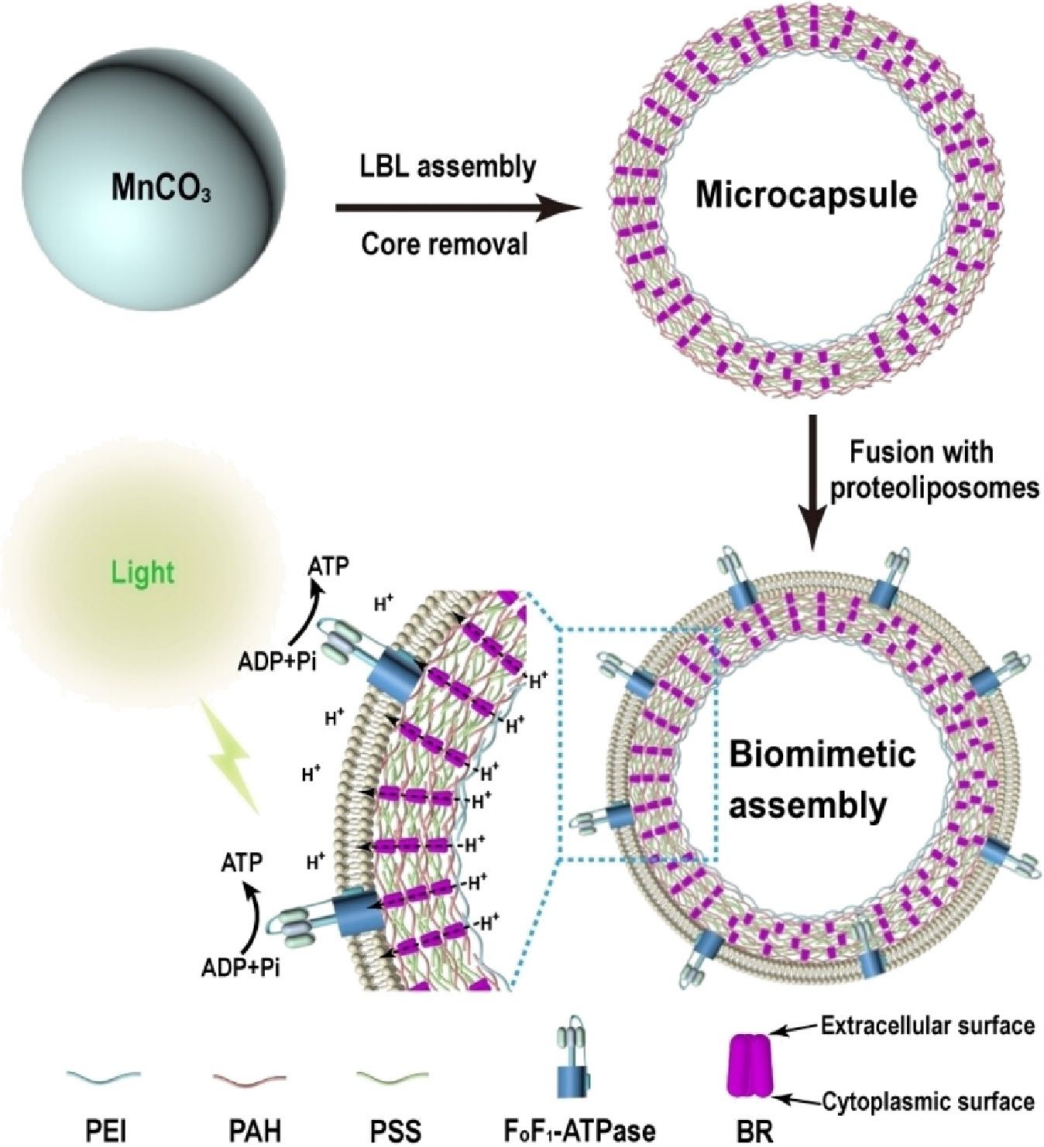
Nanoarchitectonics of bacteriorhodopsin for the enhancement of ATP generation using oriented bacteriorhodopsin microcapsules coated with F_o_F_1_-ATPase molecular motors via LbL assembly. This structure enables directional proton transfer. Figure 30 was reproduced from [308], Z. Li et al., “Oriented Nanoarchitectonics of Bacteriorhodopsin for Enhancing ATP Generation in a F_o_F_1_-ATPase-Based Assembly System”, *Angew. Chem. Int. Ed*., with permission from John Wiley and Sons. Copyright © 2022 Wiley-VCH GmbH. This content is not subject to CC BY 4.0.

As evidenced by the metabolism of photosynthetic organisms, enhancing energy conversion by optimizing the proton gradient represents a compelling research objective within the field of nanoarchitectonics. In the study of bacteriorhodopsin for the enhancement of ATP generation, Li, Dong, and colleagues created oriented bacteriorhodopsin microcapsules coated with F_o_F_1_-ATPase molecular motors via LbL assembly (Figure 30) [308]. This structure enables directional proton transfer. The nanoarchitectonics of bacteriorhodopsin and ATP synthase into an artificial biomimetic system permits the occurrence of cascade reactions and ATP synthesis under illumination. In the presence of light, bacteriorhodopsin undergoes a conformational change that facilitates radial proton transfer. This results in the creation of a higher proton gradient, which in turn enhances ATP synthesis by F_o_F_1_-ATPase. In this nanoarchitectonics structural design, bacteriorhodopsin maintains a highly uniform orientation with high loading capacity and enhanced phosphorylation through a layer-by-layer electrostatic adsorption-based approach. Moreover, the incorporation of optically aligned quantum dots markedly enhances light-induced phosphorylation. The directional ejection of protons from the microcapsules results in an increased proton gradient, thereby promoting ATP synthesis by F_o_F_1_-ATPase. The creation of such artificial hybrid nanoarchitectonics paves the way for new avenues of research into increasing the efficiency of solar energy conversion into ATP.

**Figure 31:**
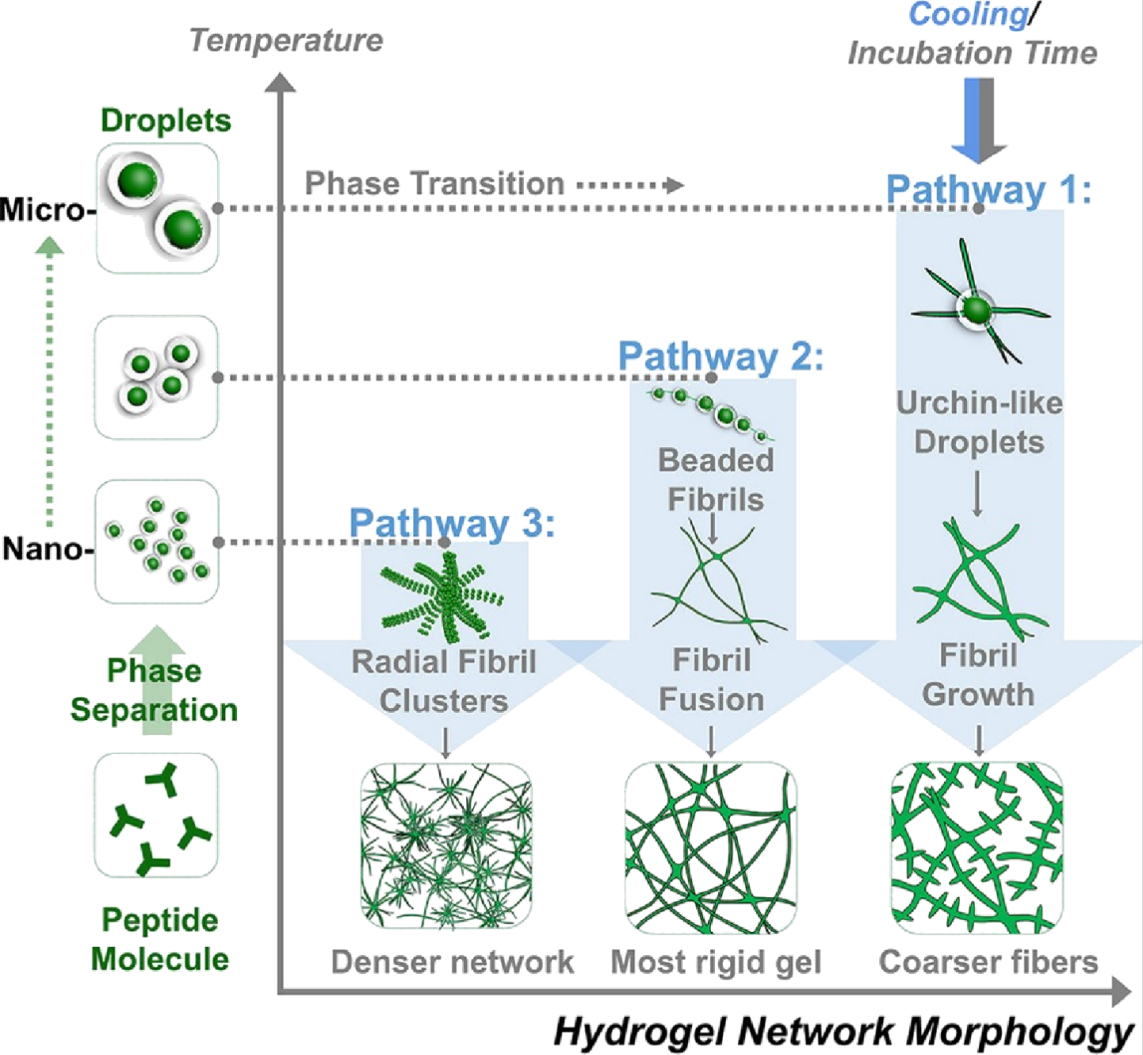
Nanoarchitectonics of hydrogel networks by adjusting the solute-rich metastable droplets formed by liquid–liquid phase separation with carboxybenzyl-protected diphenylalanine, a prototypical self-assembling peptide, as a model in which, by controlling the size and number of droplets, three distinct types of prefibrils were obtained including sea urchin-like fibrils, beaded spindle-like fibrils, and radial fibril clusters. Figure 31 was reprinted from [312], *Matter*, vol. 6, by P. Zhou; R. Xing; Q. Li; J. Li; C. Yuan; X. Yan, “Steering phase-separated droplets to control fibrillar network evolution of supramolecular peptide hydrogels“, pages 1945–1963, Copyright (2023), with permission from Elsevier. This content is not subject to CC BY 4.0.

The phenomenon of liquid–liquid phase separation has been the subject of considerable interest in the context of elucidating biological functions and fabricating structures [309–311]. It has been postulated that significant processes, including the formation of structurally disparate droplets, prefibrils, and the evolution of fibrillar networks, are attributable to liquid–liquid phase separation, wherein phase transitions occur within discrete droplets. Hydrogels with disparate assembly pathways and mechanical properties are produced, offering a means of biomaterial design. It is of great importance to be able to control the morphology of the non-covalently cross-linked network of supramolecular hydrogels in order to be able to tailor the mechanical properties of biomaterials. In the study by Li, Yuan, and Yan and colleagues, the evolution of fibril networks was observed, which in turn controlled the mechanical properties of hydrogels [312]. This was achieved by adjusting the solute-rich metastable droplets formed by liquid–liquid phase separation (Figure 31). In this study, carboxybenzyl-protected diphenylalanine, a prototypical self-assembling peptide, was selected as a model. The morphology of the network was controlled by the creation of metastable droplets, which were formed by liquid–liquid phase separation. By controlling the size and number of droplets, three distinct types of prefibrils were obtained, namely, sea urchin-like fibrils, beaded spindle-like fibrils, and radial fibril clusters. This series of intermediate polymorphs gives rise to bifurcating self-assembly pathways and dynamic supramolecular gelation behavior. The resulting gel networks display a range of fibril diameters and network densities, as well as a diverse spectrum of mechanical strengths that are markedly influenced by thermal history. As demonstrated in this study, by modulating the intermolecular interactions within the metastable droplets and the structural evolution of liquid–liquid phase separation, hydrogels with specific properties can be generated from peptide self-assembly. The concept of liquid–liquid phase separation will prove to be a significant area of interest, both in terms of understanding biological phenomena and in the creation of functional nanostructures.

Biomaterials are capable of performing a variety of sophisticated functions. The further assembly of these materials into advanced functional organizations using nanoarchitectonics represents a valuable method for the creation of more advanced functional materials. In numerous instances, the soft signal responsiveness necessary for biological functions is associated. It is of significant value to consider the subject of soft materials nanoarchitectonics in relation to the properties and functions of biomaterials. Furthermore, research is being conducted with the aim of developing a more fundamental understanding of functional biomaterials. The most exciting aspect of biomaterials nanoarchitectonics is the ability to assemble components with advanced functions into even more sophisticated organizational systems. This represents a promising avenue for the creation of functional structures by leveraging the inherent functionality observed in natural systems.

### A few other examples: 2D soft materials nanoarchitectonics

The preceding sections have examined the field of soft materials nanoarchitectonics as it pertains to a range of materials, including liquid crystals, polymers, gels, and biomolecules. It is possible that there are indications of the significance of nanoarchitectonics in examples that do not fall into these categories. It is not feasible to provide an exhaustive account of all relevant examples. The following section presents a selection of nanoarchitectonics-related examples of two-dimensional structures.

**Figure 32:**
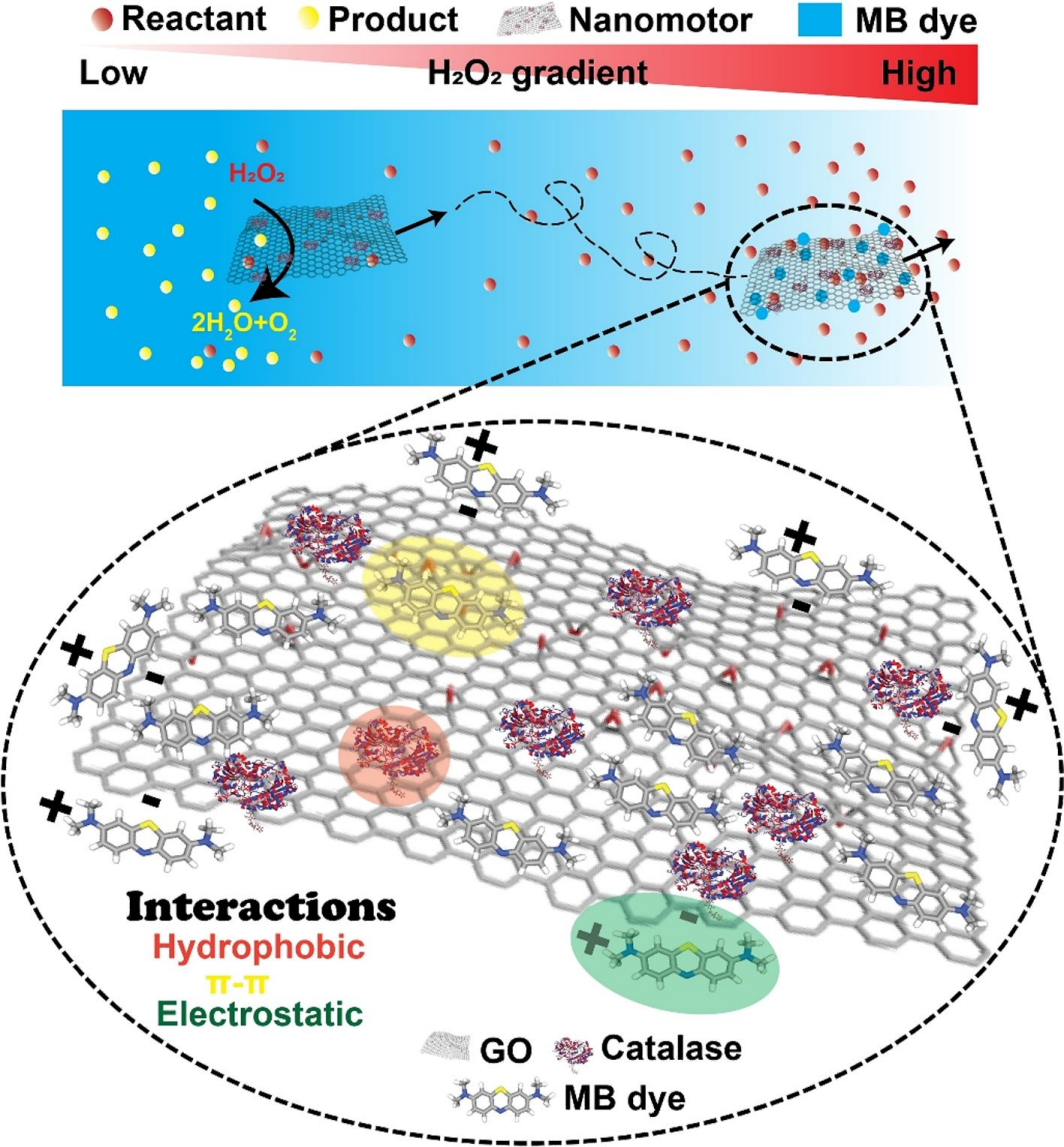
2D active nanobots based on soft nanoarchitectonics: enzyme-driven 2D nanobots based on graphene oxide through non-covalent interactions. Figure 32 was reproduced from [313], M. Mathesh et al., “2D Active Nanobots Based on Soft Nanoarchitectonics Powered by an Ultralow Fuel Concentration”, *Angew. Chem. Int. Ed.*, with permission from John Wiley and Sons. Copyright © 2021 Wiley-VCH GmbH. This content is not subject to CC BY 4.0.

Although two-dimensional materials such as graphene appear to possess a high degree of rigidity, they can in fact exhibit a high degree of mouldability when combined with materials that possess soft functionalities. One potential avenue of research is hybrid nanoarchitectonics with enzyme catalysts. Enzyme catalysts that power micro/nanomotors have significant potential for a diverse range of applications, including biomedical and environmental remediation. However, they are based on complex three-dimensional architectures and have limited surface area accessible to the catalytic sites, which results in suboptimal efficiency. Mathesh and colleagues constructed enzyme-driven 2D nanobots based on graphene oxide through non-covalent interactions (Figure 32) [313]. Catalase was immobilized on graphene oxide nanosheets (referred to as 2D nanobots) through non-covalent interactions. The 2D nanobots are capable of propelling the nanosheets in the presence of ultra-low concentrations of H_2_O_2_ as a fuel source. The velocity of the 2D nanobots is observed to increase with rising fuel concentration, yet it is noted to decline over time due to the enzyme reaction rate. The 2D nanobots exhibited efficient positive chemotaxis behavior and demonstrated the capacity to swim against gravity due to solute buoyancy. The two-dimensional characteristics of the nanobots are fully utilized, allowing them to move freely with the aid of buoyancy without the need for external forces. As a proof of concept, the 2D nanobots were used for the on-the-fly removal of methylene blue dye, thereby demonstrating the viability of a method for environmental remediation. It is anticipated that a diverse array of applications will emerge, spanning biomedical and plant nanobiotechnology. These may include active motion-based delivery to tumor microenvironments and site-specific delivery of cargo molecules to cell organelles.

**Figure 33:**
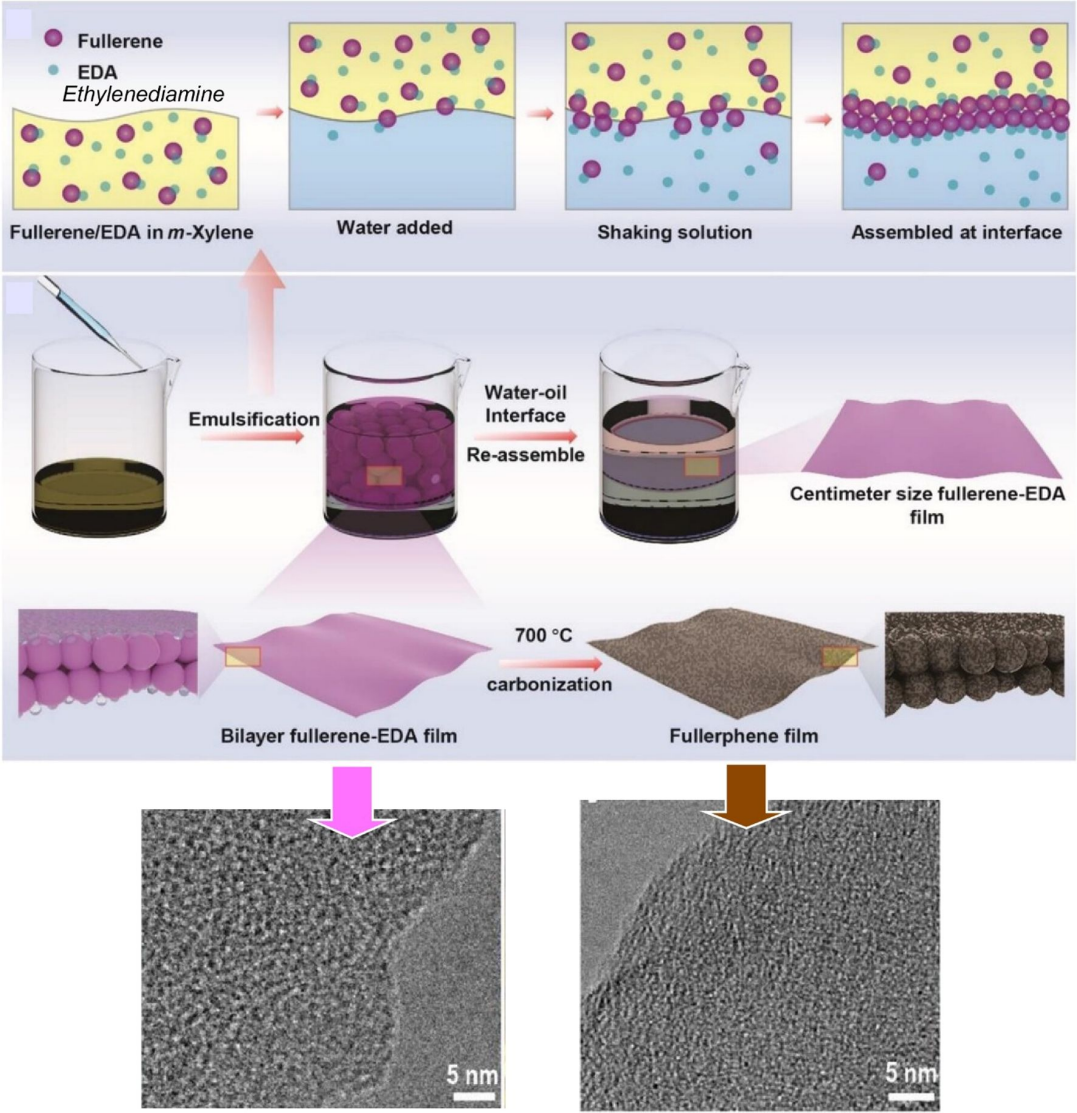
Nanoarchitectonics of a two-dimensional graphene-like material (fullerphene nanosheet) as a molecularly thin, nitrogen-doped two-dimensional carbon film, derived from zero-dimensional fullerene. Figure 33 was adapted from [314], J. Song et al., “Fullerphene Nanosheets: A Bottom-Up 2D Material for Single-Carbon-Atom-Level Molecular Discrimination”, *Advanced Materials Interfaces*, with permission from John Wiley and Sons. Copyright © 2022 Wiley-VCH GmbH. This content is not subject to CC BY 4.0.

Additionally, graphene-like two-dimensional materials can be fabricated through the use of soft materials nanoarchitectonics, such as interfacial self-assembly. Song et al. achieved the fabrication of a two-dimensional graphene-like material derived from zero-dimensional fullerene (Figure 33) [314]. The resulting two-dimensional material was designated a “fullerphene nanosheet”. It is a molecularly thin, nitrogen-doped two-dimensional carbon film. When a fullerene C_60_-ethylenediamine thin film assembled by bottom-up nanoarchitectonics at a liquid–liquid interface is thermally annealed at 700 °C, a nitrogen-doped ultrathin carbon film fullerphene is formed. The thickness is that of two fullerene molecules, yet it is distinguished by a hierarchical micro/mesoporous structure on its surface. In other words, fullerphene is an ultrafine porous nanostructure in which sp^2^-bonded C atoms are doped with pyrrole and N atoms, mainly quaternary N. The surface allows for selective and repeatable adsorption and desorption of low-molecular-weight carboxylic acid vapors through non-covalent interactions. The material can be immobilized on a quartz crystal microbalance (QCM) substrate for the purpose of gas sensing. It was demonstrated that the compound exhibited enhanced sensitivity to formic acid vapor in comparison to other prevalent low-molecular-weight carboxylic acid molecules. The enhanced sensitivity to formic acid in the gaseous phase is presumably attributable to the substantial surface area and pore volume afforded by the hierarchical micro/mesoporous structure. The adsorption of acids is dependent not only on the acidity of the analyte, but also on the molecular size. It is possible to achieve single-atom-level discrimination between formic acid and acetic acid, thereby enabling molecular discrimination based on the dimensions of the analyte at the single-atom level.

**Figure 34:**
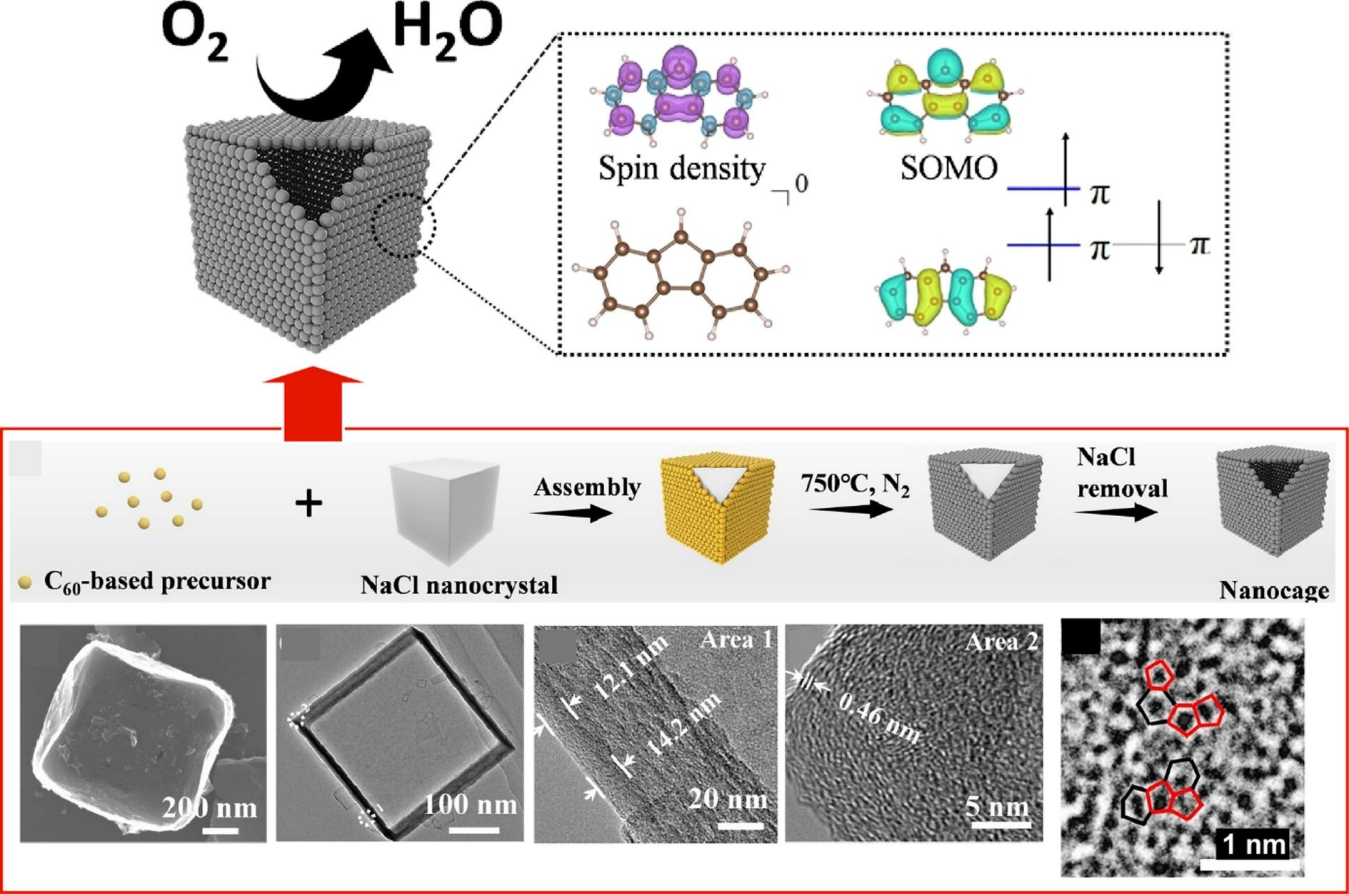
Nanoarchitectonics of a caged carbon catalyst using NaCl as a template and C_60_-containing pentagonal rings as the carbon source, with the aid of ethylenediamine, which also serves as a nitrogen source, featured with rich pentagon carbon structures and increase of electron spins for conducive to the induction of singly occupied molecular orbitals with remarkable activity for the oxygen reduction reaction: mechanism, processes, and images. Figure 34 was adapted from [318] (© 2024 G. Chen et al., *Angewandte Chemie International Edition*, published by Wiley-VCH GmbH, distributed under the terms of the Creative Commons Attribution 4.0 International License, https://creativecommons.org/licenses/by/4.0).

Furthermore, research is being conducted regarding the potential of nanoarchitectonics at the molecular framework level, utilizing high-resolution techniques. Such approaches have been conducted at the molecular level in on-surface synthesis in two-dimensional planes [315–317]. In contrast, Chen, Nakamura, and colleagues have achieved the controlled synthesis of five-membered carbon ring structures (pentagonal structures) in bulk synthesis, known as template synthesis, in their study on pentagon-rich caged carbon catalysts (Figure 34) [318]. The caged carbon catalyst was synthesized, and the mechanism underlying its high-performance oxygen reduction reaction was investigated. In this approach, a caged cubic carbon catalyst was synthesized using NaCl as a template and C_60_-containing pentagonal rings as the carbon source, with the aid of ethylenediamine, which also serves as a nitrogen source. The number of pentagons in the two-dimensional plane of the cube was increased through the introduction of nitrogen doping and subsequent annealing. It can be stated that annealing the caged cubic carbon catalyst at a high temperature in a nitrogen atmosphere results in the removal of the doped pyridine N and the generation of pentagons. Concomitantly, the number of electron spins is also observed to increase. The magnetism exhibited by catalysts with a higher pentagonal concentration is conducive to the induction of singly occupied molecular orbitals, thereby contributing to enhanced performance. The prepared catalysts display remarkable activity for the ORR under acidic electrolyte conditions. The relationship between the pentagonal structure, the number of spins, and the catalytic activity is clarified, and it is demonstrated that the enhancement in activity is contingent on the presence of spins. In other words, the origin of the ORR activity of pentagon-containing carbon catalysts can be attributed to the spins that are localized in the pentagonal rings. The results of the density functional theory calculations corroborate the hypothesis that spins are responsible for the enhancement in activity. The singly occupied molecular orbital of pentagon-containing molecules features a π orbital and is characterized by a low energy level, which facilitates effective oxygen adsorption. In other words, the local spin density of the pentagon plays a pivotal role in the adsorption of O_2_, the initial step of ORR. This study emphasizes the significance of pentagons with electronic spins in the development of efficient carbon-based catalysts for ORR, and introduces a novel principle for catalyst design. The interaction between the electron spin and oxygen molecules in non-platinum catalysts, particularly carbon-based catalysts, exerts a considerable influence on the catalytic performance of ORR. This property can be controlled by nanoarchitectonics at the molecular structure level. This study presents a promising approach to this end, introducing a five-membered ring structure with spin into graphitic carbon.

In addition to conventional soft materials, such as liquid crystals, polymers, gels, and biomolecules, nanoarchitectonics, which enables the fabrication of flexible and soft functional organizations, is a well-established field of research. To illustrate, a number of examples of nanoarchitectonics in relation to two-dimensional structures were presented. The presentation includes several examples of innovative, highly functional structures. The combination of graphene oxide and enzymes through hybrid nanoarchitectonics can facilitate the creation of microrobots that utilize two-dimensional materials for flight. The creation of new fullerene films via interface nanoarchitectonics involves the conversion of fullerenes into two-dimensional carbon films. The deliberate creation of carbon pentagon structures has the potential to enhance the catalytic function of the oxygen reduction reaction, due to their distinctive spin properties. By extending the scope of this research in this way, it can significantly expand the possibilities of nanoarchitectonics and drive the development of innovative functions.

## Conclusion and Future Perspectives

This review has examined the current state of soft materials nanoarchitectonics in light of the ongoing developments in materials sciences, which have seen a shift from hard to soft materials and from the macroscale to the nanoscale. Due to the limitations of space, the review has focused only on liquid crystals, polymers, gels, and biological materials, as well as papers claiming to be nanoarchitectonics and related works. The characteristics of each are outlined as follows. Liquid crystals are soft materials that exhibit moderate fluidity and order and can serve as a central material in soft materials nanoarchitectonics, as evidenced by their potential for developing stimuli-responsive materials. For polymers, the design, synthesis and organization have been the subject of considerable research and development, and a range of biopolymers can be sourced from natural materials. Based on such knowledge, nanoarchitectonics of polymers is being carried out in a variety of ways, employing techniques such as LbL assembly to control the assembly and structures. Polymer materials have advantages to obtain specific functions for demands. In gel nanoarchitectonics, the ability to combine components with disparate characteristics in order to produce complementary or concerted effects is facilitated by the provision of a modulative environment. Conversely, biomaterials have been developed to possess advanced functionalities as components. The further assembly of biomaterial components into advanced functional organizations represents a valuable method for the creation of even more advanced functional materials. These approaches facilitate the creation of diverse potential applications. The following examples illustrate the potential applications of these approaches: organic semiconductor devices, electrochemical catalysts, organic thin film sensors, solar power generation, plastic crystal solid electrolytes, microactuators, intelligent light-responsive materials, self-repairing composite materials, enzyme cascade sensors, materials that enhance the healing of diabetic bone defects, and materials with strong bactericidal capabilities against resistant bacteria. In addition, soft materials nanoarchitectonics has yielded a number of groundbreaking results. These include the development of microrobots comprising two-dimensional materials, fullerphene films with atomic-level recognition capabilities, and the creation of carbon pentagons that can control spin and oxygen reduction reactions. Of course, these examples are not exhaustive. Various examples on nanoarchitectonics approaches can be seen in recent review articles [319–323].

As shown above, soft materials nanoarchitectonics offers a vast array of materials designs, specific functions, and potential applications. Even when considering a restricted selection of case studies, the potential for innovation is indeed vast. Consequently, nanoarchitectonics for soft materials, which possesses flexible structures and numerous uncertainties, represents more challenging targets. The examples selected for discussion as soft materials nanoarchitectonics are also highly diverse in terms of their components, structures, assembly patterns and functions. Because nanoarchitectonics approaches often use several process together, various types of interactions such as electrostatic interaction, coordination, and hydrogen bonding are involved. These interactions are applied with combined ways in many cases. Based on these various possibilities, functional materials systems have been continuously created [324–327]. As a result, they are subjects that cannot be easily unified. The utilization of the knowledge and technology of researchers themselves or those documented in the literature may prove insufficient to fully harness the potential of soft materials nanoarchitectonics. The optimal solution to this problem would be the utilization of artificial intelligence, which has undergone significant advancements in recent years. The application of machine learning [328–332] and materials informatics [333–335] has led to significant advances in the field of artificial intelligence, which is making a notable contribution to the materials science community. With regard to nanoarchitectonics, a number of potential avenues for integration with materials informatics have been proposed [336–337]. It is our contention that the active introduction of artificial intelligence will prove to be a pivotal factor in the advancement of soft materials nanoarchitectonics, which is characterized by its flexibility, diversity and considerable potential, as evidenced by the findings presented here.

This review presents a number of examples of soft materials nanoarchitectonics and demonstrates the considerable potential of this field of research. In addition, it has proposed that the integration of artificial intelligence would prove an effective means of facilitating further development. Furthermore, the integration of processes at the nanoscale and the macroscale is essential for the realization of practical mass production. Of course, developments of characterization methods to analyze intermediate processes in addition to product analyses would become crucial. As overall conclusion, it is highly anticipated that soft materials nanoarchitectonics will continue to evolve significantly in the future.

## Data Availability

Data sharing is not applicable as no new data was generated or analyzed in this study.

## References

[1] Nocera A, Perroni C A, Ramaglia V M, Cataudella V (2016). Beilstein J Nanotechnol.

[2] Saito K, Yamamura Y (2023). Bull Chem Soc Jpn.

[3] O'Neill R T, Boulatov R (2024). Angew Chem, Int Ed.

[4] Long C, Wang R, Wang Y, Lan H, Zhu X, Zhang Y-F (2025). Prog Mater Sci.

[5] Jiang L, Sha Z, Zheng Y, Zhu R, Yu C, Chen Q, Ran R, Cui W (2025). Prog Mater Sci.

[6] Acet Ö, Kirsanov P, Önal Acet B, Halets-Bui I, Shcharbin D, Ceylan Cömert Ş, Odabaşı M (2024). Beilstein J Nanotechnol.

[7] Yamanoi Y, Hattori M (2024). Bull Chem Soc Jpn.

[8] Kaneda T, Kato K, Ohtani S, Ogoshi T (2024). Bull Chem Soc Jpn.

[9] Bai H Y, Zhu Q L, Cheng H L, Wen X L, Wang Z J, Zheng Q, Wu Z L (2025). Mater Horiz.

[10] Vashishtha G, Chauhan S, Yadav N, Chhabra D, Gupta M K, Kumar R, Li Z (2025). Int J Adv Manuf Technol.

[11] Vetter I R, Wittinghofer A (2001). Science.

[12] Jordan P, Fromme P, Witt H T, Klukas O, Saenger W, Krauß N (2001). Nature.

[13] Ferreira K N, Iverson T M, Maghlaoui K, Barber J, Iwata S (2004). Science.

[14] Yamamoto Y, Nakano S, Shigeta Y (2023). Bull Chem Soc Jpn.

[15] Miura T, Malla T R, Brewitz L, Tumber A, Salah E, Lee K J, Terasaka N, Owen C D, Strain-Damerell C, Lukacik P (2024). Bull Chem Soc Jpn.

[16] Quader S, Kataoka K, Cabral H (2022). Adv Drug Delivery Rev.

[17] Mohanan S, Sathish C I, Adams T J, Kan S, Liang M, Vinu A (2023). Bull Chem Soc Jpn.

[18] Yang W, Mixich L, Boonstra E, Cabral H (2023). Adv Healthcare Mater.

[19] Syaifie P H, Nasution M A F, Rahmawati I, Saepudin E, Ivandini T A (2024). Bull Chem Soc Jpn.

[20] Song J, Kawakami K, Ariga K (2025). Adv Colloid Interface Sci.

[21] Guo D, Shibuya R, Akiba C, Saji S, Kondo T, Nakamura J (2016). Science.

[22] Zhang E, Zhu Q, Huang J, Liu J, Tan G, Sun C, Li T, Liu S, Li Y, Wang H (2021). Appl Catal, B.

[23] Shinde P A, Abbas Q, Chodankar N R, Ariga K, Abdelkareem M A, Olabi A G (2023). J Energy Chem.

[24] Yoshimune W (2024). Bull Chem Soc Jpn.

[25] Hu Z, Gao Y, Ji S, Mae M, Imaizumi T (2024). Appl Energy.

[26] Fukushima T, Higashi M, Yamauchi M (2023). Bull Chem Soc Jpn.

[27] Ikeda A, Hunge Y M, Teshima K, Uetsuka H, Terashima C (2024). Energy Fuels.

[28] Wang K, Oe H, Nakaji Y, Wang Y, Nakaji-Hirabayashi T, Tsubaki N (2024). Chem.

[29] Takahara C, Iwasaki S, Miyake Y, Yumura T, Imoto H, Naka K (2024). Bull Chem Soc Jpn.

[30] Tran N Q, Vu N H, Yu J, Nguyen K V P, Tran T T N, Truong T-K, Peng L, Le T A, Kawazoe Y (2024). J Energy Chem.

[31] Povie G, Segawa Y, Nishihara T, Miyauchi Y, Itami K (2017). Science.

[32] Sun Z, Ikemoto K, Fukunaga T M, Koretsune T, Arita R, Sato S, Isobe H (2019). Science.

[33] Sugiyama M, Akiyama M, Yonezawa Y, Komaguchi K, Higashi M, Nozaki K, Okazoe T (2022). Science.

[34] Yasukawa Y (2024). Bull Chem Soc Jpn.

[35] Nagashima Y (2024). Bull Chem Soc Jpn.

[36] Hattori T (2024). Bull Chem Soc Jpn.

[37] Okuda S, Ikai T, Okutsu H, Ando M, Hattori M, Ishidate R, Yashima E (2024). Angew Chem, Int Ed.

[38] Narikiyo H, Gon M, Tanaka K, Chujo Y (2024). Bull Chem Soc Jpn.

[39] Uchiyama M, Ohira N, Yamashita K, Sagawa K, Kamigaito M (2024). Nat Chem.

[40] Kamigaito M (2024). Bull Chem Soc Jpn.

[41] Akiyama M, Yasuda Y, Kisoi D, Kusakabe Y, Kaji H, Imahori H (2024). Bull Chem Soc Jpn.

[42] Takezawa H, Iizuka K, Fujita M (2024). Angew Chem, Int Ed.

[43] Nakamura T, Kondo Y, Ohashi N, Sakamoto C, Hasegawa A, Hu S, Truong M A, Murdey R, Kanemitsu Y, Wakamiya A (2024). Bull Chem Soc Jpn.

[44] Zhang H, Masui Y, Masai H, Terao J (2024). Bull Chem Soc Jpn.

[45] Hama R, Ulziibayar A, Reinhardt J W, Watanabe T, Kelly J, Shinoka T (2023). Biomolecules.

[46] Watanabe S, Oyaizu K (2023). Bull Chem Soc Jpn.

[47] Morishita T, Maitani I, Michiura T, Suzuki N, Suzuki T, Akamatsu K, Minami H (2024). ACS Appl Mater Interfaces.

[48] Fujii S (2024). Curr Opin Colloid Interface Sci.

[49] Yamamoto Y, Heah W Y, Tashiro K (2024). Mater Horiz.

[50] Kato T, Uchida J, Ichikawa T, Sakamoto T (2018). Angew Chem, Int Ed.

[51] Uchida J, Soberats B, Gupta M, Kato T (2022). Adv Mater (Weinheim, Ger).

[52] Yoshizawa A (2024). Crystals.

[53] Yoshida S, Morikawa S, Ueda K, Kaneko K, Hanasaki T, Akagi K (2024). ACS Appl Mater Interfaces.

[54] Matsukizono H, Sakamoto Y, Okumura Y, Kikuchi H (2024). J Phys Chem Lett.

[55] Creton C (2017). Macromolecules.

[56] Tamate R, Ueki T (2023). Chem Rec.

[57] Shintani Y, Katagiri H, Ikeda M (2024). Adv Funct Mater.

[58] Fujiyabu T, Qi P, Yoshie K, Fujisawa A, Tsuji Y, Chandel A K S, Madhavikutty A S, Inagaki N F, Ohta S, Fujishiro M (2024). Chem Eng J.

[59] Kimura K, Marangon V, Fukuda T, Suzuki M, Soontornnon N, Tominaga Y, Hassoun J (2024). Chem Commun.

[60] Datta S, Kato Y, Higashiharaguchi S, Aratsu K, Isobe A, Saito T, Prabhu D D, Kitamoto Y, Hollamby M J, Smith A J (2020). Nature.

[61] Takeuchi Y, Ohkura K, Nishina Y (2023). Bull Chem Soc Jpn.

[62] Mieda E, Morishima Y, Watanabe T, Miyake H, Shinoda S (2023). Bull Chem Soc Jpn.

[63] Chen J, Tsuchida A, Malay A D, Tsuchiya K, Masunaga H, Tsuji Y, Kuzumoto M, Urayama K, Shintaku H, Numata K (2024). Nat Commun.

[64] Chen N, Hu M, Gou L, Tan L, Zhao D, Feng H (2024). Bull Chem Soc Jpn.

[65] Horike S (2023). Bull Chem Soc Jpn.

[66] Wang W, Chen D, Li F, Xiao X, Xu Q (2024). Chem.

[67] Miyazaki S, Ogiwara N, Nagasaka C A, Takiishi K, Inada M, Uchida S (2024). Bull Chem Soc Jpn.

[68] Guan J, Koizumi K, Fukui N, Suzuki H, Murayama K, Toyoda R, Maeda H, Kamiya K, Ohashi K, Takaishi S (2024). ACS Catal.

[69] Rani R, Ueda T, Saeki K, Toda K, Ohira S-I (2024). Bull Chem Soc Jpn.

[70] Geng K, He T, Liu R, Dalapati S, Tan K T, Li Z, Tao S, Gong Y, Jiang Q, Jiang D (2020). Chem Rev.

[71] Yang J, Huang L, You J, Yamauchi Y (2023). Small.

[72] Jiang D, Xu X, Bando Y, Alshehri S M, Eguchi M, Asahi T, Yamauchi Y (2024). Bull Chem Soc Jpn.

[73] Mabuchi H, Irie T, Sakai J, Das S, Negishi Y (2024). Chem – Eur J.

[74] Wang X, Wada Y, Shimada T, Kosaka A, Adachi K, Hashizume D, Yazawa K, Uekusa H, Shoji Y, Fukushima T (2024). J Am Chem Soc.

[75] Casalini S, Bortolotti C A, Leonardi F, Biscarini F (2017). Chem Soc Rev.

[76] Inkpen M S, Liu Z-F, Li H, Campos L M, Neaton J B, Venkataraman L (2019). Nat Chem.

[77] Farag A, Feeney T, Hossain I M, Schackmar F, Fassl P, Küster K, Bäuerle R, Ruiz-Preciado M A, Hentschel M, Ritzer D B (2023). Adv Energy Mater.

[78] Sun A, Tian C, Zhuang R, Chen C, Zheng Y, Wu X, Tang C, Liu Y, Li Z, Ouyang B (2024). Adv Energy Mater.

[79] Li M, Liu M, Qi F, Lin F R, Jen A K-Y (2024). Chem Rev.

[80] Kim M S, Ryu J-H, Deepika, Lim Y R, Nah I W, Lee K-R, Archer L A, Il Cho W (2018). Nat Energy.

[81] Oliveira O N, Caseli L, Ariga K (2022). Chem Rev.

[82] Negi S, Hamori M, Kubo Y, Kitagishi H, Kano K (2023). Bull Chem Soc Jpn.

[83] Ariga K (2023). Chem Mater.

[84] Terui R, Otsuki Y, Shibasaki Y, Fujimori A (2024). Bull Chem Soc Jpn.

[85] Lvov Y, Ariga K, Kunitake T (1994). Chem Lett.

[86] Decher G (1997). Science.

[87] Caruso F, Caruso R A, Möhwald H (1998). Science.

[88] Ariga K, Lvov Y, Decher G (2022). Phys Chem Chem Phys.

[89] Ariga K, Song J, Kawakami K (2024). Chem Commun.

[90] Imahori H (2023). Bull Chem Soc Jpn.

[91] Cai Z-X, Bolar S, Ito Y, Fujita T (2024). Nanoscale.

[92] Xia K, Yatabe T, Yonesato K, Kikkawa S, Yamazoe S, Nakata A, Ishikawa R, Shibata N, Ikuhara Y, Yamaguchi K (2024). Nat Commun.

[93] Kuzume A, Yamamoto K (2024). Bull Chem Soc Jpn.

[94] Saito K, Morita M, Okada T, Wijitwongwane R, Ogawa M (2024). Chem Soc Rev.

[95] Saitow K (2024). Bull Chem Soc Jpn.

[96] Phengdaam A, Phetsang S, Jonai S, Shinbo K, Kato K, Baba A (2024). Nanoscale Adv.

[97] Zheng L, Yunoki N, Suzuki N, Ogino K (2024). Bull Chem Soc Jpn.

[98] Bapathi K S R, Abdelbar M F, Jevasuwan W, Borse P H, Badhulika S, Fukata N (2024). Nano Energy.

[99] Zhao Z, Che Q, Chen Q, Wang K, Zhao K, Zhang C, He H, Wang X, Chen Y (2024). Bull Chem Soc Jpn.

[100] Sugimoto Y, Pou P, Abe M, Jelinek P, Pérez R, Morita S, Custance Ó (2007). Nature.

[101] Xing J, Takeuchi K, Kamei K, Nakamuro T, Harano K, Nakamura E (2022). Proc Natl Acad Sci U S A.

[102] Harano K, Nakamuro T, Nakamura E (2024). Microscopy (Oxford, U K).

[103] Onoe J, Noda Y, Wang Q, Harano K, Nakaya M, Nakayama T (2024). Sci Technol Adv Mater.

[104] Nakamuro T (2024). Bull Chem Soc Jpn.

[105] Okawa Y, Aono M (2001). Nature.

[106] Shirai Y, Minami K, Nakanishi W, Yonamine Y, Joachim C, Ariga K (2016). Jpn J Appl Phys.

[107] Kawai S, Krejčí O, Nishiuchi T, Sahara K, Kodama T, Pawlak R, Meyer E, Kubo T, Foster A S (2020). Sci Adv.

[108] Milano G, Aono M, Boarino L, Celano U, Hasegawa T, Kozicki M, Majumdar S, Menghini M, Miranda E, Ricciardi C (2022). Adv Mater (Weinheim, Ger).

[109] Oyamada N, Minamimoto H, Fukushima T, Zhou R, Murakoshi K (2024). Bull Chem Soc Jpn.

[110] Kazuma E, Jung J, Ueba H, Trenary M, Kim Y (2018). Science.

[111] Kimura K, Miwa K, Imada J, Imai-Imada M, Kawahara S, Takeya J, Kawai M, Galperin M, Kim Y (2019). Nature.

[112] Yamaguchi M, Tomás M de la H, Fujiwara A, Oketani R, Okubo K, Oka K, Tohnai N, Douhal A, Hisaki I (2024). Bull Chem Soc Jpn.

[113] Hwang D-W, Maekiniemi A, Singer R H, Sato H (2024). Nat Rev Genet.

[114] Hashikawa Y, Murata Y (2023). Bull Chem Soc Jpn.

[115] Otsuka H (2023). Gels.

[116] Zhao Z, Fukushima T, Zharnikov M (2023). J Phys Chem C.

[117] Yamagishi M, Yamamoto S, Okano K, Koshiba Y, Horike S, Ishida K, Horie M, Mori A (2024). Bull Chem Soc Jpn.

[118] Shi Q, Kajita S, Ohno N, Tanaka H, Yasuhara R, Fujiwara H, Uehara H (2024). Langmuir.

[119] Cherepakhin A, Zhizhchenko A, Khmelevskaia D, Logunov L, Kuchmizhak A, Makarov S (2024). Adv Opt Mater.

[120] Ariga K (2021). Nanoscale Horiz.

[121] Feynman R P (1960). Eng Sci.

[122] Roukes M (2001). Sci Am.

[123] Ariga K, Ji Q, Hill J P, Bando Y, Aono M (2012). NPG Asia Mater.

[124] Ariga K, Ji Q, Nakanishi W, Hill J P, Aono M (2015). Mater Horiz.

[125] Ariga K, Nishikawa M, Mori T, Takeya J, Shrestha L K, Hill J P (2019). Sci Technol Adv Mater.

[126] Aono M, Ariga K (2016). Adv Mater (Weinheim, Ger).

[127] Cao L, Huang Y, Parakhonskiy B, Skirtach A G (2022). Nanoscale.

[128] Ariga K (2023). Beilstein J Nanotechnol.

[129] Ariga K, Li J, Fei J, Ji Q, Hill J P (2016). Adv Mater (Weinheim, Ger).

[130] Ariga K, Jia X, Song J, Hill J P, Leong D T, Jia Y, Li J (2020). Angew Chem, Int Ed.

[131] Laughlin R B, Pines D (2000). Proc Natl Acad Sci U S A.

[132] Ariga K, Fakhrullin R (2022). Bull Chem Soc Jpn.

[133] Ariga K (2024). Bull Chem Soc Jpn.

[134] Nakanishi W, Minami K, Shrestha L K, Ji Q, Hill J P, Ariga K (2014). Nano Today.

[135] Komiyama M, Mori T, Ariga K (2018). Bull Chem Soc Jpn.

[136] Chen G, Sciortino F, Takeyasu K, Nakamura J, Hill J P, Shrestha L K, Ariga K (2022). Chem – Asian J.

[137] Hikichi R, Tokura Y, Igarashi Y, Imai H, Oaki Y (2023). Bull Chem Soc Jpn.

[138] Li H, Jia Y, Bai S, Peng H, Li J (2024). Adv Colloid Interface Sci.

[139] Chen G, Shrestha L K, Ariga K (2021). Molecules.

[140] Wang Y, Niu D, Ouyang G, Liu M (2022). Nat Commun.

[141] Datta K K R (2023). ChemNanoMat.

[142] Ariga K (2024). Small.

[143] Song J, Jancik-Prochazkova A, Kawakami K, Ariga K (2024). Chem Sci.

[144] Nayak A, Unayama S, Tai S, Tsuruoka T, Waser R, Aono M, Valov I, Hasegawa T (2018). Adv Mater (Weinheim, Ger).

[145] Eguchi M, Nugraha A S, Rowan A E, Shapter J, Yamauchi Y (2021). Adv Sci.

[146] Shioya N, Mori T, Ariga K, Hasegawa T (2024). Jpn J Appl Phys.

[147] Gawade V K, Jadhav R W, Bhosale S V (2024). Chem – Asian J.

[148] Ariga K, Song J, Kawakami K (2024). Phys Chem Chem Phys.

[149] Shen X, Song J, Sevencan C, Leong D T, Ariga K (2022). Sci Technol Adv Mater.

[150] Yoshida T, Ogawa M (2022). Nanoscale.

[151] Xing R, Yuan C, Fan W, Ren X, Yan X (2023). Sci Adv.

[152] Chang R, Zhao L, Xing R, Li J, Yan X (2023). Chem Soc Rev.

[153] Xu Y, Yu F, Jia Y, Xu X, Li J (2024). Angew Chem, Int Ed.

[154] Chen G, Sciortino F, Ariga K (2021). Adv Mater Interfaces.

[155] Sharma D, Choudhary P, Kumar S, Krishnan V (2023). Small.

[156] Lu X, Yan K, Yu Z, Wang J, Liu R, Zhang R, Qiao Y, Xiong J (2024). ChemSusChem.

[157] Sharma D, Choudhary P, Mittal P, Kumar S, Gouda A, Krishnan V (2024). ACS Catal.

[158] Wu Y-L, Tang P-F, Zhang Q, Yan Y-T, Zhang S, Yang G-P, Wang Y-Y (2024). Appl Organomet Chem.

[159] Liu X, Chen T, Xue Y, Fan J, Shen S, Hossain M S A, Amin M A, Pan L, Xu X, Yamauchi Y (2022). Coord Chem Rev.

[160] Kumar A, Choudhary P, Chhabra T, Kaur H, Kumar A, Qamar M, Krishnan V (2023). Mater Chem Front.

[161] Sharma M, Kumar A, Gill D, Jaiswal S, Patra A, Bhattacharya S, Krishnan V (2023). ACS Appl Mater Interfaces.

[162] Zhang X, Yang P (2024). Carbon.

[163] Sadanandan A M, Yang J-H, Devtade V, Singh G, Dharmarajan N P, Fawaz M, Lee J M, Tavakkoli E, Jeon C-H, Kumar P (2024). Prog Mater Sci.

[164] Ishihara S, Labuta J, Van Rossom W, Ishikawa D, Minami K, Hill J P, Ariga K (2014). Phys Chem Chem Phys.

[165] Chen G, Bhadra B N, Sutrisno L, Shrestha L K, Ariga K (2022). Int J Mol Sci.

[166] Vaghasiya J V, Mayorga-Martinez C C, Pumera M (2023). npj Flexible Electron.

[167] Alruwais R S, Adeosun W A (2024). J Inorg Organomet Polym Mater.

[168] Huang P, Wu W, Li M, Li Z, Pan L, Ahamad T, Alshehri S M, Bando Y, Yamauchi Y, Xu X (2024). Coord Chem Rev.

[169] Liu J, Zhou H, Yang W, Ariga K (2020). Acc Chem Res.

[170] Moradi R, Khalili N P, Septiani N L W, Liu C-H, Doustkhah E, Yamauchi Y, Rotkin S V (2022). Small.

[171] Kim S, Baek S, Sluyter R, Konstantinov K, Kim J H, Kim S, Kim Y H (2023). EcoMat.

[172] Javed A, Kong N, Mathesh M, Duan W, Yang W (2024). Sci Technol Adv Mater.

[173] Geravand M, Erfani Y, Nematpour N, Khosravani M, Rahimnia R, Adabi M (2024). Microchem J.

[174] Tsuchiya T, Nakayama T, Ariga K (2022). Appl Phys Express.

[175] Deepak D, Soin N, Roy S S (2023). Mater Today Commun.

[176] Baek S, Kim S, Han S A, Kim Y H, Kim S, Kim J N (2023). ChemNanoMat.

[177] Azzaroni O, Piccinini E, Fenoy G, Marmisollé W, Ariga K (2023). Nanotechnology.

[178] Halder S, Chakraborty C (2024). Nano Energy.

[179] Liu X, Chen T, Gong Y, Li C, Niu L, Xu S, Xu X, Pan L, Shapter J G, Yamauchi Y (2021). J Photochem Photobiol, C.

[180] Nguyen N T K, Lebastard C, Wilmet M, Dumait N, Renaud A, Cordier S, Ohashi N, Uchikoshi T, Grasset F (2022). Sci Technol Adv Mater.

[181] Dhanabal R, Kasinathan D, Mahalingam A, Madhuri K, Bose A C, Dey S R (2023). J Mater Sci: Mater Electron.

[182] Kim D, Lim H, Kim S H, Lee K N, You J, Ryu D Y, Kim J (2024). J Mater Chem A.

[183] Abdulrhman M, Abdel-Aal S K, Bain C A, Raptis D, Bernal-Texca F, Wlodarczyk K L, Hand D P, Martorell J, Marques-Hueso J (2024). Appl Phys A: Mater Sci Process.

[184] Huang H, Yan M, Yang C, He H, Jiang Q, Yang L, Lu Z, Sun Z, Xu X, Bando Y (2019). Adv Mater (Weinheim, Ger).

[185] Chen G, Singh S K, Takeyasu K, Hill J P, Nakamura J, Ariga K (2022). Sci Technol Adv Mater.

[186] Thmaini N, Charradi K, Ahmed Z, Chtourou R, Aranda P (2023). Appl Clay Sci.

[187] Liang H, Zhu X, Chen Y, Cheng J (2024). Appl Phys A: Mater Sci Process.

[188] Su Y, Ding X, Yuan J (2024). Int J Hydrogen Energy.

[189] Allwyn N, Ambrose B, Kathiresan M, Sathish M (2023). ACS Appl Energy Mater.

[190] Gokulnath S, Krishnan S, Yadav V, Sathish M (2024). Energy Fuels.

[191] Koralkar N, Mehta S, Upadhyay A, Patel G, Deshmukh K (2024). J Inorg Organomet Polym Mater.

[192] Shen J, Tian W, Liu S, Pan H, Yang C, Quan H, Zhu S (2024). ACS Nano.

[193] Wang H, Cheng P, Wu B, Yan Y, Schaaf P, Sofer Z, Wang D (2024). Adv Funct Mater.

[194] Na J, Zheng D, Kim J, Gao M, Azhar A, Lin J, Yamauchi Y (2022). Small.

[195] Shinde P A, Chodankar N R, Kim H-J, Abdelkareem M A, Ghaferi A A, Han Y-K, Olabi A G, Ariga K (2023). ACS Energy Lett.

[196] Feng T, Luo X, Liu Z, Liu X, Yan X, Li G, Zhang W, Wang K (2024). Appl Phys Rev.

[197] Mathan S, Selvaraj M, Assiri M A, Kandiah K, Rajendran R (2024). Surf Interfaces.

[198] Mishra P K, Shrestha K R, Oli H B, Shrestha T, Joshi L P, Shrestha R L, Bhattarai D P (2024). J Taiwan Inst Chem Eng.

[199] Khan A H, Ghosh S, Pradhan B, Dalui A, Shrestha L K, Acharya S, Ariga K (2017). Bull Chem Soc Jpn.

[200] Kim J, Kim J H, Ariga K (2017). Joule.

[201] Ghosh K, Iffelsberger C, Konečný M, Vyskočil J, Michalička J, Pumera M (2023). Adv Energy Mater.

[202] Feng J-C, Li S-X, Zhang Z-P, An Y, Gao Q-S, Sun Z, Xia H (2024). Nano Energy.

[203] Wu H, Li J, Ji Q, Ariga K (2024). Sci Technol Adv Mater.

[204] Chuy G P, Muraro P C L, Viana A R, Pavoski G, Espinosa D C R, Vizzotto B S, da Silva W L (2022). J Inorg Organomet Polym Mater.

[205] Bhadra B N, Shrestha L K, Ariga K (2022). CrystEngComm.

[206] Jiao W, Liu P, Zhao Z, Zhang H, Zhang L (2023). J Electron Mater.

[207] Li Z, Chen M, Zhu W, Xin R, Yang J, Hu S, You J, Ryu D Y, Lim S-H, Li S (2024). Coord Chem Rev.

[208] Ullah H, Alomar T S, Tariq M, AlMasoud N, Bhatti M H, Ajmal M, Nazar Z, Nadeem M, Asif H M, Sohail M (2024). J Environ Chem Eng.

[209] Ferhan A R, Park S, Park H, Tae H, Jackman J A, Cho N-J (2022). Adv Funct Mater.

[210] Komiyama M (2023). Beilstein J Nanotechnol.

[211] Aziz T, Nadeem A A, Sarwar A, Perveen I, Hussain N, Khan A A, Daudzai Z, Cui H, Lin L (2023). Biomedicines.

[212] You Q Y, Hu M D, Qian H (2024). Adv Funct Mater.

[213] Song J, Jia X, Ariga K (2020). Small Methods.

[214] Hu W, Shi J, Lv W, Jia X, Ariga K (2022). Sci Technol Adv Mater.

[215] Jia X, Chen J, Lv W, Li H, Ariga K (2023). Cell Rep Phys Sci.

[216] Song J, Lyu W, Kawakami K, Ariga K (2024). Nanoscale.

[217] Dixit M, Shrestha L K, Ariga K, Pati F (2024). ACS Appl Nano Mater.

[218] Kumbhar P, Kolekar K, Khot C, Dabhole S, Salawi A, Sabei F Y, Mohite A, Kole K, Mhatre S, Jha N K (2023). J Controlled Release.

[219] Sutrisno L, Ariga K (2023). NPG Asia Mater.

[220] Song J, Kawakami K, Ariga K (2023). Curr Opin Colloid Interface Sci.

[221] Duan H, Wang F, Xu W, Sheng G, Sun Z, Chu H (2023). Dalton Trans.

[222] Xiao S, Sun G, Huang S, Lin C, Li Y (2024). Pharmaceutics.

[223] Schmidt-Mende L, Fechtenkötter A, Müllen K, Moons E, Friend R H, MacKenzie J D (2001). Science.

[224] Huang L, Wu H, Ding L, Caro J, Wang H (2024). Angew Chem, Int Ed.

[225] Xu J, Li R, Chen X, Zhang L, Li Y, Huang Y, Zhao T, Jia L (2024). Appl Surf Sci.

[226] Kumar N, Sahu S, Paul H, Rout M K, De J, Pal S K, Mishra P, Nayak A (2024). J Phys Chem B.

[227] Kumar N, Samal P P, Mahapatra A, De J, Pal S K, Mishra P, Nayak A (2023). Soft Matter.

[228] Wang C, Dong H, Hu W, Liu Y, Zhu D (2012). Chem Rev.

[229] Iwanaga T, Tanaka K, Kawano K (2024). Bull Chem Soc Jpn.

[230] Yamamoto Y, Kushida S, Okada D, Oki O, Yamagishi H, Hendra (2023). Bull Chem Soc Jpn.

[231] Roy R, Brouillac C, Jacques E, Quinton C, Poriel C (2024). Angew Chem, Int Ed.

[232] Ikeda K, Matsuo T, Yano K, Hayashi S (2024). Bull Chem Soc Jpn.

[233] Matoba Y, Uemura S, Funahashi M (2023). Bull Chem Soc Jpn.

[234] Han M J, Wei D, Kim Y H, Ahn H, Shin T J, Clark N A, Walba D M, Yoon D K (2018). ACS Cent Sci.

[235] Wang C-I, Maier J C, Jackson N E (2024). Chem Sci.

[236] Martínez-Bueno A, Martín S, Ortega J, Folcia C L, Termine R, Golemme A, Giménez R, Sierra T (2024). Chem Mater.

[237] Seki A, Funahashi M, Aoki K (2023). Bull Chem Soc Jpn.

[238] Kresge C T, Leonowicz M E, Roth W J, Vartuli J C, Beck J S (1992). Nature.

[239] Beck J S, Vartuli J C, Roth W J, Leonowicz M E, Kresge C T, Schmitt K D, Chu C T W, Olson D H, Sheppard E W, McCullen S B (1992). J Am Chem Soc.

[240] Zhang X, Li L, Chen Y, Valenzuela C, Liu Y, Yang Y, Feng Y, Wang L, Feng W (2024). Angew Chem, Int Ed.

[241] Raza H A, Karakaya I, Dag Ö (2023). ACS Appl Energy Mater.

[242] Yu H, Gold J I, Wolter T J, Bao N, Smith E, Zhang H A, Twieg R J, Mavrikakis M, Abbott N L (2024). Adv Mater (Weinheim, Ger).

[243] Seki T (2024). Bull Chem Soc Jpn.

[244] Brand R, Lunkenheimer P, Loidl A (2002). J Chem Phys.

[245] Das S, Mondal A, Reddy C M (2020). Chem Soc Rev.

[246] Nishikawa K, Fujii K (2023). Bull Chem Soc Jpn.

[247] Nishikawa K, Fujii K, Matsumoto K, Abe H, Yoshizawa-Fujita M (2024). Bull Chem Soc Jpn.

[248] Hirotsu Y, Sekiguchi R, Takeoka Y, Rikukawa M, Yoshizawa-Fujita M (2024). Bull Chem Soc Jpn.

[249] Ariga K, Akakabe S, Sekiguchi R, Thomas M L, Takeoka Y, Rikukawa M, Yoshizawa-Fujita M (2024). ACS Omega.

[250] Gibson V C, Spitzmesser S K (2003). Chem Rev.

[251] Li F, Suzuki R, Gao T, Xia X, Isono T, Satoh T (2023). Bull Chem Soc Jpn.

[252] Gao R-T, Li S-Y, Liu B-H, Chen Z, Liu N, Zhou L, Wu Z-Q (2024). Chem Sci.

[253] Sato K, Gon M, Tanaka K, Chujo Y (2024). Bull Chem Soc Jpn.

[254] Inokuma Y (2024). Bull Chem Soc Jpn.

[255] Lvov Y, Ariga K, Ichinose I, Kunitake T (1995). J Am Chem Soc.

[256] Kotov N A, Dekany I, Fendler J H (1995). J Phys Chem.

[257] Ariga K, Hill J P, Ji Q (2007). Phys Chem Chem Phys.

[258] Rydzek G, Ji Q, Li M, Schaaf P, Hill J P, Boulmedais F, Ariga K (2015). Nano Today.

[259] Borges J, Zeng J, Liu X Q, Chang H, Monge C, Garot C, Ren K-f, Machillot P, Vrana N E, Lavalle P (2024). Adv Healthcare Mater.

[260] Lorenzo A, Marmisollé W A, Maza E M, Ceolín M, Azzaroni O (2018). Phys Chem Chem Phys.

[261] Arnim V v, Finkelmann H, Dobarro A, Velasco D (1996). Macromol Chem Phys.

[262] Severing K, Stibal-Fischer E, Hasenhindl A, Finkelmann H, Saalwächter K (2006). J Phys Chem B.

[263] Li Y, Shan X, Li S, Wang J, Li Z, Wang Z, Li X, Hong W, Li M, Ma Y (2023). Angew Chem, Int Ed.

[264] Yang J, Sekizawa Y, Shi X, Ijiro K, Mitomo H (2024). Bull Chem Soc Jpn.

[265] Makio H, Terao H, Iwashita A, Fujita T (2011). Chem Rev.

[266] Tanaka T, Iwamoto S, Aso Y (2023). Bull Chem Soc Jpn.

[267] Nakano T, Imoto H, Naka K (2024). Bull Chem Soc Jpn.

[268] Okamoto H, Sogabe A, Honda S (2024). Commun Chem.

[269] Kitao T (2024). Bull Chem Soc Jpn.

[270] Kubota K (2023). Bull Chem Soc Jpn.

[271] Chen L, Nixon R, De Bo G (2024). Nature.

[272] Kim Y, Iimura K-i, Tamaoki N (2024). Bull Chem Soc Jpn.

[273] Watabe T, Otsuka H (2024). Macromolecules.

[274] Yamamoto T, Takahashi A, Otsuka H (2024). Bull Chem Soc Jpn.

[275] Mártire A P, Segovia G M, Azzaroni O, Rafti M, Marmisollé W (2019). Mol Syst Des Eng.

[276] Cortez M L, Lorenzo A, Marmisollé W A, von Bilderling C, Maza E, Pietrasanta L, Battaglini F, Ceolín M, Azzaroni O (2018). Soft Matter.

[277] Huang J, Lin S, Bai X, Li W, Zhang R, Miao C, Zhang X, Huang Z, Chen M, Weng S (2022). ACS Appl Mater Interfaces.

[278] Ishii M, Yamashita Y, Watanabe S, Ariga K, Takeya J (2023). Nature.

[279] Li L, Jia S, Yue S, Wang C, Qiu H, Ji Y, Cao M, Zhang D (2024). Green Chem.

[280] Fu Z, Liu H, Lyu Q, Dai J, Ji C, Tian Y (2024). Chem Eng J.

[281] Liang S, Feng H, Chen N, Wang B, Hu M, Huang X, Yang K, Gu Y (2024). Bull Chem Soc Jpn.

[282] Kubota R (2023). Bull Chem Soc Jpn.

[283] Sekine Y, Nankawa T (2023). Bull Chem Soc Jpn.

[284] Pedige M P H, Sugawara A, Uyama H (2024). Bull Chem Soc Jpn.

[285] Song S-N, Zhao X-L, Yang X-C, Ding Y, Ren F-D, Pang X-Y, Li B, Hu J-Y, Chen Y-Z, Gao W-W (2024). ACS Appl Mater Interfaces.

[286] Roopsung N, An T L H, Sugawara A, Asoh T-A, Hsu Y-I, Uyama H (2023). Bull Chem Soc Jpn.

[287] Wang Y, Li P, Cao S, Liu Y, Gao C (2023). Nanoscale.

[288] Ito K (2007). Polym J.

[289] Liu C, Morimoto N, Jiang L, Kawahara S, Noritomi T, Yokoyama H, Mayumi K, Ito K (2021). Science.

[290] Tsuruta T (2010). J Biomater Sci, Polym Ed.

[291] Tanaka M, Hayashi T, Morita S (2013). Polym J.

[292] Nishimura S-n, Tanaka M (2023). Bull Chem Soc Jpn.

[293] Roy B, Govindaraju T (2024). Bull Chem Soc Jpn.

[294] Zhang R, Wang Y, Yang G (2022). Polymers (Basel, Switz).

[295] Zhang C, Guo Q, Tong Z, Chen S, Mao Z, Yu Y (2022). J Colloid Interface Sci.

[296] Marin E, Tapeinos C, Sarasua J R, Larrañaga A (2022). Adv Colloid Interface Sci.

[297] Fu C, Wang Z, Zhou X, Hu B, Li C, Yang P (2024). Chem Soc Rev.

[298] Ariga K, Onda M, Lvov Y, Kunitake T (1997). Chem Lett.

[299] Vranckx C, Lambricht L, Préat V, Cornu O, Dupont-Gillain C, vander Straeten A (2022). Langmuir.

[300] Komiyama M, Yoshimoto K, Sisido M, Ariga K (2017). Bull Chem Soc Jpn.

[301] Liu J, Wang R, Zhou H, Mathesh M, Dubey M, Zhang W, Wang B, Yang W (2022). Nanoscale.

[302] Sun J, Ge J, Liu W, Lan M, Zhang H, Wang P, Wang Y, Niu Z (2014). Nanoscale.

[303] Ma N, Qiu W, Wei G, Zhang J, Yu H, Kong J, Zhang X (2024). Chem Eng J.

[304] Yu Z, Tang J, Xu M, Wu D, Gao Y, Zeng Y, Liu X, Tang D (2024). Anal Chem (Washington, DC, U S).

[305] Kalyana Sundaram S d, Hossain M M, Rezki M, Ariga K, Tsujimura S (2023). Biosensors.

[306] Bae S, Kim M, Jo N, Kim K M, Lee C, Kwon T-H, Nam Y S, Ryu J (2024). ACS Appl Mater Interfaces.

[307] Wang T, Fei J, Dong Z, Yu F, Li J (2024). Angew Chem, Int Ed.

[308] Li Z, Xu X, Yu F, Fei J, Li Q, Dong M, Li J (2022). Angew Chem, Int Ed.

[309] Hyman A A, Weber C A, Jülicher F (2014). Annu Rev Cell Dev Biol.

[310] Yue T, Zhang F, Wei Y, Wang Z (2024). Nano Today.

[311] Choi J, Rafiq N M, Park D (2024). Trends Biochem Sci.

[312] Zhou P, Xing R, Li Q, Li J, Yuan C, Yan X (2023). Matter.

[313] Mathesh M, Bhattarai E, Yang W (2022). Angew Chem, Int Ed.

[314] Song J, Murata T, Tsai K-C, Jia X, Sciortino F, Ma R, Yamauchi Y, Hill J P, Shrestha L K, Ariga K (2022). Adv Mater Interfaces.

[315] Ruffieux P, Wang S, Yang B, Sánchez-Sánchez C, Liu J, Dienel T, Talirz L, Shinde P, Pignedoli C A, Passerone D (2016). Nature.

[316] Clair S, de Oteyza D G (2019). Chem Rev.

[317] Li D, Silveira O J, Matsuda T, Hayashi H, Maeda H, Foster A S, Kawai S (2024). Angew Chem, Int Ed.

[318] Chen G, Isegawa M, Koide T, Yoshida Y, Harano K, Hayashida K, Fujita S, Takeyasu K, Ariga K, Nakamura J (2024). Angew Chem, Int Ed.

[319] Jia Y, Yan X, Li J (2022). Angew Chem, Int Ed.

[320] Yang T, Skirtach A G (2025). Materials.

[321] Aleena P A, Singh G, Vidyasagar D, Kumar P, Ahmed M I, Bahadur R, Sathish C, Sajan D, Vinu A (2025). Prog Mater Sci.

[322] Chen G, Koide T, Nakamura J, Ariga K Small Methods.

[323] Ariga K (2025). Materials.

[324] Mao X, Bischofberger I, Hosoi A E (2024). Proc Natl Acad Sci U S A.

[325] Yu W, Lin K-Y, Boyle D T, Tang M T, Cui Y, Chen Y, Yu Z, Xu R, Lin Y, Feng G (2025). Nat Chem.

[326] Nishitani S, Ao K, Jalil A, Arias-Soto O I, Moudi A, Chen F, Biyani A, Muppirala P N, Landry M P (2025). Proc Natl Acad Sci U S A.

[327] Ramani N, Hwang J, Anderson A J, Delgado J, Hernández-López L, Figg C A, Winegar P H, Mirkin C A (2025). J Am Chem Soc.

[328] Saito N, Nawachi A, Kondo Y, Choi J, Morimoto H, Ohshima T (2023). Bull Chem Soc Jpn.

[329] Nakaguro K, Mitsuta Y, Koseki S, Oshiyama T, Asada T (2023). Bull Chem Soc Jpn.

[330] Price C C, Li Y, Zhou G, Younas R, Zeng S S, Scanlon T H, Munro J M, Hinkle C L (2024). Nano Lett.

[331] Yospanya W, Matsumura A, Imasato Y, Itou T, Aoki Y, Nakazawa H, Matsui T, Yokoyama T, Ui M, Umetsu M (2024). Bull Chem Soc Jpn.

[332] Liu P, Zhu X, Ran X, Bi H, Huang X, Gu N (2025). Coord Chem Rev.

[333] Ramprasad R, Batra R, Pilania G, Mannodi-Kanakkithodi A, Kim C (2017). npj Comput Mater.

[334] Ootahara T, Hatakeyama-Sato K, Thomas M L, Takeoka Y, Rikukawa M, Yoshizawa-Fujita M (2024). ACS Appl Electron Mater.

[335] Sivan D, Zafar S, Rohit R V, Vipin R R, Satheeshkumar K, Raj V, Moorthy K, Misnon I I, Ramakrishna S, Jose R (2024). Mater Today Sustainability.

[336] Chaikittisilp W, Yamauchi Y, Ariga K (2022). Adv Mater (Weinheim, Ger).

[337] Oviedo L R, Oviedo V R, Martins M O, Fagan S B, da Silva W L (2022). J Nanopart Res.

